# In-silico pharmacological insights into the therapeutic potential of microRNAs for microplastic-associated cancers

**DOI:** 10.3389/fcell.2025.1699693

**Published:** 2025-11-25

**Authors:** Akmaral Baspakova, Afshin Zare, Nadiar M. Mussin, Nader Tanideh, Kulyash R. Zhilisbayeva, Ramazon Safarzoda Sharoffidin, Roza Suleimenova, Gulden Yelgondina, Akmeiir E. Kaliyeva, Aigerim A. Umbetova, Ainur Zinaliyeva, Amin Tamadon

**Affiliations:** 1 Department of Epidemiology, West Kazakhstan Marat Ospanov Medical University, Aktobe, Kazakhstan; 2 Drug Discovery and Development Industry, School of Pharmacy, Taipei Medical University, Taipei, Taiwan; 3 Department of Surgery No. 2, West Kazakhstan Marat Ospanov Medical University, Aktobe, Kazakhstan; 4 Stem Cells Technology Research Center, Shiraz University of Medical Sciences, Shiraz, Iran; 5 Department of Pharmacology, Medical School, Shiraz University of Medical Sciences, Shiraz, Iran; 6 Department of Languages, West Kazakhstan Marat Ospanov Medical University, Aktobe, Kazakhstan; 7 Department of Pharmaceutical Technology, Avicenna Tajik State Medical University, Dushanbe, Tajikistan; 8 Department of Public Health and Hygiene, Astana Medical University, Astana, Kazakhstan; 9 School of General Medicine-2, Asfendiyarov Kazakh National Medical University, Almaty, Kazakhstan; 10 Department of Microbiology, Virology and Immunology, West Kazakhstan Marat Ospanov Medical University, Aktobe, Kazakhstan; 11 Department for Scientific Work, West Kazakhstan Marat Ospanov Medical University, Aktobe, Kazakhstan; 12 Department of General Medical Practice No. 2, West Kazakhstan Marat Ospanov Medical University, Aktobe, Kazakhstan; 13 Department of Natural Sciences, West Kazakhstan Marat Ospanov Medical University, Aktobe, Kazakhstan

**Keywords:** microplastics, microRNAs, cancer, therapy resistance, *in silico*, RNAhybrid

## Abstract

Microplastics (MPs) are increasingly implicated in cancer biology through effects on gene expression, stress responses, and treatment susceptibility; however, causal links remain provisional. We systematically screened PubMed and Google Scholar (through September 2025) to identify cancer-related genes reported to be altered by MP exposure and then evaluated microRNAs (miRNAs) with anticancer activity that may target those genes. Mature miRNA sequences were retrieved from RNAcentral and assessed against MP-altered genes using RNAhybrid for target-site prediction and minimum free-energy (mfe) hybridization. MPs were reported to modulate genes across multiple tumor types—including breast, gastric, liver, lung, colorectal, cervical, pancreatic, and skin. In silico analyses identified candidate miRNAs with favorable mfe values for these targets, including miR-483-3p, miR-365, miR-331-3p, miR-138-5p, miR-760, miR-1-3p, miR-665, miR-490-3p, miR-370-3p, miR-520a, miR-638, miR-559, miR-532-3p, miR-593-5p, and miR-29b. These interactions suggest putative avenues to counter MP-associated oncogenic programs and therapy resistance. Because mfe predictions do not establish functional regulation, all findings should be interpreted as hypothesis-generating. Priorities for validation include reporter assays, gene/protein modulation, phenotypic rescue, and *in vivo* testing in MP-exposed models. Collectively, our results nominate miRNAs as candidate tools to interrogate and potentially mitigate MP-associated carcinogenic mechanisms.

## 1 Introduction

Microplastics (MPs) are conventionally defined as plastic particles <5 mm (5,000 µm) in diameter, while nanoplastics (NPs) are generally <1 µm. For the purposes of this review, we focused on the range of 1–5,000 μm, consistent with commonly reported experimental studies (Hale et al., 2020). Among these, human health is one of the most critical concerns. Studies have confirmed the presence of MPs in the human body worldwide, where they can accumulate in various types of cells (Vethaak and Legler, 2021). This accumulation may lead to several adverse health outcomes, including gut microbiota disruption and respiratory disorders (Winiarska et al., 2024).

Microplastics (MPs < 5 mm) have been detected in human tissues and may perturb cancer-related pathways including oxidative stress, lipid metabolism, inflammation, and drug transport. Studies report that MPs can upregulate efflux transporters (ABCB1/ABCG2) and alter chemotherapeutic susceptibility (Rosellini et al., 2023), enhance metastatic features in breast cancer (Park et al., 2023), promote therapy resistance via ASGR2 in gastric cancer (Kim et al., 2022), and aggravate radiation-induced intestinal injury (Chen Y. et al., 2024). Despite these observations, mechanisms remain poorly defined.

One of the most concerning potential health impacts of MPs is cancer. Previous studies have suggested MPs as possible contributors to carcinogenic processes, but the evidence remains preliminary and largely associative (Kumar et al., 2024). In addition to initiating tumorigenesis through mechanisms such as DNA damage (Hu et al., 2022), may also influence the response of cancer cells to anti-cancer therapies, potentially contributing to drug resistance (Kim et al., 2022). These findings underscore the need to explore novel strategies for cancer treatment.

Therefore, scientists have attempted to develop various kinds of anti-cancer therapeutic agents in recent years (Sun et al., 2023). Recent research has focused on developing innovative anticancer strategies, including advanced drug platforms (Kaliyev et al., 2024), stem-cell–based therapies (Kaliyev et al., 2024), and public education initiatives (Barani, 2024). Among these, microRNAs (miRNAs) have emerged as promising anti-cancer agents.

miRNAs are ∼22-nucleotide non-coding RNAs that guide Argonaute complexes to complementary mRNA regions, leading to mRNA degradation or translational repression. Each miRNA regulates many targets, allowing broad control of oncogenic networks involving proliferation, apoptosis, invasion, and drug resistance (Szczepanek et al., 2022). These regulatory properties make miRNAs attractive therapeutic candidates for MP-associated cancers. miRNAs possess several advantageous properties, including the ability to regulate cancer-related pathways, modulate drug sensitivity, deliver therapeutic molecules, and enable personalized treatment approaches (Szczepanek et al., 2022), making them strong candidates for treating MP-associated cancers. However, there is still a lack of comprehensive understanding regarding the potential of miRNAs to treat MP- associated tumors and the molecular mechanisms through which they exert anti-cancer effects. Most previous studies have focused on how MPs alter miRNA expression and function (Chen T. et al., 2024).

Therefore, the present study aims to investigate the therapeutic potential of miRNAs in MP-associated cancers through an *in silico* analysis. Additionally, this review explores possible molecular mechanisms underlying the anti-cancer activity of miRNAs and offers insights for future *in-vivo* and *in-vitro* research to further clarify their role in treating MP-associated malignancies.

## 2 Materials and methods

### 2.1 Literature identification and selection

**FIGURE 1:**
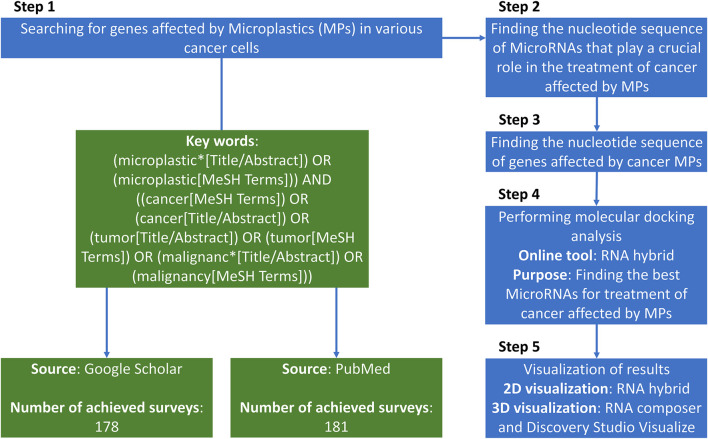
The total paradigm of the searching strategy of the present study.

We queried PubMed and Google Scholar from database inception to 30 September 2025 (Figure 1). Example search string (PubMed): (microplastic* OR nanoplastic* OR “plastic-related”) AND (cancer OR tumor OR carcinoma OR leukemia) AND (gene OR transcript* OR “drug resistance” OR efflux OR MAPK OR ABCB1 OR ABCG2) and for miRNAs: (microRNA OR miRNA) AND (anticancer OR tumor suppress* OR apoptosis OR chemosensit*) AND (breast OR gastric OR liver OR lung OR colorectal OR cervical OR pancreatic OR melanoma).

Inclusion criteria: (i) primary *in vitro*/*in vivo*/clinical studies in human cancer models or human tissues reporting MP exposure (or plastic-related compounds) and gene/protein/pathway changes; (ii) peer-reviewed; (iii) English. Exclusion: reviews, editorials, non-human non-cancer models, studies reporting expression changes without cancer relevance, or miRNAs lacking anticancer functional evidence. Two reviewers independently screened titles/abstracts and full texts; disagreements were resolved by a third reviewer.

### 2.2 Sequence sources and target selection

Mature miRNA sequences were retrieved from RNAcentral; human gene sequences (mRNA/UTR/coding region where available) were obtained from NCBI Gene/RefSeq. We evaluated genes previously reported as MP-altered in cancer contexts (Tables 1–3). (Chen T. et al., 2024).

**TABLE 1:** Genes that are affected by microplastics (MPs) in various types of cancer cells.

Microplastic	Type of cancer cell	Cancer type	Affected gene	Effect of microplastics on gene	The final impact on the cancer cell	Reference
MP	CEM/ADR5000 cells	Leukemia	ABCB1 ABCG2	Inhibition	Increasing cytotoxicity	Rosellini et al. (2023)
	MDA-MB-231-BCRP cells	Breast cancer			Disruption of detoxification pathways	
					Heightening cell susceptibility to xenobiotics	
PMMA	HepG2	Liver cancer	HO-1	Upregulation	Increasing oxidative stress	Boran et al. (2024)
					Promotion of inflammation	
			PPARγ		Increasing lipid accumulation	
			LXR-α		Disruption of lipid homeostasis	
					Increasing inflammation	
			FABP1	Downregulation	Promoting inflammation	
					Promoting lipid accumulation	
			PPARα	Downregulation	Mitochondrial damage	
					Lipid metabolism disruption	
Polythene	SCL-1	Skin cancer	NLRP3	Increased expression	Enhancing proliferation	Wang et al. (2023)
	A431				Enhancing the inflammatory response	
PP	MDA-MB-231	Breast cancer	TMBIM6	Increased expression	Enhancing cell cycle progression	Park et al. (2023)
			AP2M1	Increased expression	Increasing secretion of pro-inflammatory cytokine IL-6	
			PTP4A2	Increased expression	Promoting metastatic features	
			FTH1	Reduced expression		
PS	NCI-N87	Gastric cancer	CD44	Increased expression	Multidrug resistance	Zhao et al. (2024), Brynzak-Schreiber et al. (2024), Li et al. (2023), Yan et al. (2020)
			ASGR2	Increased expression	Enhancing cancer hallmarks	
					Enhancing proliferation	
					Enhancing migration	
					Drug resistance	
		Colorectal cancer cells	MUC2	Reduced expression	Loss of mucosal barrier protection	
					Increasing inflammation-driven carcinogenesis	
					Promotion of colorectal cancer risk	
		Lung cancer cells	TIM4	Potential binding and internalization of PS microplastics	Altering the tumor immune microenvironment	
		Cervical cancer cells			Immune suppression and modulation	
		Colorectal cancer cells			Tumor progression	
		Gastric cancer cells				
		Pancreatic cancer cells				
	AGS	Gastric cancer cells	Bax	Overexpression	Decreasing cell viability	
					Inducing apoptosis	
					Inducing DNA damage	
PTFE	A549	Lung cancer	MAPK Family	Activating	Induction of oxidative stress	K et al. (2023)
			NLRP3	Reduced expression	Induction of an inflammatory response	
			BCL2	Increased expression	Induction of carcinogenic processes	

A431, human epidermoid carcinoma cell line; ABCB1, ATP-binding cassette sub-family B member 1; ABCG2, ATP-binding cassette sub-family G member 2; AGS, adenocarcinoma gastric cancer cells; AP2M1, adaptor-related protein complex 2 subunit mu 1; ASGR2, asialoglycoprotein receptor 2; A549, human lung carcinoma cell line; Bax, Bcl-2-associated X protein; BCL2, B-cell lymphoma 2; CEM/ADR5000 cells, doxorubicin-resistant human acute lymphoblastic leukemia cells; CD44, cluster of differentiation 44; FABP1, fatty acid-binding protein 1; FTH1, ferritin heavy chain 1; HO-1, heme oxygenase 1; LXR-α, liver X receptor alpha; MAPK, family, mitogen-activated protein kinase family; MDA-MB-231, human breast adenocarcinoma cell line; MDA-MB-231-BCRP, cells, breast cancer resistance protein-overexpressing MDA-MB-231, cells; MP, microplastic; MUC2, mucin 2; NCI-N87, human gastric carcinoma cell line; NLRP3, NACHT, LRR, and PYD, domains-containing protein 3; PMMA, polymethyl methacrylate; PP, polypropylene; PPARα, peroxisome proliferator-activated receptor alpha; PPARγ, peroxisome proliferator-activated receptor gamma; PS, polystyrene; PTFE, polytetrafluoroethylene; PTP4A2, protein tyrosine phosphatase type IVA, member 2; SCL-1, squamous cell carcinoma cell line; TMBIM6, transmembrane BAX, inhibitor motif-containing 6; TIM4, T-cell immunoglobulin and mucin domain-containing protein 4.

**TABLE 2:** Detailed information about gene function and molecular mechanisms that are affected by MPs, based on previous studies.

Gene	The total function of genes in cancer	Type of cancer	Function of the gene in cancer cells	The molecular mechanism by which genes exert their function in cancer	Reference
ABCB1	+ Substance transportation + Role in Vascular Transport + Vascular Processes in the Circulatory System + Ion Channel Interactions	+ Breast cancer + Leukemia	Preventing the accumulation of toxic substances inside cells	Actively pumps chemotherapeutic drugs out of cancer cells, thereby reducing intracellular drug accumulation	Miao et al. (2017), Altamura et al. (2022)
			Preventing the accumulation of certain chemotherapeutic drugs		
			Controlling the transport of substances between blood and tissues	Contributes to MDR by lowering the efficacy of various anticancer drugs	
			Reducing drug penetration into certain tissues	Increased efflux of chemotherapeutic drugs, reducing their cytotoxic effects	
			Impacting pharmacokinetics and limiting drug efficacy in cancer therapy		
			Influencing the selective permeability of blood vessels	Barrier Integrity and Selectivity: By regulating the efflux of molecules, P-gp contributes to maintaining the selective permeability of vascular barriers	
			Affecting drug distribution and clearance	Drug Resistance in Circulatory Tumors: P-gp impacts drug efficacy by limiting the systemic retention of drugs within the vascular environment	
			Regulating apoptosis	P-gp may regulate or interact with ion channels, contributing to cellular responses and influencing apoptosis and drug resistance	
			Regulating proliferation	Facilitating cellular adaptation to stress conditions, such as chemotherapy exposure	
ABCG2	+ Response to the drug + Substance transportation	+ Breast Cancer + Leukemia	Increasing drug efflux	Reducing intracellular retention of chemotherapeutic agents	Wang et al. (2020), Franczyk et al. (2022)
				Transporting a wide range of chemotherapeutic agents and limiting their cytotoxic effects in cancer cells	
			Acting as a barrier in tissues like the blood-brain barrier, liver, and intestines	Regulating the bioavailability and toxicity of drugs	
AP2M1	Vesicle coat	Breast Cancer	Promoting intracellular trafficking	Coating vesicles during endocytosis	Shin et al., 2021; Münz (2020), Xue et al. (2021)
			Promoting cancer progression	Facilitating the recruitment of cargo proteins to clathrin-coated vesicles	
				Maintaining cellular homeostasis	
				Regulating the availability of key signaling molecules	
			Promoting tumor growth and progression	Influencing the internalization and trafficking of cancer-related receptors such as EGFR	
	Antigen Processing		Playing a role in endocytosis	Modulating cancer cell signaling	
				Modulating cancer cell nutrient uptake	
				Modulating cancer cell receptor recycling or degradation	
				Curbing oncogenic pathways	
			Internalization of MHC class I molecules	Regulating the trafficking and recycling of MHC molecules	
				Ensuring proper antigen display to T cells	
	receptor-mediated endocytosis		Promoting cancer progression	Increasing receptor endocytosis	
			Modulating cancer cell proliferation and survival pathways	Regulating CME	
				Mediating the trafficking of receptors such as EGFR	
ASGR2	Regulation of immune response	Gastric Cancer	Influencing the tumor microenvironment	+ Targeting glycoproteins with terminal galactose or N-acetylgalactosamine residues for degradation + Glycoprotein recycling + Immune regulation	Xue et al. (2021)
			Promoting tumor metastasis		
			Worsening of tumor prognosis		
			Increasing tumor aggressiveness		
			Increasing tumor survival		
Bax		Gastric cancer	Inducing apoptosis in cancer cells	Collapsing the mitochondrial membrane potential, which results in the release of cytochrome C, which leads to apoptosis	Shabani et al. (2020), Shen et al. (2023)
				Activation caspases 3, 7, and 9	
BCL2	Regulation of the extrinsic apoptotic signaling pathway	Lung cancer	Preventing cancer cell death	+ Inhibiting the pro-apoptotic protein Bax + Inhibiting the activation of caspases 3, 7, and 9 + Inhibiting the activation of the cleavage of PARP	Ramkumar et al. (2023), Alam et al. (2022a), Alam et al. (2022b)
			Promoting cancer cell survival		
			Promoting resistance to chemotherapy and radiation		
			Preventing the induction of apoptosis		
			Avoiding programmed cell death		
			Blocking the activation of downstream caspases	Preventing the release of cytochrome c from mitochondria	
			Promoting cancer growth		
			Promoting the persistence of cancer cells		
CD44	Cell-matrix adhesion	Gastric cancer	Enhancing antioxidant defense	Stabilizing the xCT cystine transporter	Jang et al., 2011; Zavros (2017)
			Reducing oxidative stress		
			Enhancing the antioxidant defense of cancer stem cells		
			Tumor cell survival		
			Promoting tumor growth		
			Tumor initiation	Interaction with extracellular matrix components such as hyaluronan	
			Tumor progression	Involvement in signaling pathways like c-Met activation	
FABP1	Lipid oxidation	Liver cancer	Enhancing tumor progression	Interacting with PPARG in TAMs to promote FAO	Tang et al. (2023)
			Maintaining the M2 phenotype of TAMs		
			Immune suppression		
FTH1	Iron ion homeostasis	Breast Cancer	Iron storage	Activating, binding, and stabilizing the tumor suppressor p53 under oxidative stress	Di Sanzo et al. (2020), Jia et al. (2024)
			Oxidative damage protection		
			Regulation of angiogenesis		
			Inhibiting ferroptosis	Chelating ferrous iron and reducing ROS accumulation	
			Inhibiting chemotherapy resistance		
			Stabilizing high levels of antioxidant capacity in breast cancer cells		
HO-1	Heme metabolic process	Liver cancer	Reducing oxidative stress	Playing a role in the Nrf2 signaling pathway	Alharbi et al. (2022)
			Creating Cytoprotective Effects	Facilitating the production of biliverdin, CO, and free iron from heme	
			Modulating tumor cell proliferation	Anti-apoptotic and antioxidant properties​	
			Enhancing cancer cell resistance to treatment	Activation of survival pathways	
			Enhancing tumor progression	Suppression of apoptosis​	
			Enhancing metastasis		
LXR-α	Fatty acid biosynthetic process	Liver cancer	Lipid accumulation	Modulating downstream target genes, such as SREBP-1c, PPARγ, and FAS	Han et al. (2023)
	Inflammation		Inhibiting Cancer Progression		
	Immune responses		Inhibiting tumor cell proliferation	Upregulation of genes like SOCS3 and suppression of oncogenes like FOXM1	
			Inducing cell cycle arrest		
			Inducing apoptosis		
			Reducing tumor invasiveness​	Interacting with the Wnt/β-catenin and NF-κB signaling pathways	
			Reducing tumor migration		
MAPK Family		Lung cancer	Increasing cancer cell proliferation	Activation of Ras/Raf/MEK/ERK cascade	Jain et al. (2021), Zhou et al. (2020a)
			Increasing cancer cell metastasis		
			Promoting cancer cell resistance to targeted therapies		
			Promoting cancer cell proliferation	Activation of receptor α4β1 integrin leads to phosphorylation of MAPK components such as JNK and p38	
			Promoting cancer cell migration		
			Promoting cancer cell invasion		
MUC2	Glycoprotein biosynthetic process	Colorectal cancer cells	Regulating immune response and inflammation	+ Decreasing IL-6 secretion + Inhibiting the STAT3 and Chk2 signaling pathway + Activation of CREB phosphorylation + Upregulation of E-cadherin	Hsu et al. (2017)
			Decreasing tumor cell migration		
			Decreasing EMT		
			Decreasing metastasis		
			Decreasing cancer progression		
NLRP3	Regulation of the inflammatory response	+ Skin Cancer + Lung cancer	Inducing inflammation	Activation of NLRP3, ASC, and caspase-1 which leads to Activating IL-1β and IL-18	Ciazyn et al. (2020)
	Regulation of cytokine production is involved in the inflammatory response		Increasing tumor growth		
			Increasing tumor angiogenesis		
			Increasing tumor immune evasion mechanisms		
			Inducing DNA damage		
			Suppressing apoptosis		
			Creating a tumor-promoting environment		
PPARα	Regulation of the inflammatory response	Liver cancer	+ Increasing tumor cell proliferation + Decreasing tumor cell apoptosis + Increasing tumor cell invasiveness	Regulating lipid metabolism	Pan et al. (2024)
	Regulation of cytokine production is involved in the inflammatory response			Regulating glucose metabolism	
				Regulating oxidative stress	
				Regulating inflammation	
				Interacting with several signaling pathways like NF-κB	
PPARγ	Transcription coactivator activity	Liver cancer	Inducing the transcription of genes involved in anti-inflammatory, anti-fibrotic, and anti-oxidative responses	Binding with the RXR to specific DNA sequences called PPREs	Pan et al. (2024), Ishtiaq et al. (2022)
	Ligand-activated transcription factor activity		Anti-inflammatory and anti-fibrotic effects	+ Inhibiting the expression of TNF-α, IL-6, IL-1 through inhibiting the NF-κB signaling pathway + Promoting the expression of anti-inflammatory cytokines like IL-10, TGF-β + Inducing the expression of antioxidant enzymes such as SOD and catalase + reduces ROS levels + Limiting DNA damage and oxidative stress	
	Regulation of the inflammatory response		Inhibiting hepatic fibrosis progression and inflammation		
	Regulation of cytokine production is involved in the inflammatory response		Suppressing tumor microenvironment remodeling		
			Decreasing cancer cell apoptosis		
			Suppressing ECM deposition in liver fibrosis	Suppressing the activation of HSCs	
			Enhancing glucose consumption		
			Enhancing lactate generation		
			Enhancing cancer cell proliferation		
PTP4A2		Breast Cancer	Promoting angiogenesis	Regulation of VEGF-A and DLL-4/Notch-1 signaling pathways	Yu et al. (2023)
			Enhancing tumor cell migration	Regulation ERK signaling pathway	
			Enhancing tumor cell invasion		
			Supporting oncogenesis	Interaction with CNNM3	
			Enhancing tumor progression and metastasis	Regulating intracellular magnesium concentration	
TIM4	Antigen processing and presentation	Lung cancer cells	Tumor progression	Binding to PtdSer on apoptotic cells, mediating their uptake by DCs	Liu et al. (2020), Wang et al. (2021), Astuti et al. (2024), Caronni et al. (2021)
	Leukocyte migration	Lung cancer cells	Tumor cell proliferation	Interacting with αvβ3 integrin via its Arg-Gly-Asp (RGD) motif	
		Colorectal cancer cells	Enhancing the EMT		
		Gastric cancer cells	Enhancing the migration of cancer cells		
			Enhancing the invasion of cancer cells		
			Recruitment of tumor-associated macrophages	Activating PI3K/AKT/mTOR signaling pathway	
			Activation of angiogenesis		
		Pancreatic cancer cells	Suppressing the immune system	Arg1 upregulation, which suppresses CD8^+^ T cell activity	
			Facilitating tumor immune evasion		
TMBIM6	Regulation of response to endoplasmic reticulum stress	Breast Cancer	Increasing chemo resistance	Elevating the levels of cytosolic ROS and calcium	Robinson et al. (2025), Junjappa et al. (2019), Shin et al. (2023)
	Apoptotic mitochondrial changes		Increasing cancer metastasis	Inducing paraptosis via ERAD II mechanisms	
			Increasing cancer progression	Activating lysosomal biogenesis	
			Enhancing migration and invasion	Activating the MAPK/ERK signaling pathway	
				Increasing the expression of mesenchymal markers, MMP-9, and Snail-1 and Snail-2	
			Cancer progression	PKC activation enhances Sp1-mediated TMBIM6 transcription	
			Supporting cellular processes like metastasis		
			Facilitating resistance to apoptosis		

Arg1, Arginase-1; CME, clathrin-mediated endocytosis; CO, carbon monoxide; DCs, dendritic cells; EGFR, epidermal growth factor receptor; EMT, epithelial-mesenchymal transition; ERK, Extracellular signal-Regulated Kinase; FAO, fatty acid oxidation; HSC, hepatic stellate cell; MDR, multidrug resistance; MEK, MAPK/ERK, kinase; MMPs, metalloproteinases; MMP-9, matrix metalloproteinase-9; PARP, poly-ADP, ribose polymerase; P-gp, P-glycoprotein; PKC, Protein kinase-C; PPARG, peroxisome proliferator-activated receptor gamma; PtdSer, phosphatidylserine; Raf, Rapidly Accelerated Fibrosarcoma; Ras, Rat Sarcoma virus; ROS, reactive oxygen species; SOD, superoxide dismutase; TAMs, tumor-associated macrophages.

**TABLE 3:** Various effects of MPs on the anti-cancer agents and the final effect caused by these impacts on cancer cells.

Type of MP	Type of MP-affected cancer	Affected anti-cancer agent	Effect of MP on anti-cancer agents	Effect of MP on cancer	Reference
Plastic-related compounds	Breast cancer cells	Doxorubicin	Altering the uptake and intracellular accumulation of doxorubicin	Increasing the cytotoxicity in cancer cells	Rosellini et al. (2023)
	Leukemia cells		Enhancing the intracellular concentration of doxorubicin	Affecting the therapeutic outcome	
PS	Abdominal and pelvic tumors	Radiotherapy	Aggravating radiation-induced intestinal injury	Enhancing the side effects of the anti-cancer approaches	Chen et al. (2024a), Yan et al. (2020), Zhou et al. (2020b)
				Decreasing the efficacy of radiotherapy	
	Gastric cancer	Bortezomib	+ Inducing drug resistance + Influencing the bioavailability and toxicity of Tetracycline	Enhancing cancer progression	
		Paclitaxel		Enhancing cancer proliferation	
		Gefitinib		Enhancing cancer migration	
		Lapatinib		Enhancing cancer invasion	
		Trastuzumab		Poor survival rates	
		Tetracycline		Enhancing the cytotoxicity of cancer cells	
				Reducing cancer cell viability	
				Increasing oxidative stress in cancer cells	
				Increasing cancer cell apoptosis	

### 2.3 In silico hybridization

We used RNAhybrid to predict target-site hybridization and minimum free energy (mfe). Default parameters were applied unless noted; where possible we scanned 3′UTRs preferentially and considered coding sequence if 3′UTR data were unavailable. For each gene, we screened multiple miRNAs with anticancer evidence and recorded the lowest mfe site per miRNA-gene pair. Lower (more negative) mfe was interpreted as more stable predicted binding. No single mfe threshold was used for exclusion; instead, candidates were prioritized by relative mfe within gene-specific comparisons (Chen T. et al., 2024; Jain et al., 2021). MFE criteria included: RNAhybrid minimum-free-energy (mfe) values were interpreted as strong (≤−20 kcal mol^−1^), suggestive (−15 to −19.9), and borderline (>−15). Pairs above −20 were retained only if supported by prior functional evidence and are flagged as low-priority hypothese.

### 2.4 Visualization and networks

**FIGURE 2:**
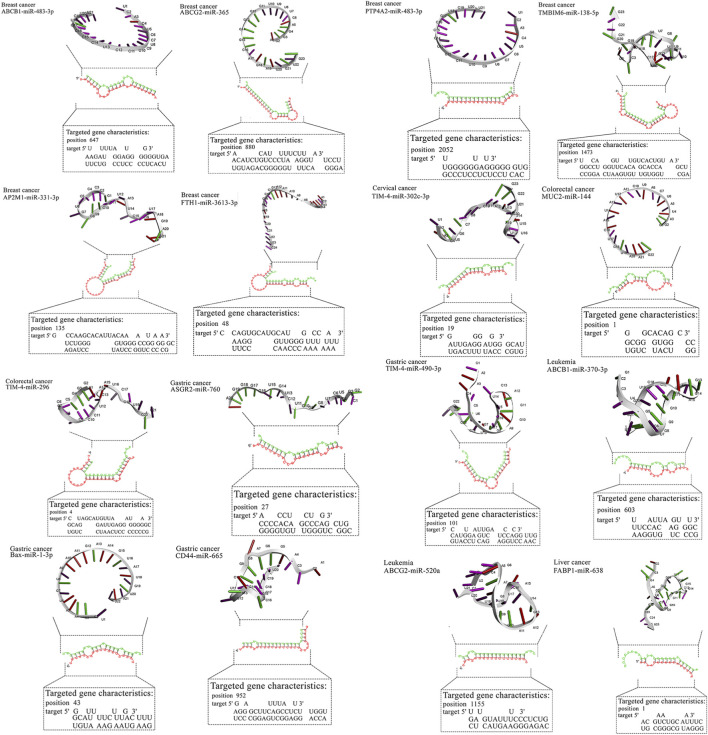
Detailed data about molecular interactions between microRNAs with anti-cancer activity and genes affected by MPs. The 3D structure at the top of the figure represents microRNA with the highest binding affinity toward the gene affected by MPs. The data about the targeted gene is depicted in the lower part of the figure. ABCB1, ATP Binding Cassette Subfamily B Member 1; ABCG2, ATP Binding Cassette Subfamily G Member 2; AP2M1, Adaptor Related Protein Complex 2 Subunit Mu 1; ASGR2, Asialoglycoprotein Receptor 2; Bax, BCL2 Associated X, Apoptosis Regulator; BCL2, B-cell CLL/lymphoma 2; BRAF, B-Raf Proto-Oncogene, Serine/Threonine Kinase; c-jun, Jun Proto-Oncogene, AP-1 Transcription Factor Subunit; CD44, CD44 Molecule (Indian Blood Group); ERK, Extracellular Signal-Regulated Kinase; FABP1, Fatty Acid Binding Protein 1; FTH1, Ferritin Heavy Chain 1; HO-1, Heme Oxygenase 1; JNK, c-Jun N-terminal Kinase; KRAS, KRAS Proto-Oncogene, GTPase; LXR-α, Liver X Receptor Alpha; MAP2K1, Mitogen-Activated Protein Kinase Kinase 1; MAP2K4, Mitogen-Activated Protein Kinase Kinase 4; MAPK14, Mitogen-Activated Protein Kinase 14; MUC2, Mucin 2; NLRP3, NLR Family Pyrin Domain Containing 3; NLRP3, NLR Family Pyrin Domain Containing 3; PPARα, Peroxisome Proliferator-Activated Receptor Alpha; PPARγ, Peroxisome Proliferator-Activated Receptor Gamma; PTP4A2, Protein Tyrosine Phosphatase Type IVA, Member 2; TMBIM6, Transmembrane BAX Inhibitor Motif Containing 6; TIM4, T-cell Immunoglobulin and Mucin-domain Containing-4; TIM4, T-cell Immunoglobulin and Mucin-domain Containing-4; TIM4, T-cell Immunoglobulin and Mucin-domain Containing-4; TIM4, T-cell Immunoglobulin and Mucin-domain Containing-4; TIM4, T-cell Immunoglobulin and Mucin-domain Containing-4.

**FIGURE 3:**
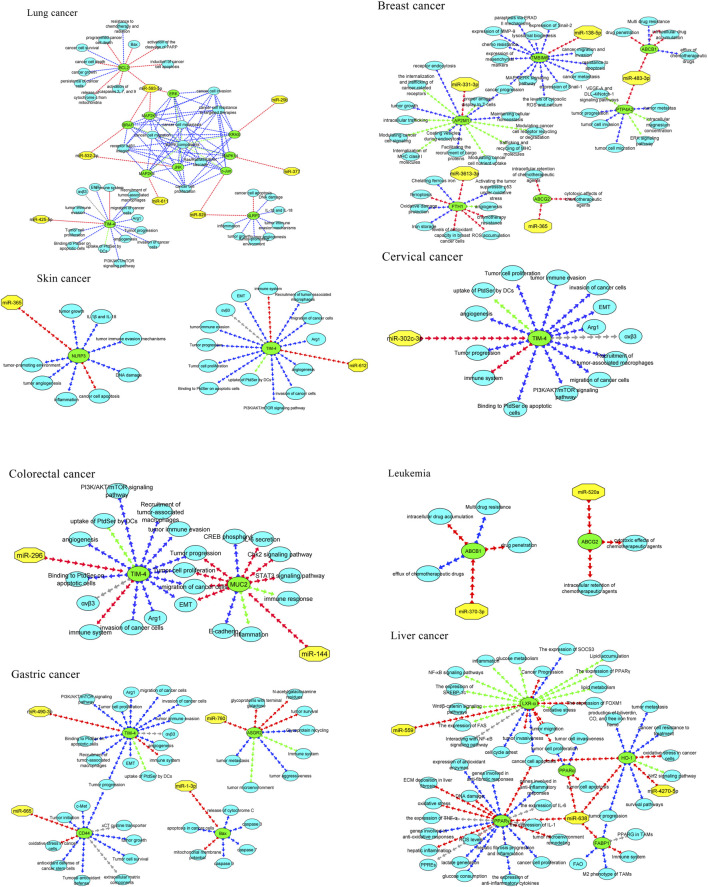
MicroRNAs with the in-silico capability to suppress genes in MPs-based cancers and their possible targeted molecular pathways. The blue, red, green, and gray arrows represent activation (or upregulation), inhibition (or downregulation), regulation, and interaction, respectively. Moreover, yellow hexagons, blue ovals, and green ovals demonstrate microRNAs, targeted genes, and pathways affected by targeted genes, respectively. ABCB1, ATP Binding Cassette Subfamily B Member 1; ABCG2, ATP Binding Cassette Subfamily G Member 2; AP2M1, Adaptor Related Protein Complex 2 Subunit Mu 1; ASGR2, Asialoglycoprotein Receptor 2; Bax, BCL2 Associated X, Apoptosis Regulator; BCL2, B-cell CLL/lymphoma 2; BRAF, B-Raf Proto-Oncogene, Serine/Threonine Kinase; c-jun, Jun Proto-Oncogene, AP-1 Transcription Factor Subunit; CD44, CD44 Molecule (Indian Blood Group); ERK, Extracellular Signal-Regulated Kinase; FABP1, Fatty Acid Binding Protein 1; FTH1, Ferritin Heavy Chain 1; HO-1, Heme Oxygenase 1; JNK, c-Jun N-terminal Kinase; KRAS, KRAS Proto-Oncogene, GTPase; LXR-α, Liver X Receptor Alpha; MAP2K1, Mitogen-Activated Protein Kinase Kinase 1; MAP2K4, Mitogen-Activated Protein Kinase Kinase 4; MAPK14, Mitogen-Activated Protein Kinase 14; MUC2, Mucin 2; NLRP3, NLR Family Pyrin Domain Containing 3; NLRP3, NLR Family Pyrin Domain Containing 3; PPARα, Peroxisome Proliferator-Activated Receptor Alpha; PPARγ, Peroxisome Proliferator-Activated Receptor Gamma; PTP4A2, Protein Tyrosine Phosphatase Type IVA, Member 2; TMBIM6, Transmembrane BAX Inhibitor Motif Containing 6; TIM4, T-cell Immunoglobulin and Mucin-domain Containing-4; TIM4, T-cell Immunoglobulin and Mucin-domain Containing-4; TIM4, T-cell Immunoglobulin and Mucin-domain Containing-4; TIM4, T-cell Immunoglobulin and Mucin-domain Containing-4; TIM4, T-cell Immunoglobulin and Mucin-domain Containing-4.

Two-dimensional pairing diagrams were exported from RNAhybrid. 3D miRNA cartoons were rendered in Discovery Studio Visualizer for illustrative purposes only. Cytoscape was used to depict miRNA–gene–pathway relationships (Figure 2). Representative 2D pairing plots, 3D illustrations, and network diagrams are shown in Figure 3.

### 2.5 Interpretive caveats

mfe predictions do not prove targeting; they require orthogonal validation (reporter assays with wild-type/mutant sites, miRNA gain/loss, protein readouts, and phenotypic rescue under MP exposure).

## 3 Results

### 3.1 MPs modulate cancer-relevant genes across tumor types

Across studies, MPs were linked to changes in efflux transporters (ABCB1, ABCG2), stress and survival mediators (TMBIM6, HO-1), immune modulators (TIM4), metabolic regulators (PPARα/γ, LXR-α, FABP1), and ECM/adhesion factors (CD44). In breast cancer models, polypropylene increased AP2M1, PTP4A2, and TMBIM6 while reducing FTH1, a pattern consistent with enhanced trafficking/ER-stress signaling and diminished iron-mediated tumor suppressive functions. In gastric cancer, polystyrene exposure associated with higher CD44 and ASGR2, aligning with stemness/adhesion and glycoprotein handling. In liver cancer, PMMA upregulated HO-1 and downregulated PPARα/FABP1, suggesting oxidative stress and lipid dysregulation. Lung cancer models exposed to PTFE implicated MAPK cascade genes and BCL2, consistent with proliferation and apoptosis evasion. Collectively, these reports converge on MP-associated activation of survival and resistance programs with tumor- and polymer-specific nuances. An overview of MP-altered genes by tumor type is summarized in Figure 3 (Table 1).

### 3.2 Functional roles of MP-altered genes

Table 2 maps each gene to cancer functions and mechanisms. Notable axes include: (i) drug efflux (ABCB1/ABCG2) → reduced intracellular drug levels; (ii) endocytosis/trafficking (AP2M1) → receptor signaling and nutrient uptake; (iii) immune modulation (TIM4) → efferocytosis and immune tolerance; (iv) metabolism/oxidative balance (PPARs, LXR-α, FABP1, HO-1) → growth and stress adaptation; (v) MAPK signaling → proliferation/migration; and (vi) cell death control (BAX↑, BCL2↑) → apoptosis set-point shifts. These roles provide mechanistic context for potential miRNA interventions (Table 2).

### 3.3 Impact of MPs on anticancer therapies

Reports indicate both sensitization and resistance, but the weight of evidence suggests attenuation of therapy efficacy in several contexts (e.g., altered uptake/efflux; MAPK-mediated survival; inflammation-mediated radioprotection). The table summarizes polymer/tumor-specific patterns and implicated agents (e.g., doxorubicin, taxanes, HER2/EGFR inhibitors), emphasizing the need to measure MP exposure in preclinical efficacy studies (Table 3).

### 3.4 miRNAs as therapeutic candidates

We collated anticancer miRNAs with functional evidence (apoptosis, invasion, chemosensitization). This pool served as input to the *in silico* screen. Where multiple candidates mapped to one target, prioritization was by relative mfe plus prior functional plausibility. Examples of anticancer miRNAs selected for screening are illustrated in Figure 3 (Supplementary Table S1).

### 3.5 Predicted miRNA–gene interactions

For breast cancer, miR-483-3p and miR-138-5p showed favorable predictions against ABCB1/PTP4A2 and TMBIM6, respectively, while miR-365 and miR-331-3p mapped to ABCG2/AP2M1. In gastric cancer, miR-665 and miR-760 targeted CD44 and ASGR2; in liver cancer, miR-638 showed broad predictions (e.g., HO-1, PPARα/γ). Lung cancer candidates clustered on MAPK nodes (miR-532-3p, miR-593-5p, miR-29b). These pairings nominate tractable validation sets (reporter assays ± MP exposure, phenotypic readouts). Representative RNAhybrid pairing diagrams and 3D cartoons for top-ranked pairs appear in Figure 3, and the integrated miRNA–gene–pathway network is provided in Figure 3 (Table 4).

**TABLE 4:** Detailed information about the binding affinity of microRNAs and genes in cancer affected by MPs.

Type of cancer affected by MPs	The kind of gene affected by MPs in this cancer	MicroRNA with therapeutic *in silico* effect on the gene affected by MPs		Sequence of microRNA
		The most proper therapeutic microRNA based on *in silico* data	Binding affinity	
Breast cancer	ABCB1	miR-483-3p	−28.2	UCACUCCUCUCCUCCCGUCUU
	ABCG2	miR-365	−31.8	AGGGACUUUUGGGGGCAGAUGUG
	AP2M1	miR-331-3p	−28.7	GCCCCUGGGCCUAUCCUAGAA
	FTH1	miR-3613-3p	−16.7	ACAAAAAAAAAAGCCCAACCCUUC
	PTP4A2	miR-483-3p	−31.9	UCACUCCUCUCCUCCCGUCUU
	TMBIM6	miR-138-5p	−34.4	AGCUGGUGUUGUGAAUCAGGCCG
Cervical cancer	TIM4	miR-302c-3p	−25.1	UAAGUGCUUCCAUGUUUCAGUGG
Colorectal cancer	MUC2	miR-144	−13.8	GGAUAUCAUCAUAUACUGUAAG
	TIM4	miR-296	−31.9	AGGGCCCCCCCUCAAUCCUGU
Gastric cancer	ASGR2	miR-760	−32	CGGCUCUGGGUCUGUGGGGA
	Bax	miR-1-3p	−14.1	UGGAAUGUAAAGAAGUAUGUAU
	CD44	miR-665	−35.5	ACCAGGAGGCUGAGGCCCCU
	TIM4	miR-490-3p	−27.9	CAACCUGGAGGACUCCAUGCUG
Leukemia	ABCB1	miR-370-3p	−24.7	GCCUGCUGGGGUGGAACCUGGU
	ABCG2	miR-520a	−26.6	CUCCAGAGGGAAGUACUUUCU
Liver cancer	FABP1	miR-638	−20	AGGGAUCGCGGGCGGGUGGCGGCCU
	HO-1	miR-638	−37.4	AGGGAUCGCGGGCGGGUGGCGGCCU
		miR-4270-5p	−37.4	UCAGGGAGUCAGGGGAGGGC
	LXR-α	miR-559	−15.7	UAAAGUAAAUAUGCACCAAAA
	PPARα	miR-638	−39.3	AGGGAUCGCGGGCGGGUGGCGGCCU
	PPARγ	miR-638	−29.2	AGGGAUCGCGGGCGGGUGGCGGCCU
Lung cancer	BRAF	miR-532-3p	−31	CCUCCCACACCCAAGGCUUGCA
		miR-593-5p	−31	AGGCACCAGCCAGGCAUUGCUCAGC
	c-jun	miR-92b	−33.8	AGGGACGGGACGCGGUGCAGUG
	ERK	miR-593-5p	−34.9	AGGCACCAGCCAGGCAUUGCUCAGC
	JNK	miR-532-3p	−34.9	CCUCCCACACCCAAGGCUUGCA
	MAP2K1	miR-593-5p	−33.9	AGGCACCAGCCAGGCAUUGCUCAGC
	MAP2K4	miR-611	−36.2	GCGAGGACCCCUCGGGGUCUGAC
	MAPK14	miR-377	−30.7	AGAGGUUGCCCUUGGUGAAUUC
	KRAS	miR-29b	−30.8	GCUGGUUUCAUAUGGUGGUUUAGA
	NLRP3	miR-92b	−33.1	AGGGACGGGACGCGGUGCAGUG
	BCL2	miR-593-5p	−37.7	AGGCACCAGCCAGGCAUUGCUCAGC
	TIM4	miR-425-5p	−25.7	AAUGACACGAUCACUCCCGUUGA
Skin cancer	NLRP3	miR-365	−28.9	AGGGACUUUUGGGGGCAGAUGUG
Pancreatic cancer	TIM4	miR-612	−25.4	GCUGGGCAGGGCUUCUGAGCUCCUU

Lower (more negative) mfe = stronger binding. Strong (≤−20), Suggestive (−15 to −19.9), Borderline (>−15). Borderline values are shown for completeness but require experimental validation.

Most high-affinity interactions showed mfe ≤ −20 kcal mol^−1^ (e.g., TMBIM6/miR-138-5p, CD44/miR-665, PPARα/miR-638, MAP2K4/miR-611). Moderate (−15 to −19.9) values such as FTH1/miR-3613-3p were retained due to prior experimental evidence of anticancer function. Borderline pairs (e.g., BAX/miR-1-3p, −14.1) were listed for transparency but are considered exploratory. Representative hybridization structures are illustrated in Figure 3.

## 4 Discussion

Our results are consistent with prior evidence that MP exposure modulates canonical cancer pathways. Enhanced efflux and drug resistance observed experimentally (Rosellini et al., 2023) parallel our identification of ABCB1/ABCG2 as MP-responsive genes and their suppression by miR-483-3p and miR-365. Polypropylene-induced metastasis (Park et al., 2023) corresponds to increased AP2M1/PTP4A2/TMBIM6, predicted to be inhibited by miR-331-3p and miR-138-5p. Upregulation of ASGR2 in gastric cancer (Kim et al., 2022) matches our predicted targeting by miR-760. Similarly, activation of MAPK and BCL2 signaling (Jain et al., 2021; Ramkumar et al., 2023; Zhou Z. et al., 2020) is reflected in our miR-532-3p/miR-593-5p predictions. These consistencies strengthen the biological plausibility of our computational findings while emphasizing the need for experimental validation.

Besides, MPs affect some genes in cancer cells and suppress their effects. For instance, Polymethyl methacrylate (PMMA) inhibits liver cancer cells by downregulating FABP1 and PPAR alpha. According to prior surveys, MPs can downregulate specific genes in tumor cells, such as BCAS3, PHF19, and PRKCD, whose expression has been suppressed by microplastics in previous studies (Chen et al., 2025).

Moreover, our study demonstrates that MPs can affect some genes in breast cancer. ABCB1 is one of these genes that encodes P-glycoprotein (P-gp), an ATP-binding cassette (ABC) efflux transporter involved in multidrug resistance (MDR) in breast cancer, which expels chemotherapeutic drugs from cells, reduces drug efficacy, and contributes to treatment failure (Miao et al., 2017). It regulates ion channels, affecting apoptosis, proliferation, and other cancer-related processes (Altamura et al., 2022).

ABCG2 is another gene affected by MPs, and it encodes Breast Cancer Resistance Protein (BCRP), another ABC transporter involved in MDR by exporting chemotherapeutic agents, such as mitoxantrone and doxorubicin, out of cancer cells, reducing drug efficacy (Zhang et al., 2022). It also influences drug bioavailability and toxicity, acting as a barrier in the blood-brain barrier, liver, and intestines, with alterations linked to treatment failure in cancers with high ABCG2 expression (Wang et al., 2020; Franczyk et al., 2022).

AP2M1, a key component of the clathrin adaptor protein complex, facilitates receptor-mediated endocytosis in cancer cells, enhancing nutrient and growth factor uptake, which supports tumor growth and survival (Shin et al., 2021; Liu et al., 2021; Münz, 2020). Its overexpression is associated with aggressive cancer phenotypes and chemoresistance (Liu et al., 2023). Notably, this gene is also influenced by MPs in breast cancer.

Other genes affected by MPs in breast cancer include FTH1, PTP4A2, and TMBIM6. FTH1, a tumor suppressor, regulates iron storage and oxidative stress protection in cancer cells and stabilizes p53 under stress conditions. Silencing FTH1 leads to increased tumor growth, migration, and chemoresistance, along with upregulation of oncogenes like c-MYC and G9a (Di Sanzo et al., 2020; Ali et al., 2021). PTP4A2, upregulated in breast cancer, promotes cancer progression through various pathways (Chouleur et al., 2024). TMBIM6, a key regulator of stress responses, is linked to increased chemoresistance, cancer progression, and metastasis in breast cancer, as well as reduced patient survivalTMBIM6, a key regulator of stress responses, is linked to increased chemoresistance, cancer progression, and metastasis in breast cancer, as well as reduced patient survival (Robinson et al., 2025).

The other effect on MPs on genes in cancer cells is their effect on cervical cancer cells. Based on prior surveys, TIM4, along with TIM3, plays an essential role in the degradation of dying tumor cells via autophagy, reducing antigen presentation and impairing cytotoxic T lymphocyte (CTL) responses, creating immune tolerance, and weakening the antitumor immune response (Junjappa et al., 2019).

Furthermore, the present study demonstrates that two genes in colorectal cancer (CRC) cells are affected by MPs: MUC2 and TIM4. MUC2 is a protective barrier in epithelial cells, playing a role in cell differentiation and maintaining the balance of adhesion. Altered MUC2 expression impacts the progression of CRC by influencing cellular proliferation, apoptosis, and epithelial integrity (Iranmanesh et al., 2021). Notably, the silencing of MUC2 increases IL-6 secretion, which activates the STAT3 signaling pathway, promoting tumor cell migration, epithelial-mesenchymal transition (EMT), and metastasis. MUC2 downregulation leads to the activation of STAT3 and Chk2, suppression of CREB phosphorylation, and loss of E-cadherin, facilitating cancer progression and metastasis (Hsu et al., 2017). Moreover, TIM4 acts as an oncogene through different mechanisms, including supporting tumor cell proliferation, migration, invasion, and immune evasion, and contributing to tumor immune tolerance by impairing antigen presentation and cytotoxic T cell responses (Liu et al., 2020).

According to our findings, ASGR2, Bax, CD44, and TIM4 are genes impacted by MPs in gastric cancer cells. ASGR2 enhances tumor survival and metastasis, with higher levels linked to poor prognosis in gastric cancer (Xue et al., 2021). CD44 regulates cell adhesion, motility, and survival, promoting gastric cancer progression through tumor growth, invasion, and metastasis (Jang et al., 2011). It also supports tumor survival by enhancing antioxidant defenses and reducing oxidative stress (Zavros, 2017). TIM4 is overexpressed in gastric cancer tissues, correlating with increased angiogenesis, tumor growth, and poorer patient survival outcomes (Wang et al., 2021). In contrast, Bax induces apoptosis in gastric cancer cells through the mitochondrial pathway, promoting pro-apoptotic signaling, mitochondrial membrane collapse, and subsequent caspase activation (Shabani et al., 2020; Shen et al., 2023).

The other genes affected by MPs in cancer cells are ABCB1 and ABCG2 in Leukemia. ABCB1 is an efflux transporter that helps pump chemotherapy drugs out of cells, and its activation contributes to MDR. In AML, overexpression of ABCB1 has been linked to poor treatment outcomes ABCB1 (Sucha et al., 2022). ABCG2 functions as an efflux transporter that can extrude a wide variety of chemotherapy drugs out of the cells, thereby reducing their effectiveness and contributing to drug resistance in leukemia cells. Moreover, the overexpression of ABCG2 in leukemia cells is associated with poor clinical outcomes, including reduced complete remission rates and overall survival (Damiani and Tiribelli, 2023).

In addition, FABP1, HO-1, LXR-α, PPAR-alpha (PPARα), and PPARγ have been affected by MPs in liver cancer cells. FABP1 supports tumor progression by maintaining the M2 phenotype of tumor-associated macrophages (TAMs), which is associated with immune suppression and cancer progression. FABP1 deficiency in TAMs reduced tumor growth, invasion, and migration *in vitro*, highlighting its role in enhancing cancer cell proliferation and aggressiveness (Tang et al., 2023). HO-1 promotes cancer cell survival by suppressing pro-apoptotic pathways, regulating mitochondrial oxidative stress, activating the transcription of antioxidant and detoxifying genes, and enhancing the cell’s ability to counteract oxidative damage and resist apoptosis (Alharbi et al., 2022). LXR-α plays a crucial role in regulating lipid metabolism, inflammation, and immune responses in HCC. Its activation inhibits tumor cell proliferation by inducing cell cycle arrest and apoptosis, and reduces tumor invasiveness and migration (Han et al., 2023).

PPARα plays a significant role in the development and progression of liver cancer by controlling lipid metabolism, glucose regulation, and inflammation in the liver cells (Pan et al., 2024). PPARγ is a protective factor in liver cancer by some mechanisms, including inhibiting hepatic fibrosis progression and inflammation, suppressing tumor microenvironment remodeling, and promoting apoptosis and senescence in hepatocellular carcinoma (HCC) cells (Ishtiaq et al., 2022).

Additionally, genes involved in the MAPK signaling pathway include BRAF, c-Jun, ERK, JNK, MAP2K1, MAP2K4, MAPK14, and KRAS, which are genes affected by MPs in lung cancer cells. These genes are crucial for cell proliferation and survival, and their activation leads to the uncontrolled growth of cancer cells (Pradhan et al., 2019). Other genes, such as NLRP3, BCL2, and TIM4, are also affected by MPs in the mentioned cancer. The activation of NLRP3 creates chronic inflammation that promotes tumors by inducing DNA damage, enhancing angiogenesis, and suppressing apoptosis in cancer cells (Tang et al., 2020). The BCL2 gene contributes to the resistance of small cell lung cancer (SCLC) to Aurora kinase B (AURKB) inhibitors. It suppresses apoptosis and DNA damage caused by these inhibitors, allowing cells to avoid programmed cell death even under therapeutic stress (Ramkumar et al., 2023). TIM4 acts as an oncogene by supporting tumor cell proliferation, migration, invasion, and immune evasion, and also contributes to tumor immune tolerance by impairing antigen presentation and cytotoxic T cell responses (Liu et al., 2020).

Additionally, MPs affect NLRP3 in skin cancer cells. NLRP3 enhances inflammation, stimulates angiogenesis, and promotes the proliferation and migration of tumor cells (Ciazyn et al., 2020). Additionally, our study revealed that TIM4 is another gene in pancreatic cancer cells that MPs can influence. TIM4 is crucial in creating an immune-suppressive environment, enabling tumor cells to evade immune detection. It also supports tumor progression by reducing the effectiveness of immune cells, such as macrophages and T cells, in targeting cancer cells (Shi et al., 2021).

MPs may also influence the efficacy of cancer therapies. They can alter the metabolism and bioavailability of therapeutic drugs by interfering with their absorption and distribution in the body. This could lead to either reduced or enhanced drug activity, depending on the interactions between the MPs and the drugs (Deng et al., 2025). Interestingly, our study also shows that MPs can reduce and enhance the anti-cancer drug activity. However, according to the prior surveys and our study, MPs can deteriorate the impacts of anti-cancer drugs (Zhao et al., 2024) and exert this resistance against various anticancer agents.

The present study highlights the potential of certain microRNAs as candidate therapeutics in breast cancer models; however, these predictions are hypothesis-generating and require validation in experimental and clinical contexts (Zhao et al., 2019). Our *in silico* analysis highlights microRNAs targeting genes influenced by MPs in breast cancer cells, aligning with prior findings on their anti-breast cancer capabilities (Menbari et al., 2020). Specific microRNAs, including miR-483-3p, miR-365, miR-331-3p, and miR-138-5p, demonstrated strong binding affinity for genes such as ABCB1, ABCG2, AP2M1, PTP4A2, and TMBIM6, marking them as promising candidates for microRNA therapy. These microRNAs also target various molecular pathways, enhancing their anti-cancer effects (Liu et al., 2019; Zhao et al., 2020; Rasoolnezhad et al., 2021; Shen et al., 2020). In-silico analysis also revealed miR-3613-3p’s potential to target FTH1, a gene involved in iron homeostasis and oxidative stress regulation in cancer cells (Di Sanzo et al., 2020), making it a key therapeutic candidate.

**FIGURE 4:**
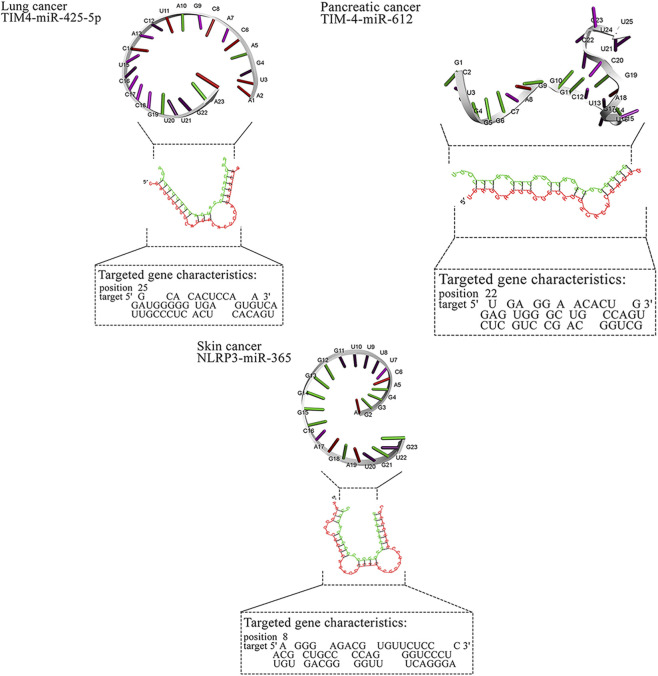
MicroRNAs with the *in silico* capability to suppress genes in MPs-based cancers and their possible targeted molecular pathways. The blue, red, green, and gray arrows represent activation (or upregulation), inhibition (or downregulation), regulation, and interaction, respectively. Moreover, yellow hexagons, blue ovals, and green ovals demonstrate microRNAs, targeted genes, and pathways affected by targeted genes, respectively. ABCB1, ATP Binding Cassette Subfamily B Member 1; ABCG2, ATP Binding Cassette Subfamily G Member 2; AP2M1, Adaptor Related Protein Complex 2 Subunit Mu 1; ASGR2, Asialoglycoprotein Receptor 2; Bax, BCL2 Associated X, Apoptosis Regulator; BCL2, B-cell CLL/lymphoma 2; BRAF, B-Raf Proto-Oncogene, Serine/Threonine Kinase; c-jun, Jun Proto-Oncogene, AP-1 Transcription Factor Subunit; CD44, CD44 Molecule (Indian Blood Group); ERK, Extracellular Signal-Regulated Kinase; FABP1, Fatty Acid Binding Protein 1; FTH1, Ferritin Heavy Chain 1; HO-1, Heme Oxygenase 1; JNK, c-Jun N-terminal Kinase; KRAS, KRAS Proto-Oncogene, GTPase; LXR-α, Liver X Receptor Alpha; MAP2K1, Mitogen-Activated Protein Kinase Kinase 1; MAP2K4, Mitogen-Activated Protein Kinase Kinase 4; MAPK14, Mitogen-Activated Protein Kinase 14; MUC2, Mucin 2; NLRP3, NLR Family Pyrin Domain Containing 3; NLRP3, NLR Family Pyrin Domain Containing 3; PPARα, Peroxisome Proliferator-Activated Receptor Alpha; PPARγ, Peroxisome Proliferator-Activated Receptor Gamma; PTP4A2, Protein Tyrosine Phosphatase Type IVA, Member 2; TMBIM6, Transmembrane BAX Inhibitor Motif Containing 6; TIM4, T-cell Immunoglobulin and Mucin-domain Containing-4; TIM4, T-cell Immunoglobulin and Mucin-domain Containing-4; TIM4, T-cell Immunoglobulin and Mucin-domain Containing-4; TIM4, T-cell Immunoglobulin and Mucin-domain Containing-4; TIM4, T-cell Immunoglobulin and Mucin-domain Containing-4.

In addition to breast cancer, microRNAs also exhibit anti-cancer effects in cervical cancer (Hasanzadeh et al., 2019; Ding et al., 2021), colorectal cancer (Cheng et al., 2020), gastric cancer (Spitz and Gavathiotis, 2022), leukemia (Gil-Kulik et al., 2024; Xiao et al., 2024), liver cancer (K et al., 2020; Ramalingam et al., 2024), lung cancer (Pradhan et al., 2019; Naeli et al., 2020), pancreatic cancer (Li et al., 2021; Javadrashid et al., 2021), and skin cancer (Mohamm et al., 2021). Our study corroborates these findings, with *in silico* analyses revealing specific microRNAs that interact with key genes associated with tumorigenesis in these cancers. For instance, miR-302c-3p targets TIM4 in cervical cancer, miR-144 targets MUC2 in colorectal cancer, miR-706 and miR-665 target ASGR2 and CD44 in gastric cancer, and miR-532-3p and miR-593-5p target MAPK genes in lung cancer. These microRNAs demonstrate their therapeutic potential by influencing various molecular pathways, as shown in Figure 4. Overall, the study reinforces the growing potential of microRNAs as targeted therapies across multiple malignancies.

### 4.1 Limitations

First, studies linking MPs to gene changes are heterogeneous in polymer type, size, dose, and exposure model; many use surrogates (e.g., plastic-related compounds) rather than standardized particles. Second, mfe predictions do not demonstrate binding or regulation; off-targeting and RNA context effects are likely. Third, we did not perform a quantitative meta-analysis due to heterogeneity and incomplete reporting.

### 4.2 Future work

We propose a tiered pipeline: 1. verify MP-induced gene changes under standardized exposures; 2. validate miRNA targeting (luciferase wild-type/mutant, protein knockdown, rescue); 3. evaluate phenotypes (viability, invasion, efflux, radiosensitization) with and without MPs; 4. test delivery and safety *in vivo*.

## 5 Conclusion

MP exposure has been reported to perturb cancer-relevant genes and therapy responses across tumor types. Our *in silico* analyses nominate miRNAs that may counter these MP-associated programs. These findings are hypothesis-generating and require rigorous experimental and translational validation.

## References

[B1] Alam S. Mohammad T. Padder R. A. Hassan M. I. Husain M. (2022a). Thymoquinone and quercetin induce enhanced apoptosis in non-small cell lung cancer in combination through the Bax/Bcl2 cascade. J. Cell Biochem. 123 (2), 259–274. 10.1002/jcb.30162 34636440

[B2] Alam M. Alam S. Shamsi A. Adnan M. Elasbali A. M. Al-Soud W. A. (2022b). Bax/Bcl-2 Cascade is regulated by the EGFR pathway: therapeutic targeting of non-small cell lung cancer. Front. Oncol. 12, 869672. 10.3389/fonc.2022.869672 35402265 PMC8990771

[B3] Alharbi K. S. Almalki W. H. Albratty M. Meraya A. M. Najmi A. Vyas G. (2022). The therapeutic role of nutraceuticals targeting the Nrf2/HO-1 signaling pathway in liver cancer. J. Food Biochem. 46 (10), e14357. 10.1111/jfbc.14357 35945911

[B4] Ali A. Shafarin J. Abu Jabal R. Aljabi N. Hamad M. Sualeh M. J. (2021). Ferritin heavy chain (FTH1) exerts significant antigrowth effects in breast cancer cells by inhibiting the expression of c-MYC. FEBS Open Bio 11 (11), 3101–3114. 10.1002/2211-5463.13303 34551213 PMC8564339

[B5] Altamura C. Gavazzo P. Pusch M. Desaphy J. F. (2022). Ion channel involvement in tumor drug resistance. J. Pers. Med. 12 (2), 210. 10.3390/jpm12020210 35207698 PMC8878471

[B6] Astuti Y. Raymant M. Quaranta V. Clarke K. Abudula M. Smith O. (2024). Efferocytosis reprograms the tumor microenvironment to promote pancreatic cancer liver metastasis. Nat. Cancer 5 (5), 774–790. 10.1038/s43018-024-00731-2 38355776 PMC11136665

[B7] Barani M. (2024). The role of environmental education in improving human health: literature review. West Kazakhstan Med. J. 66 (4), 373–386. 10.18502/wkmj.v66i4.17769

[B8] Boran T. Zengin O. S. Seker Z. Akyildiz A. G. Kara M. Oztas E. (2024). An evaluation of a hepatotoxicity risk induced by the microplastic polymethyl methacrylate (PMMA) using HepG2/THP-1 co-culture model. Environ. Sci. Pollut. Res. Int. 31 (20), 28890–28904. 10.1007/s11356-024-33086-3 38564126 PMC11058773

[B9] Brynzak-Schreiber E. Schogl E. Bapp C. Cseh K. Kopatz V. Jakupec M. A. (2024). Microplastics role in cell migration and distribution during cancer cell division. Chemosphere 353, 141463. 10.1016/j.chemosphere.2024.141463 38423146

[B10] Caronni N. Piperno G. M. Simoncello F. Romano O. Vodret S. Yanagihashi Y. (2021). TIM4 expression by dendritic cells mediates uptake of tumor-associated antigens and anti-tumor responses. Nat. Commun. 12 (1), 2237. 10.1038/s41467-021-22535-z 33854047 PMC8046802

[B11] Chen Y. Zeng Q. Luo Y. Song M. He X. Sheng H. (2024a). Polystyrene microplastics aggravate radiation-induced intestinal injury in mice. Ecotoxicol. Environ. Saf. 283, 116834. 10.1016/j.ecoenv.2024.116834 39106569

[B12] Chen T. Lin Q. Gong C. Zhao H. Peng R. (2024b). Research progress on micro (nano)Plastics exposure-induced miRNA-Mediated biotoxicity. Toxics 12 (7), 475. 10.3390/toxics12070475 39058127 PMC11280978

[B13] Chen Y. Zhang Z. Ji K. Zhang Q. Qian L. Yang C. (2025). Role of microplastics in the tumor microenvironment (review). Oncol. Lett. 29 (4), 193. 10.3892/ol.2025.14939 40041410 PMC11877014

[B14] Cheng B. Zhang Y. Wu Z. W. Cui Z. C. Li W. L. (2020). MiR-144 inhibits colorectal cancer cell migration and invasion by regulating PBX3. Eur. Rev. Med. Pharmacol. Sci. 24 (18), 9361–9369. 10.26355/eurrev_202009_23019 33015777

[B15] Chouleur T. Emanuelli A. Souleyreau W. Derieppe M. A. Leboucq T. Hardy S. (2024). PTP4A2 promotes glioblastoma progression and macrophage polarization under microenvironmental pressure. Cancer Res. Commun. 4 (7), 1702–1714. 10.1158/2767-9764.CRC-23-0334 38904264 PMC11238266

[B16] Ciazynska M. Bednarski I. A. Wodz K. Narbutt J. Lesiak A. (2020). NLRP1 and NLRP3 inflammasomes as a new approach to skin carcinogenesis. Oncol. Lett. 19 (3), 1649–1656. 10.3892/ol.2020.11284 32194656 PMC7039172

[B17] Damiani D. Tiribelli M. (2023). ABCG2 in acute myeloid leukemia: old and new perspectives. Int. J. Mol. Sci. 24 (8), 7147. 10.3390/ijms24087147 37108308 PMC10138346

[B18] Deng X. Gui Y. Zhao L. (2025). The micro(nano)plastics perspective: exploring cancer development and therapy. Mol. Cancer 24 (1), 30. 10.1186/s12943-025-02230-z 39856719 PMC11761189

[B19] Di Sanzo M. Quaresima B. Biamonte F. Palmieri C. Faniello M. C. (2020). FTH1 pseudogenes in cancer and cell metabolism. Cells 9 (12), 2554. 10.3390/cells9122554 33260500 PMC7760355

[B20] Ding H. M. Zhang H. Wang J. Zhou J. H. Shen F. R. Ji R. N. (2021). miR-302c-3p and miR-520a-3p suppress the proliferation of cervical carcinoma cells by targeting CXCL8. Mol. Med. Rep. 23 (5), 322–10. 10.3892/mmr.2021.11961 33760117 PMC7974325

[B21] Franczyk B. Rysz J. Gluba-Brzozka A. (2022). Pharmacogenetics of drugs used in the treatment of cancers. Genes (Basel) 13 (2), 311. 10.3390/genes13020311 35205356 PMC8871547

[B22] Gil-Kulik P. Kluz N. Przywara D. Petniak A. Wasilewska M. Fraczek-Chudzik N. (2024). Potential use of exosomal non-coding MicroRNAs in leukemia therapy: a systematic review. Cancers (Basel) 16 (23), 3948. 10.3390/cancers16233948 39682135 PMC11639955

[B23] Hale R. C. Seeley M. E. La Guardia M. J. Mai L. Zeng E. Y. (2020). A global perspective on microplastics. J. Geophys. Research-Oceans 125 (1), e2018JC014719. 10.1029/2018JC014719

[B24] Han N. Yuan M. Yan L. Tang H. (2023). Emerging insights into liver X receptor alpha in the tumorigenesis and therapeutics of human cancers. Biomolecules 13 (8), 1184. 10.3390/biom13081184 37627249 PMC10452869

[B25] Hasanzadeh M. Movahedi M. Rejali M. Maleki F. Moetamani-Ahmadi M. Seifi S. (2019). The potential prognostic and therapeutic application of tissue and circulating microRNAs in cervical cancer. J. Cell Physiol. 234 (2), 1289–1294. 10.1002/jcp.27160 30191988

[B26] Hsu H. P. Lai M. D. Lee J. C. Yen M. C. Weng T. Y. Chen W. C. (2017). Mucin 2 silencing promotes colon cancer metastasis through interleukin-6 signaling. Sci. Rep. 7 (1), 5823. 10.1038/s41598-017-04952-7 28725043 PMC5517441

[B27] Hu X. Yu Q. Gatheru Waigi M. Ling W. Qin C. Wang J. (2022). Microplastics-sorbed phenanthrene and its derivatives are highly bioaccessible and may induce human cancer risks. Environ. Int. 168, 107459. 10.1016/j.envint.2022.107459 35964535

[B28] Iranmanesh H. Majd A. Mojarad E. N. Zali M. R. Hashemi M. (2021). Investigating the relationship between the expression level of mucin gene cluster (MUC2, MUC5A, and MUC5B) and clinicopathological characterization of colorectal cancer. Galen. Med. J. 10, e2030. 10.31661/gmj.v10i0.2030 35572847 PMC9086863

[B29] Ishtiaq S. M. Arshad M. I. Khan J. A. (2022). PPARγ signaling in hepatocarcinogenesis: mechanistic insights for cellular reprogramming and therapeutic implications. Pharmacol. Ther. 240, 108298. 10.1016/j.pharmthera.2022.108298 36243148

[B30] Jain A. S. Prasad A. Pradeep S. Dharmashekar C. Achar R. R. Ekaterina S. (2021). Everything old is new again: drug repurposing approach for non-small cell lung cancer targeting MAPK signaling pathway. Front. Oncol. 11, 741326. 10.3389/fonc.2021.741326 34692523 PMC8526962

[B31] Jang B. I. Li Y. Graham D. Y. Cen P. (2011). The role of CD44 in the pathogenesis, diagnosis, and therapy of gastric cancer. Gut Liver 5 (4), 397–405. 10.5009/gnl.2011.5.4.397 22195236 PMC3240781

[B32] Javadrashid D. Mohammadzadeh R. Baghbanzadeh A. Safaee S. Amini M. Lotfi Z. (2021). Simultaneous microRNA-612 restoration and 5-FU treatment inhibit the growth and migration of human PANC-1 pancreatic cancer cells. EXCLI J. 20, 160–173. 10.17179/excli2020-2900 33564285 PMC7868639

[B33] Jia S. Yang Y. Zhu Y. Yang W. Ling L. Wei Y. (2024). Association of FTH1-Expressing circulating tumor cells with efficacy of neoadjuvant chemotherapy for patients with breast cancer: a prospective cohort study. Oncologist 29 (1), e25–e37. 10.1093/oncolo/oyad195 37390841 PMC10769790

[B34] Junjappa R. P. Kim H. K. Park S. Y. Bhattarai K. R. Kim K. W. Soh J. W. (2019). Expression of TMBIM6 in cancers: the involvement of Sp1 and PKC. Cancers (Basel) 11 (7), 974. 10.3390/cancers11070974 31336725 PMC6678130

[B35] Karbasforooshan H. Hayes A. W. Mohammadzadeh N. Zirak M. R. Karimi G. (2020). The possible role of sirtuins and microRNAs in hepatocellular carcinoma therapy. Cell Cycle 19 (23), 3209–3221. 10.1080/15384101.2020.1843813 33164623 PMC7751631

[B36] K C. P. Maharjan A. Acharya M. Lee D. Kusma S. Gautam R. (2023). Polytetrafluorethylene microplastic particles mediated oxidative stress, inflammation, and intracellular signaling pathway alteration in human derived cell lines. Sci. Total Environ. 897, 165295. 10.1016/j.scitotenv.2023.165295 37419366

[B37] Kaliyev A. A. Mussin N. M. Tamadon A. (2024). The importance of mesenchymal stromal/stem cell therapy for cancer. West Kazakhstan Med. J., 106–110. 10.18502/wkmj.v66i2.16452

[B38] Kim H. Zaheer J. Choi E. J. Kim J. S. (2022). Enhanced ASGR2 by microplastic exposure leads to resistance to therapy in gastric cancer. Theranostics 12 (7), 3217–3236. 10.7150/thno.73226 35547772 PMC9065185

[B39] Kumar N. Lamba M. Pachar A. K. Yadav S. Acharya A. (2024). Microplastics - a growing concern as carcinogens in cancer etiology: emphasis on biochemical and molecular mechanisms. Cell Biochem. Biophysics 82 (4), 3109–3121. 10.1007/s12013-024-01436-0 39031249

[B40] Li X. Jiang W. Gan Y. Zhou W. (2021). The application of exosomal MicroRNAs in the treatment of pancreatic cancer and its research progress. Pancreas 50 (1), 12–16. 10.1097/MPA.0000000000001713 33370018

[B41] Li S. Keenan J. I. Shaw I. C. Frizelle F. A. (2023). Could microplastics be a driver for early onset colorectal cancer? Cancers (Basel) 15 (13), 3323. 10.3390/cancers15133323 37444433 PMC10340669

[B42] Liu F. Zhuang L. Wu R. X. Li D. Y. (2019). miR-365 inhibits cell invasion and migration of triple negative breast cancer through ADAM10. J. Buon 24 (5), 1905–1912. 31786854

[B43] Liu W. Xu L. Liang X. Liu X. Zhao Y. Ma C. (2020). Tim-4 in health and disease: friend or foe? Front. Immunol. 11, 537. 10.3389/fimmu.2020.00537 32300343 PMC7142236

[B44] Liu Q. Bautista-Gomez J. Higgins D. A. Yu J. Xiong Y. (2021). Dysregulation of the AP2M1 phosphorylation cycle by LRRK2 impairs endocytosis and leads to dopaminergic neurodegeneration. Sci. Signal 14 (693), eabg3555. 10.1126/scisignal.abg3555 34315807 PMC8486338

[B45] Liu X. Zhao X. Yang J. Wang H. Piao Y. Wang L. (2023). High expression of AP2M1 correlates with worse prognosis by regulating immune microenvironment and drug resistance to R-CHOP in diffuse large B cell lymphoma. Eur. J. Haematol. 110 (2), 198–208. 10.1111/ejh.13895 36335584

[B46] Menbari M. N. Rahimi K. Ahmadi A. Mohammadi-Yeganeh S. Elyasi A. Darvishi N. (2020). miR-483-3p suppresses the proliferation and progression of human triple negative breast cancer cells by targeting the HDAC8>oncogene. J. Cell Physiol. 235 (3), 2631–2642. 10.1002/jcp.29167 31508813

[B47] Miao L. Guo S. Lin C. M. Liu Q. Huang L. (2017). Nanoformulations for combination or cascade anticancer therapy. Adv. Drug Deliv. Rev. 115, 3–22. 10.1016/j.addr.2017.06.003 28624477 PMC5591780

[B48] Mohammadi M. Spotin A. Mahami-Oskouei M. Shanehbandi D. Ahmadpour E. Casulli A. (2021). MicroRNA-365 promotes apoptosis in human melanoma cell A375 treated with hydatid cyst fluid of Echinococcus granulosus sensu stricto. Microb. Pathog. 153, 104804. 10.1016/j.micpath.2021.104804 33609644

[B49] Münz C. (2020). Autophagy proteins influence endocytosis for MHC restricted antigen presentation (Elsevier). 10.1016/j.semcancer.2019.03.005 30928540

[B50] Naeli P. Yousefi F. Ghasemi Y. Savardashtaki A. Mirzaei H. (2020). The role of MicroRNAs in lung cancer: implications for diagnosis and therapy. Curr. Mol. Med. 20 (2), 90–101. 10.2174/1566524019666191001113511 31573883

[B51] Pan Y. Li Y. Fan H. Cui H. Chen Z. Wang Y. (2024). Roles of the peroxisome proliferator-activated receptors (PPARs) in the pathogenesis of hepatocellular carcinoma (HCC). Biomed. Pharmacother. 177, 117089. 10.1016/j.biopha.2024.117089 38972148

[B52] Park J. H. Hong S. Kim O. H. Kim C. H. Kim J. Kim J. W. (2023). Polypropylene microplastics promote metastatic features in human breast cancer. Sci. Rep. 13 (1), 6252. 10.1038/s41598-023-33393-8 37069244 PMC10108816

[B53] Pradhan R. Singhvi G. Dubey S. K. Gupta G. Dua K. (2019). MAPK pathway: a potential target for the treatment of non-small-cell lung carcinoma. Future Med. Chem. 11 (8), 793–795. 10.4155/fmc-2018-0468 30994024

[B54] Ramalingam P. S. Elangovan S. Mekala J. R. Arumugam S. (2024). Liver X receptors (LXRs) in cancer-an Eagle's view on molecular insights and therapeutic opportunities. Front. Cell Dev. Biol. 12, 1386102. 10.3389/fcell.2024.1386102 38550382 PMC10972936

[B55] Ramkumar K. Tanimoto A. Della Corte C. M. Stewart C. A. Wang Q. Shen L. (2023). Targeting BCL2 overcomes resistance and augments response to Aurora kinase B inhibition by AZD2811 in small cell lung cancer. Clin. Cancer Res. 29 (16), 3237–3249. 10.1158/1078-0432.CCR-23-0375 37289191 PMC10527398

[B56] Rasoolnezhad M. Safaralizadeh R. Hosseinpourfeizi M. A. Banan-Khojasteh S. M. Baradaran B. (2021). MiRNA-138-5p: a strong tumor suppressor targeting PD-L-1 inhibits proliferation and motility of breast cancer cells and induces apoptosis. Eur. J. Pharmacol. 896, 173933. 10.1016/j.ejphar.2021.173933 33545160

[B57] Robinson K. S. Sennhenn P. Yuan D. S. Liu H. Taddei D. Qian Y. (2025). TMBIM6/BI-1 is an intracellular environmental regulator that induces paraptosis in cancer *via* ROS and Calcium-activated ERAD II pathways. Oncogene 44 (8), 494–512. 10.1038/s41388-024-03222-x 39609612 PMC11832424

[B58] Rosellini M. Turunen P. Efferth T. (2023). Impact of plastic-related compounds on P-Glycoprotein and breast cancer resistance protein *in vitro* . Molecules 28 (6), 2710. 10.3390/molecules28062710 36985682 PMC10058098

[B59] Shabani F. Mahdavi M. Imani M. Hosseinpour-Feizi M. A. Gheibi N. (2020). Calprotectin (S100A8/S100A9)-induced cytotoxicity and apoptosis in human gastric cancer AGS cells: alteration in expression levels of Bax, Bcl-2, and ERK2. Hum. Exp. Toxicol. 39 (8), 1031–1045. 10.1177/0960327120909530 32167384

[B60] Shen S. Zhang S. Liu P. Wang J. Du H. (2020). Potential role of microRNAs in the treatment and diagnosis of cervical cancer. Cancer Genet. 248-249, 25–30. 10.1016/j.cancergen.2020.09.003 32987255

[B61] Shen J. Yang H. Qiao X. Chen Y. Zheng L. Lin J. (2023). The E3 ubiquitin ligase TRIM17 promotes gastric cancer survival and progression *via* controlling BAX stability and antagonizing apoptosis. Cell Death Differ. 30 (10), 2322–2335. 10.1038/s41418-023-01221-1 37697039 PMC10589321

[B62] Shi B. Chu J. Huang T. Wang X. Li Q. Gao Q. (2021). The scavenger receptor MARCO expressed by tumor-associated macrophages are highly associated with poor pancreatic cancer prognosis. Front. Oncol. 11, 771488. 10.3389/fonc.2021.771488 34778091 PMC8586414

[B63] Shin J. Nile A. Oh J. W. (2021). Role of adaptin protein complexes in intracellular trafficking and their impact on diseases. Bioengineered 12 (1), 8259–8278. 10.1080/21655979.2021.1982846 34565296 PMC8806629

[B64] Shin Y. Choi H. Y. Kwak Y. Yang G. M. Jeong Y. Jeon T. I. (2023). TMBIM6-mediated miR-181a expression regulates breast cancer cell migration and invasion *via* the MAPK/ERK signaling pathway. J. Cancer 14 (4), 554–572. 10.7150/jca.81600 37057283 PMC10088543

[B65] Spitz A. Z. Gavathiotis E. (2022). Physiological and pharmacological modulation of BAX. Trends Pharmacol. Sci. 43 (3), 206–220. 10.1016/j.tips.2021.11.001 34848097 PMC8840970

[B66] Sucha S. Sorf A. Svoren M. Vagiannis D. Ahmed F. Visek B. (2022). ABCB1 as a potential beneficial target of midostaurin in acute myeloid leukemia. Biomed. Pharmacother. 150, 112962. 10.1016/j.biopha.2022.112962 35462331

[B67] Sun L. Liu H. Ye Y. Lei Y. Islam R. Tan S. (2023). Smart nanoparticles for cancer therapy. Signal Transduct. Target Ther. 8 (1), 418. 10.1038/s41392-023-01642-x 37919282 PMC10622502

[B68] Szczepanek J. Skorupa M. Tretyn A. (2022). MicroRNA as a potential therapeutic molecule in cancer. Cells 11 (6), 1008. 10.3390/cells11061008 35326459 PMC8947269

[B69] Tang D. Liu H. Zhao Y. Qian D. Luo S. Patz E. F. Jr. (2020). Genetic variants of BIRC3 and NRG1 in the NLRP3 inflammasome pathway are associated with non-small cell lung cancer survival. Am. J. Cancer Res. 10 (8), 2582–2595. 32905523 PMC7471354

[B70] Tang W. Sun G. Ji G. W. Feng T. Zhang Q. Cao H. (2023). Single-cell RNA-sequencing atlas reveals an FABP1-dependent immunosuppressive environment in hepatocellular carcinoma. J. Immunother. Cancer 11 (11), e007030. 10.1136/jitc-2023-007030 38007237 PMC10679975

[B71] Vethaak A. D. Legler J. (2021). Microplastics and human health. Science. 371 (6530), 672–674. 10.1126/science.abe5041 33574197

[B72] Wang Y. Fang Z. Hong M. Yang D. Xie W. (2020). Long-noncoding RNAs (lncRNAs) in drug metabolism and disposition, implications in cancer chemo-resistance. Acta Pharm. Sin. B 10 (1), 105–112. 10.1016/j.apsb.2019.09.011 31993309 PMC6976993

[B73] Wang W. Min K. Chen G. Zhang H. Deng J. Lv M. (2021). Use of bioinformatic database analysis and specimen verification to identify novel biomarkers predicting gastric cancer metastasis. J. Cancer 12 (19), 5967–5976. 10.7150/jca.58768 34476011 PMC8408128

[B74] Wang Y. Xu X. Jiang G. (2023). Microplastics exposure promotes the proliferation of skin cancer cells but inhibits the growth of normal skin cells by regulating the inflammatory process. Ecotoxicol. Environ. Saf. 267, 115636. 10.1016/j.ecoenv.2023.115636 37918331

[B75] Winiarska E. Jutel M. Zemelka-Wiacek M. (2024). The potential impact of nano- and microplastics on human health: understanding human health risks. Environ. Res. 251 (Pt 2), 118535. 10.1016/j.envres.2024.118535 38460665

[B76] Xiao J. Wan F. Tian L. Li Y. (2024). Tumor suppressor miR-520a inhibits cell growth by negatively regulating PI3K/AKT signaling pathway in acute myeloid leukemia. Adv. Clin. Exp. Med. 33 (7), 729–738. 10.17219/acem/171299 37855060

[B77] Xue S. Ma M. Bei S. Li F. Wu C. Li H. (2021). Identification and validation of the immune regulator CXCR4 as a novel promising target for gastric cancer. Front. Immunol. 12, 702615. 10.3389/fimmu.2021.702615 34322132 PMC8311657

[B78] Yan X. Zhang Y. Lu Y. He L. Qu J. Zhou C. (2020). The complex toxicity of tetracycline with polystyrene spheres on gastric cancer cells. Int. J. Environ. Res. Public Health 17 (8), 2808. 10.3390/ijerph17082808 32325809 PMC7216245

[B79] Yu M. Lin C. Wei M. (2023). A pan-cancer analysis of oncogenic protein tyrosine phosphatase subfamily PTP4As. J. Holist. Integr. Pharm. 4 (2), 185–198. 10.1016/j.jhip.2023.07.001

[B80] Zavros Y. (2017). Initiation and maintenance of gastric cancer: a focus on CD44 variant isoforms and cancer stem cells. Cell Mol. Gastroenterol. Hepatol. 4 (1), 55–63. 10.1016/j.jcmgh.2017.03.003 28560289 PMC5439237

[B81] Zhang Y. S. Yang C. Han L. Liu L. Liu Y. J. (2022). Expression of BCRP/ABCG2 protein in invasive breast cancer and response to neoadjuvant chemotherapy. Oncol. Res. Treat. 45 (3), 94–101. 10.1159/000520871 34775385

[B82] Zhao J. Li D. Fang L. (2019). MiR-128-3p suppresses breast cancer cellular progression *via* targeting LIMK1. Biomed. Pharmacother. 115, 108947. 10.1016/j.biopha.2019.108947 31078043

[B83] Zhao M. Zhang M. Tao Z. Cao J. Wang L. Hu X. (2020). miR-331-3p suppresses cell proliferation in TNBC cells by downregulating NRP2. Technol. Cancer Res. Treat. 19, 1533033820905824. 10.1177/1533033820905824 32174262 PMC7076578

[B84] Zhao J. Zhang H. Shi L. Jia Y. Sheng H. (2024). Detection and quantification of microplastics in various types of human tumor tissues. Ecotoxicol. Environ. Saf. 283, 116818. 10.1016/j.ecoenv.2024.116818 39083862

[B85] Zhou Z. Zhou Q. Wu X. Xu S. Hu X. Tao X. (2020a). VCAM-1 secreted from cancer-associated fibroblasts enhances the growth and invasion of lung cancer cells through AKT and MAPK signaling. Cancer Lett. 473, 62–73. 10.1016/j.canlet.2019.12.039 31904479

[B86] Zhou Z. G. Xu C. Dong Z. Wang Y. P. Duan J. Y. Yan C. Q. (2020b). MiR-497 inhibits cell proliferation and invasion ability by targeting HMGA2 in pancreatic ductal adenocarcinoma. Eur. Rev. Med. Pharmacol. Sci. 24 (1), 122–129. 10.26355/eurrev_202001_19901 31957824

