# Supplementary material for: In-silico pharmacological insights into the therapeutic potential of microRNAs for microplastic-associated cancers
Source: Front Cell Dev Biol. 2025 Nov 25;13:1699693. doi: 10.3389/fcell.2025.1699693 (PMC12685920; doi:10.3389/fcell.2025.1699693)
Supplement: Supplementary file 1 [file Table1.docx]

# Supplementary tables

## Table S1. MicroRNAs in the treatment of MP-based cancers

| MicroRNA | Reference | MicroRNA | Reference | MicroRNA | Reference | MicroRNA | Reference | MicroRNA | Reference |
| --- | --- | --- | --- | --- | --- | --- | --- | --- | --- |
| Leukemia | | Breast cancer | | Liver cancer | | Colorectal cancer | | Lung cancer | |
| miR-29b-3p | (1) | miR-1 | (2) | miR-9 | (3) | miR-7 | (4) | miR-1-3p | (5) |
| miR-34a | (6, 7) | miR-16-5p | (8) | miR-16-5p | (9) | miR-9-5p | (10) | miR-16 | (11) |
| miR-34b | (12) | miR-26b | (13) | miR-27a-3p | (14) | miRNA-20b-5p | (15) | miR-23b | (16) |
| miR-96 | (17) | miR-96-5p | (18) | miR-29c-3p | (19) | miR-22-3p | (20) | miR-29b | (21) |
| miR-101 | (22) | miR-126-3p | (23) | miR-33b | (24) | miR-30b-5p | (25) | miR-30a-5p | (26) |
| miR-181a-5p | (27) | miR-128-3p | (28) | miR-34a-5p | (29) | miR-31 | (30) | miR-30e | (31) |
| miR-182 | (32) | miR-129-1-3p | (33) | miR-101-3p | (34) | miR-93-5p | (35) | miR-34b | (36) |
| miR-203 | (37) | miR-133 | (38) | miR-122 | (39) | miR-101 | (40) | miR-34b-3p | (41) |
| miR-211 | (42) | miR-138-5p | (43) | miR-125a-5p | (44) | miR-124 | (45) | miR-34c | (46) |
| miR-338-3p | (47) | miR-140-5p | (48) | miR-144-3p | (49) | miR-124-3p | (50) | miR-92b | (51) |
| miR-342-3p | (52) | miR-142-3p | (53) | miR-148a-3p | (54) | miR-125 | (55) | miR-125a | (56) |
| miR-342-5p | (57) | miR-143-5p | (58) | miR-152-3p | (59) | miR-129-5p | (60) | miR-129-2 | (61) |
| miR-361-3p | (62) | miR-152 | (63) | miR-155 | (64) | miR-130a-3p | (65) | miR-139-5p | (66) |
| miR-370-3p | (67) | miR-182-3p | (68) | miR-181a | (3) | miR-137-3p | (69) | miR-140-3p | (70) |
| miR-376a-3p | (71) | miR-184 | (72) | miR-199a | (73) | miR-141-3p | (74) | miR-142-3p | (75) |
| miR-381-3p | (76) | miR-186 | (77) | miR-200b | (78) | miR-142-3p | (79) | miR-144-3p | (80) |
| miR-454-3p | (81) | miR-188-5p | (82) | miR-297 | (83) | miR-144 | (84) | miR-146a-5p | (85) |
| miR-520a | (86) | miR‑192 | (87) | miR-302a | (88) | miR-192 | (89) | miR-148b | (90) |
| miR-582-3p | (91) | miR-192-5p | (92) | miR-329-3p | (93) | miR-193a | (94) | miR-181c-5p | (95) |
|  |  | miR-204 | (96) | miR-383 | (97) | miR-203a | (98) | miR-181d-5p | (99) |
|  |  | miR-216a | (100) | miR-339-5p | (101) | miR-205 | (102) | miR-183-5p | (103) |
|  |  | miR-216a-5p | (104) | miR-342-3p | (105) | miR-212 | (106) | miR-187 | (107) |
|  |  | miR-216b | (108) | miR-370 | (109) | miR-214 | (110) | miR-194 | (111) |
|  |  | miR-322 | (112) | miR-429 | (113) | miR-296 | (114) | miR-195-5p | (115) |
|  |  | miR-331-3p | (116) | miR-455-3p | (117) | miR-328-3p | (118) | miR-199a-3p | (119) |
|  |  | miR-342-3p | (120) | miR-485-5p | (121) | miR-335-5p | (122) | miR-200c-3p | (123) |
|  |  | miR-365 | (124) | miR-486-5p | (125) | miR-375-3p | (126) | miR-204 | (127) |
|  |  | miR-374c-5p | (128) | miR-490-5p | (129) | miR-378a-5p | (130) | miR-221 | (131) |
|  |  | miR-409 | (132) | miR-495 | (133) | miR-488 | (134) | miR-325 | (135) |
|  |  | miR-449a | (136) | miR-499 | (137) | miR-498 | (138) | miR-338-3p | (139) |
|  |  | miR-451a | (140) | miR-559 | (141) | miR-506-3p | (142) | miR-338-5p | (143) |
|  |  | miR-483-3p | (144) | miR-597-5p | (145) | miR-509-5p | (146) | miR-342-3p | (147) |
|  |  | miR-511 | (148) | miR-627-5p | (149) | miR-582-5p | (150) | miR-377 | (151) |
|  |  | miR-518a-5p | (152) | miR-638 | (153) | miR-876-3p | (154) | miR-379 | (155) |
|  |  | miR-519e-5p | (156) | miR-936 | (157) | miR-760 | (158) | miR-381 | (159) |
|  |  | miR-524-5p | (160) | miR-1914 | (161) | miR-766-3p | (162) | miRNA-425-5p | (163) |
|  |  | miR-526b | (164) | miR-2392 | (165) | miR-1266-3p | (166) | miR-486-5p | (167) |
|  |  | miR-539 | (168) | miR-4270-5p | (169) | miR-3622a-3p | (170) | miR-497 | (171) |
|  |  | miR-548c-5p | (172) | miR-4310 | (173) | miR-4319 | (174) | miR-512-5p | (175) |
|  |  | miR-641 | (176) | miR-4651 | (177) | miR-4429-5p | (178) | miR-520a | (179) |
|  |  | miR-1284 | (180) |  |  | miR-4461 | (181) | miR-532-3p | (182) |
|  |  | miR-1298 | (183) |  |  | miR-4746 | (184) | miR-577 | (185) |
|  |  | miR-3613-3p | (186) |  |  | miR-6511b-5p | (187) | miR-584-5p | (188) |
|  |  | miR-4458 | (189) |  |  |  |  | miR-590 | (190) |
|  |  |  |  |  |  |  |  | miR-593-5p | (191) |
|  |  |  |  |  |  |  |  | miR-611 | (192) |
|  |  |  |  |  |  |  |  | miR-655-3p | (193) |
|  |  |  |  |  |  |  |  | miR-1915-3p | (194) |
|  |  |  |  |  |  |  |  | miR-4732-3p | (195) |
| MicroRNA | Reference | MicroRNA | Reference | MicroRNA | Reference | MicroRNA | Reference | miR-654-3p | (196) |
| Gastric cancer | | Cervical cancer | | Skin cancer | | Pancreatic cancer | |  |  |
| miR-1-3p | (197) | miR-26b | (198) | miR-29c | (199) | miR-24-3p | (200) |  |  |
| miR-9-5p | (201) | miR-29a | (202) | miR-33a-5p | (203) | miR-26a | (204) |  |  |
| miR-19b-3p | (205) | miR-32-5p | (206) | miR-127 | (207) | miR-33a-3p | (208) |  |  |
| miR-24 | (209) | miR-34c-5p | (210) | miR-130a | (211) | miR-33a-5p | (212) |  |  |
| miR-29c | (213) | miR-100 | (214) | miR-135b-5p | (215) | miR-139 | (216) |  |  |
| miR-31 | (217) | miR-137 | (218) | miR-139-5p | (219) | miR‑142‑5p | (220) |  |  |
| miR-33a | (221) | miR-138 | (222) | miR-140-5p | (223) | miR-143-3p | (224) |  |  |
| miR-34a | (225) | miR-142-5p | (226) | miR-155 | (227) | miR-145 | (228) |  |  |
| miR-34c-5p | (229) | miR-145 | (230) | miR-193a-3p | (231) | miR-203 | (232) |  |  |
| miR-105 | (233) | miR-152 | (234) | miR-193a-5p |  | miR-211-5p | (235) |  |  |
| miR-107 | (236) | miR-186-3p | (237) | miR-200b-3p | (238) | miR-217 | (239) |  |  |
| miR-124-5p | (240) | miR-204 | (241) | miR-224-5p | (242) | miR-320b | (243) |  |  |
| miR-126 | (244) | miR-214-3p | (245) | miR-365 | (246) | miR-337 | (247) |  |  |
| miR-129-3p | (248) | miR-302c-3p | (249) | miR-429-5p | (250) | miR-345-5p | (251) |  |  |
| miR-129-5p | (252) | miR-326 | (253) | miR-524-5p | (254) | miR-374 | (255) |  |  |
| miR-140 | (256) | miR-362-3p | (257) | miR-590-5p | (258) | miR‑381 | (259) |  |  |
| miR-148a-3p | (260) | miR-374b | (261) | miR-767-3p | (262) | miR-382 | (263) |  |  |
| miR-149 | (264) | miR-377 | (265) | miR-876-3p | (266) | miR-455 | (267) |  |  |
| miR-198 | (268) | miR-520a-3p | (249) | miR-3619-5p | (269) | miR-497 | (270) |  |  |
| miR-204-3p | (271) | miR-526b | (272) | miR-4458 | (273) | miR-519 | (274) |  |  |
| miR-205-5p | (275) | miR-613 | (276) |  |  | miR-519d-3p | (277) |  |  |
| miR-206 | (278) | miR-636 | (279) |  |  | miR-539 | (280) |  |  |
| miR-216b | (281) | miR-638 | (282) |  |  | miR-543 | (283) |  |  |
| miR‑299‑3p | (284) | miR-664 | (285) |  |  | miR-573 | (286) |  |  |
| miR-345 | (287) | miR-802 | (288) |  |  | miR-597-5p | (289) |  |  |
| miR-361-5p | (290) | miR-1258 | (291) |  |  | miR-608 | (292) |  |  |
| miR-365 | (293) | miR-3184-5p | (294) |  |  | miR-612 | (295) |  |  |
| miR-381 | (296) | miR-3929 | (297) |  |  | miR-634 | (298) |  |  |
| miR-383-5p | (299) |  |  |  |  | miR-4269 | (300) |  |  |
| miR-411 | (301) |  |  |  |  |  |  |  |  |
| miR-431-5p | (302) |  |  |  |  |  |  |  |  |
| miR-455-3p | (303) |  |  |  |  |  |  |  |  |
| miR-490-3p | (304) |  |  |  |  |  |  |  |  |
| miR-491 | (305) |  |  |  |  |  |  |  |  |
| miR-520-3p | (306) |  |  |  |  |  |  |  |  |
| miR-582-3p | (307) |  |  |  |  |  |  |  |  |
| miR-596 | (308) |  |  |  |  |  |  |  |  |
| miR-621 | (309) |  |  |  |  |  |  |  |  |
| miR-643 | (310) |  |  |  |  |  |  |  |  |
| miR-665 | (311) |  |  |  |  |  |  |  |  |
| miR-711 | (312) |  |  |  |  |  |  |  |  |
| miR-760 | (313) |  |  |  |  |  |  |  |  |
| miR-874-3p | (314) |  |  |  |  |  |  |  |  |
| miR-936 | (315) |  |  |  |  |  |  |  |  |
| miR-1179 | (316) |  |  |  |  |  |  |  |  |
| miR-1915 | (317) |  |  |  |  |  |  |  |  |
| miR-1275 | (318) |  |  |  |  |  |  |  |  |
| miR-1286 | (319) |  |  |  |  |  |  |  |  |
| miR-1324 | (320) |  |  |  |  |  |  |  |  |
| miR-4268 | (321) |  |  |  |  |  |  |  |  |
| miR-4429 | (322) |  |  |  |  |  |  |  |  |
| miR-5683 | (323) |  |  |  |  |  |  |  |  |
| miR-6838-5p | (324) |  |  |  |  |  |  |  |  |
| miR-7160 | (325) |  |  |  |  |  |  |  |  |

## Table S2. The tendency between genes affected by MPs in breast cancer and micro-RNAs that have demonstrated anti-breast cancer capability (kcal/mol).

| Miro-RNA | Gene | | | | | |
| --- | --- | --- | --- | --- | --- | --- |
|  | ABCB1 | ABCG2 | AP2M1 | FTH1 | PTP4A2 | TMBIM6 |
| miR-1 | -17.4 | -20.8 | -18.5 | -18.6 | -18.8 | -17.3 |
| miR-16-5p | -17.3 | -21.7 | -19.8 | -19.7 | -23.6 | -22.4 |
| miR-26b | -18.5 | -22.4 | -20.7 | -18.6 | -24.1 | -22.3 |
| miR-96-5p | -20.4 | -21.0 | -23.3 | -17.4 | -22.6 | -21.0 |
| miR-126-3p | -19.9 | -18.7 | -19.6 | -18.0 | -19.7 | -19.2 |
| miR-128-3p | -22.4 | -23.2 | -23.9 | -16.9 | -27.0 | -24.8 |
| miR-129-1-3p | -24.4 | -24.6 | -20.4 | -19.2 | -28.1 | -23.9 |
| miR-133 | -19.2 | -19.7 | -21.1 | -20.9 | -21.7 | -22.3 |
| miR-138-5p | -23.6 | -27.2 | -27.4 | -21.8 | -25.3 | -34.4 |
| miR-140-5p | -23.2 | -26.1 | -20.5 | -30.0 | -22.9 | -25.7 |
| miR-142-3p | -21.6 | -18.8 | -20.6 | -21.2 | -24.5 | -25.2 |
| miR-143-5p | -26.0 | -28.9 | -29.2 | -26.8 | -26.1 | -28.9 |
| miR-152 | -24.2 | -22.7 | -23.9 | -20.5 | -22.7 | -24.8 |
| miR-182-3p | -21.0 | -25.4 | -21.8 | -23.2 | -23.8 | -22.0 |
| miR-184 | -20.1 | -27.3 | -25.7 | -22.8 | -23.7 | -24.6 |
| miR-186 | -18.9 | -23.3 | -26.1 | -19.0 | -22.2 | -22.9 |
| miR-188-5p | -20.0 | -28.3 | -29.5 | -27.8 | -25.4 | -29.0 |
| miR-192-5p | -22.2 | -22.2 | -19.3 | -23.3 | -20.0 | -23.3 |
| miR-204 | -20.3 | -24.3 | -22.9 | -22.9 | -26.7 | -26.8 |
| miR-216a-5p | -23.2 | -25.0 | -24.1 | -23.2 | -25.7 | -25.6 |
| miR-216b | -20.5 | -21.2 | -19.0 | -20.0 | -23.0 | -24.1 |
| miR-322 | -19.1 | -18.5 | -16.7 | -19.7 | -23.1 | -20.0 |
| miR-331-3p | -25.1 | -27.8 | -28.7 | -24.3 | -29.8 | -28.2 |
| miR-342-3p | -17.8 | -23.5 | -21.3 | -19.0 | -28.0 | -26.2 |
| miR-365 | -26.8 | -31.8 | -27.8 | -23.4 | -29.6 | -28.9 |
| miR-374c-5p | -17.8 | -21.9 | -19.5 | -18.2 | -21.5 | -21.1 |
| miR-409 | -20.3 | -21.6 | -22.5 | -26.5 | -24.4 | -25.5 |
| miR-449a | -23.8 | -23.9 | -22.4 | -22.4 | -28.9 | -28.3 |
| miR-451a | -22.3 | -22.3 | -22.0 | -17.2 | -21.0 | -19.2 |
| miR-483-3p | -28.2 | -22.9 | -24.4 | -20.4 | -31.9 | -27.2 |
| miR-511 | -26.6 | -25.6 | -24.0 | -20.8 | -26.6 | -24.6 |
| miR-518a-5p | -17.2 | -21.5 | -29.8 | -24.9 | -22.0 | -23.3 |
| miR-519e-5p | -20.8 | -20.0 | -26.5 | -20.8 | -27.3 | -22.4 |
| miR-524-5p | -17.6 | -22.1 | -21.8 | -22.4 | -22.2 | -23.0 |
| miR-526b | -20.6 | -23.3 | -26.7 | -25.1 | -24.0 | -29.7 |
| miR-539 | -22.0 | -21.3 | -21.8 | -21.4 | -22.4 | -25.4 |
| miR-548c-5p | -19.3 | -18.6 | -18.6 | -17.1 | -20.2 | -19.9 |
| miR-641 | -22.1 | -21.7 | -23.7 | -27.4 | -24.6 | -23.7 |
| miR-1284 | -19.1 | -28.4 | -22.5 | -19.2 | -25.4 | -22.8 |
| miR-1298 | -21.2 | -22.5 | -23.1 | -21.7 | -25.0 | -23.9 |
| miR-3613-3p | -19.1 | -19.9 | -21.6 | -16.7 | -21.9 | -22.0 |
| miR-4458 | -22.1 | -24.7 | -23.8 | -21.5 | -23.7 | -24.0 |

## Table S3. The tendency between genes affected by MPs in Leukemia and microRNAs that have demonstrated anti-leukemia capability.

| Miro-RNA | Gene | | Miro-RNA | Gene | | Miro-RNA | Gene | |
| --- | --- | --- | --- | --- | --- | --- | --- | --- |
|  | ABCB1 | ABCG2 |  | ABCB1 | ABCG2 |  | ABCB1 | ABCG2 |
| miR-29b-3p | -18.9 | -22.9 | miR-182 | -22.4 | -22.9 | miR-361-3p | -20.2 | -25 |
| miR-34a | -20.7 | -24.6 | miR-203 | -20.8 | -24.8 | miR-370-3p | -24.7 | -24.9 |
| miR-34b | -22.7 | -22.5 | miR-211 | -21.2 | -23.4 | miR-376a-3p | -16.7 | -18.6 |
| miR-96 | -20.4 | -21 | miR-338-3p | -19.5 | -25.5 | miR-381-3p | -19.9 | -22.6 |
| miR-101 | -19.3 | -24.2 | miR-342-3p | -17.8 | -23.5 | miR-454-3p | -18.2 | -21.1 |
| miR-181a-5p | -19 | -21.7 | miR-342-5p | -20.2 | -23.7 | miR-520a | -21.5 | -26.6 |
|  |  |  |  |  |  | miR-582-3p | -18.2 | -24.6 |

## Table S4. The tendency between genes affected by MPs in liver cancer and microRNAs that have demonstrated anti-liver cancer capability.

| Gene | FABP1 | HO-1 | LXR-α | PPARα | PPARγ |
| --- | --- | --- | --- | --- | --- |
| Micro-RNA |  |  |  |  |  |
| miR-9 | -14.8 | -22.8 | -22.4 | -24.0 | -14.4 |
| miR-16-5p | -12.5 | -19.6 | -22.0 | -24.2 | -17.6 |
| miR-27a-3p | -12.1 | -23.1 | -23.9 | -26.4 | -20.2 |
| miR-29c-3p | -13.5 | -18.4 | -20.3 | -23.9 | -13.1 |
| miR-33b | -15.9 | -20.6 | -19.5 | -29.3 | -15.8 |
| miR-34a-5p | -12.4 | -23.9 | -29.8 | -31.7 | -26.8 |
| miR-101-3p | -15.1 | -18.4 | -21.2 | -24.7 | -17.6 |
| miR-122 | -18.2 | -24.3 | -23.1 | -25.7 | -17.9 |
| miR-125a-5p | -9.7 | -27.1 | -24.2 | -29.9 | -19.3 |
| miR-144-3p | -14.5 | -16.4 | -18.6 | -20.8 | -15.1 |
| miR-148a-3p | -15.4 | -21.9 | -23.8 | -26.1 | -17.9 |
| miR-152-3p | -16.2 | -18.8 | -25.0 | -24.5 | -16.2 |
| miR-155 | -14.8 | -20.9 | -21.7 | -25.1 | -16.8 |
| miR-181a | -15.9 | -26.1 | -20.4 | -27.4 | -15.6 |
| miR-199a | -14.2 | -27.0 | -23.4 | -29.5 | -20.9 |
| miR-200b | -14.5 | -23.8 | -22.9 | -28.8 | -19.7 |
| miR-297 | -14.6 | -23.2 | -19.9 | -32.5 | -22.0 |
| miR-302a | -14.8 | -20.9 | -21.1 | -22.9 | -14.4 |
| miR-329-3p | -12.8 | -22.6 | -19.2 | -26.9 | -15.4 |
| miR-339-5p | -12.4 | -32.4 | -30.6 | -37.3 | -16.5 |
| miR-342-3p | -14.6 | -26.4 | -27.8 | -24.5 | -15.0 |
| miR-370 | -13.7 | -23.8 | -23.1 | -29.6 | -17.8 |
| miR-383 | -13.7 | -24.1 | -21.5 | -26.8 | -22.4 |
| miR-429 | -11.6 | -21.8 | -18.8 | -22.1 | -13.8 |
| miR-455-3p | -14.8 | -23.0 | -22.2 | -27.3 | -16.9 |
| miR-485-5p | -12.4 | -29.5 | -22.1 | -28.8 | -19.2 |
| miR-486-5p | -10.8 | -27.9 | -27.7 | -33.5 | -14.5 |
| miR-490-5p | -11.3 | -23.9 | -27.3 | -28.8 | -16.3 |
| miR-495 | -11.3 | -22.5 | -21.6 | -25.8 | -13.6 |
| miR-499 | -14.8 | -20.5 | -20.6 | -20.9 | -14.8 |
| miR-559 | -13.2 | -14.9 | -15.7 | -23.6 | -14.6 |
| miR-597-5p | -14.3 | -23.7 | -20.9 | -28.7 | -16.9 |
| miR-627-5p | -13.2 | -20.3 | -21.2 | -23.1 | -19.0 |
| miR-638 | -20.0 | -37.4 | -33.9 | -39.3 | -29.2 |
| miR-936 | -17.1 | -27.2 | -23.9 | -30.8 | -20.0 |
| miR-1914 | -11.1 | -30.4 | -35.1 | -37.0 | -16.0 |
| miR-2392 | -15.6 | -26.6 | -27.1 | -32.3 | -20.8 |
| miR-4270-5p | -11.8 | -37.4 | -29.1 | -33.1 | -23.4 |
| miR-4310 | -11.5 | -18.7 | -19.0 | -24.7 | -12.4 |
| miR-4651 | -16.9 | -28.1 | -32.7 | -35.4 | -25.5 |

## Table S5. The tendency between genes affected by MPs in colorectal cancer and microRNAs that have demonstrated anti-colorectal cancer capability.

| Micro-RNA | Gene | | Micro-RNA | Gene | |
| --- | --- | --- | --- | --- | --- |
|  | MUC2 | TIM-4 |  | MUC2 | TIM-4 |
| miR-7 | -16.6 | -18.3 | miR-214 | -20.7 | -24.2 |
| miR-9-5p | -17.2 | -17.5 | miR-296 | -24.1 | -31.9 |
| miR-20b-5p | -22.4 | -21.5 | miR-328-3p | -26.1 | -24.4 |
| miR-22-3p | -20.8 | -18.6 | miR-335-5p | -14.3 | -13.5 |
| miR-30b-5p | -15.7 | -19.7 | miR-375-3p | -20.8 | -19.3 |
| miR-31 | -21.2 | -19.2 | miR-378a-5p | -18.8 | -22.4 |
| miR-93-5p | -22.8 | -25.8 | miR-488 | -15.4 | -16.1 |
| miR-101 | -18.9 | -18.5 | miR-498 | -23.3 | -21.6 |
| miR-124 | -20.1 | -18.6 | miR-506-3p | -22.2 | -22.0 |
| miR-124-3p | -24.8 | -24.1 | miR-509-5p | -21.0 | -18.7 |
| miR-125 | -19.2 | -21.1 | miR-582-5p | -15.9 | -16.4 |
| miR-129-5p | -20.8 | -18.1 | miR-760 | -38.4 | -23.7 |
| miR-130a-3p | -18.6 | -15.5 | miR-766-3p | -19.7 | -27.0 |
| miR-137-3p | -15.5 | -14.7 | miR-876-3p | -16.6 | -23.0 |
| miR-141-3p | -17.1 | -17.5 | miR-1266-3p | -21.3 | -21.2 |
| miR-142-3p | -18.9 | -20.2 | miR-3622a-3p | -19.4 | -22.6 |
| miR-144 | -13.8 | -16.3 | miR-4319 | -19.3 | -20.8 |
| miR-192 | -14.6 | -16.0 | miR-4429-5p | -30.8 | -18.8 |
| miR-193a | -23.3 | -22.6 | miR-4461 | -24.2 | -22.9 |
| miR-203a | -15.8 | -18.1 | miR-4746 | -21.5 | -23.1 |
| miR-205 | -16.4 | -23.3 | miR-6511b-5p | -31.9 | -23.1 |
| miR-212 | -18.3 | -20.9 |  |  |  |

## Table S6. The tendency between genes affected by microRNA (MPs) in lung cancer and microRNAs that have demonstrated anti-lung cancer capabilities.

| Micro-RNA | Genes | | | | | | | | | | |
| --- | --- | --- | --- | --- | --- | --- | --- | --- | --- | --- | --- |
|  | MAPK genes (326) | | | | | | | |  |  |  |
|  | BRAF | c-Jun | ERK | JNK | MAP2K1 | MAP2K4 | MAPK14 | KRAS | NLRP3 | BCL2 | TIM-4 |
| miR-1-3p | -19.4 | -18.6 | -21.2 | -20.6 | -17.5 | -19.3 | -18.6 | -20.5 | -18.8 | -24.1 | -15.9 |
| miR-16 | -22.3 | -23.1 | -26.7 | -22.5 | -21.3 | -22.7 | -22.9 | -25.6 | -21.9 | -24.1 | -15.0 |
| miR-23b | -30.1 | -26.7 | -28.6 | -26.6 | -26.2 | -26.0 | -29.6 | -25.8 | -26.8 | -26.5 | -22.8 |
| miR-29b | -28.0 | -24.3 | -26.9 | -26.2 | -24.3 | -26.1 | -27.6 | -30.8 | -25.0 | -28.9 | -18.2 |
| miR-30a-5p | -22.3 | -21.9 | -23.4 | -22.4 | -18.7 | -21.9 | -27.5 | -22.3 | -24.6 | -24.4 | -23.8 |
| miR-30e | -23.0 | -21.7 | -22.2 | -24.0 | -17.9 | -23.4 | -24.7 | -20.6 | -22.7 | -22.7 | -21.0 |
| miR-34b | -28.1 | -23.4 | -27.5 | -24.6 | -24.2 | -25.9 | -26.0 | -24.9 | -23.1 | -29.9 | -21.8 |
| miR-34b-3p | -27.3 | -27.7 | -26.4 | -24.7 | -22.3 | -21.9 | -24.7 | -22.5 | -21.2 | -25.5 | -17.5 |
| miR-34c | -26.7 | -24.3 | -24.9 | -29.3 | -27.3 | -26.8 | -25.3 | -25.9 | -22.0 | -29.9 | -17.9 |
| miR-92b | -30.5 | -33.8 | -34.1 | -29.2 | -31.7 | -27.3 | -30.2 | -29.1 | -33.1 | -32.3 | -21.8 |
| miR-125a | -27.4 | -31.9 | -29.2 | -28.4 | -21.1 | -24.7 | -26.9 | -24.8 | -24.9 | -28.9 | -21.1 |
| miR-129-2 | -29.3 | -28.0 | -25.9 | -23.9 | -21.7 | -19.8 | -26.6 | -25.5 | -20.1 | -27.3 | -23.3 |
| miR-139-5p | -25.3 | -28.3 | -28.0 | -29.9 | -26.9 | -26.7 | -24.7 | -27.6 | -24.3 | -28.4 | -24.0 |
| miR-140-3p | -26.7 | -21.4 | -29.4 | -22.6 | -26.1 | -27.0 | -24.5 | -27.5 | -22.5 | -27.3 | -14.0 |
| miR-142-3p | -22.7 | -25.8 | -25.9 | -22.6 | -20.9 | -23.5 | -20.4 | -22.0 | -21.5 | -22.5 | -20.2 |
| miR-144-3p | -16.1 | -22.1 | -20.4 | -21.0 | -19.1 | -21.0 | -19.3 | -18.4 | -15.9 | -21.7 | -12.6 |
| miR-146a-5p | -24.0 | -21.7 | -23.0 | -24.7 | -19.6 | -23.7 | -22.9 | -28.4 | -20.8 | -26.8 | -20.0 |
| miR-148b | -26.6 | -19.8 | -26.8 | -22.8 | -20.7 | -25.4 | -26.3 | -20.6 | -23.5 | -23.6 | -17.2 |
| miR-181c-5p | -25.2 | -22.1 | -25.9 | -25.4 | -20.8 | -26.7 | -23.1 | -24.3 | -25.1 | -25.4 | -20.9 |
| miR-181d-5p | -27.6 | -23.4 | -26.6 | -23.6 | -24.4 | -24.8 | -24.7 | -24.8 | -24.3 | -26.1 | -21.2 |
| miR-183-5p | -24.1 | -23.4 | -26.6 | -24.6 | -23.0 | -23.1 | -25.1 | -28.3 | -27.0 | -22.6 | -22.2 |
| miR-187 | -29.4 | -26.9 | -30.4 | -25.2 | -27.8 | -24.5 | -30.1 | -29.2 | -25.5 | -31.1 | -18.2 |
| miR-194 | -24.5 | -26.1 | -23.9 | -22.6 | -21.7 | -22.0 | -22.8 | -23.4 | -24.1 | -25.1 | -17.9 |
| miR-195-5p | -24.2 | -20.9 | -24.6 | -24.1 | -21.8 | -20.3 | -23.2 | -21.4 | -20.7 | -28.0 | -13.8 |
| miR-199a-3p | -25.1 | -24.8 | -26.1 | -23.8 | -23.2 | -28.7 | -23.6 | -24.3 | -22.7 | -24.9 | -19.1 |
| miR-200c-3p | -26.1 | -21.1 | -29.2 | -24.9 | -24.7 | -23.9 | -21.9 | -25.3 | -21.7 | -25.4 | -20.3 |
| miR-204 | -28.7 | -29.6 | -28.4 | -30.2 | -22.9 | -27.6 | -25.3 | -24.7 | -26.6 | -28.7 | -20.6 |
| miR-221 | -22.1 | -21.0 | -24.0 | -22.1 | -20.9 | -21.7 | -22.0 | -21.8 | -18.5 | -23.6 | -18.3 |
| miR-325 | -26.8 | -22.4 | -27.8 | -25.8 | -28.9 | -25.6 | -27.1 | -24.2 | -22.4 | -27.6 | -22.2 |
| miR-338-3p | -23.6 | -21.2 | -22.4 | -23.4 | -21.7 | -23.7 | -23.7 | -22.1 | -18.8 | -27.5 | -19.9 |
| miR-338-5p | -25.3 | -21.9 | -26.8 | -22.5 | -21.9 | -25.2 | -21.7 | -25.0 | -28.6 | -24.9 | -20.1 |
| miR-342-3p | -25.9 | -24.2 | -29.6 | -23.0 | -23.5 | -20.5 | -25.5 | -24.3 | -21.5 | -25.6 | -16.0 |
| miR-377 | -25.1 | -27.4 | -26.7 | -23.0 | -24.6 | -25.2 | -30.7 | -24.8 | -25.1 | -29.3 | -20.6 |
| miR-379 | -24.2 | -21.0 | -23.7 | -22.7 | -26.2 | -24.2 | -22.0 | -23.8 | -23.0 | -22.7 | -17.8 |
| miR-381 | -25.0 | -25.7 | -26.4 | -25.7 | -24.8 | -25.9 | -30.1 | -23.9 | -26.5 | -24.8 | -20.7 |
| miR-425-5p | -29.4 | -24.0 | -22.2 | -26.9 | -20.2 | -20.7 | -26.0 | -24.1 | -22.4 | -24.2 | -25.7 |
| miR-486-5p | -30.8 | -29.4 | -26.9 | -26.2 | -24.5 | -25.3 | -27.1 | -28.3 | -29.1 | -29.8 | -23.4 |
| miR-497 | -25.2 | -23.4 | -31.1 | -27.1 | -25.8 | -27.9 | -26.4 | -23.6 | -22.7 | -24.8 | -17.7 |
| miR-512-5p | -28.4 | -28.5 | -28.3 | -31.4 | -25.0 | -27.9 | -25.6 | -27.4 | -30.4 | -31.1 | -18.5 |
| miR-520a | -23.1 | -25.6 | -29.6 | -23.0 | -24.0 | -24.0 | -23.8 | -23.6 | -22.8 | -28.4 | -16.0 |
| miR-532-3p | -31.0 | -29.5 | -29.6 | -34.9 | -21.6 | -24.5 | -29.9 | -30.5 | -25.5 | -30.4 | -23.0 |
| miR-577 | -19.0 | -18.4 | -18.4 | -18.5 | -17.7 | -19.6 | -19.6 | -21.6 | -17.0 | -18.9 | -14.5 |
| miR-584-5p | -27.2 | -23.4 | -27.8 | -27.5 | -26.0 | -25.7 | -28.0 | -26.2 | -24.3 | -27.5 | -22.6 |
| miR-590 | -21.2 | -19.6 | -22.0 | -24.2 | -21.7 | -22.9 | -22.7 | -20.7 | -18.5 | -22.9 | -16.7 |
| miR-593-5p | -31.0 | -31.8 | -34.9 | -30.3 | -33.9 | -26.5 | -29.0 | -28.8 | -26.2 | -37.7 | -21.8 |
| miR-611 | -27.1 | -30.0 | -32.4 | -30.0 | -26.6 | -36.2 | -29.0 | -30.5 | -31.0 | -33.5 | -23.6 |
| miR-655-3p | -17.9 | -19.5 | -21.3 | -21.4 | -21.5 | -20.0 | -22.1 | -19.2 | -17.5 | -19.8 | -15.2 |
| miR-1915-3p | -26.8 | -31.9 | -29.3 | -33.8 | -30.2 | -25.2 | -27.1 | -24.9 | -28.9 | -33.5 | -20.8 |
| miR-4732-3p | -30.0 | -28.9 | -26.0 | -25.2 | -22.2 | -23.9 | -29.2 | -25.2 | -26.5 | -27.9 | -23.3 |

## Table S7. The tendency between genes affected by MPs in gastric cancer and microRNAs that have demonstrated anti-gastric cancer capability.

| Micro-RNA | Genes | | | |
| --- | --- | --- | --- | --- |
|  | ASGR2 | Bax | CD44 | TIM-4 |
| miR-1-3p | -13.1 | -14.1 | -18.7 | -15.9 |
| miR-9-5p | -19.5 | -16.1 | -23.8 | -17.5 |
| miR-19b-3p | -21.7 | -17.9 | -21.4 | -19.9 |
| miR-24 | -21.2 | -19.6 | -24.6 | -20 |
| miR-29c | -22.0 | -23.8 | -25.4 | -16.6 |
| miR-31 | -21.2 | -18.7 | -23.5 | -19.2 |
| miR-33a | -15.0 | -15.9 | -23.4 | -18.9 |
| miR-34a | -23.9 | -18.7 | -28.2 | -22 |
| miR-34c-5p | -22.6 | -16.5 | -27.7 | -17.9 |
| miR-105 | -27.2 | -23.2 | -25.7 | -20.9 |
| miR-107 | -20.9 | -16.7 | -28.1 | -16.8 |
| miR-124-5p | -21.7 | -19.2 | -26.4 | -18.6 |
| miR-126 | -14.6 | -16.9 | -17.4 | -14.7 |
| miR-129-3p | -23.1 | -25.0 | -27.6 | -21.3 |
| miR-129-5p | -22.8 | -16.6 | -27.5 | -18.1 |
| miR-140 | -20.9 | -19.0 | -27.3 | -22.5 |
| miR-148a-3p | -18.3 | -17.9 | -21.5 | -14.3 |
| miR-149 | -26.1 | -35.6 | -28.5 | -24.8 |
| miR-198 | -23.5 | -23.0 | -31.4 | -19.6 |
| miR-204-3p | -25.6 | -14.4 | -29.6 | -19.2 |
| miR-205-5p | -22.5 | -25.3 | -24.9 | -23.3 |
| miR-206 | -19.6 | -16.8 | -23.7 | -19.1 |
| miR-216b | -19.4 | -19.5 | -22.3 | -17.2 |
| miR‑299‑3p | -22.8 | -19.2 | -23.3 | -18.1 |
| miR-345 | -24.1 | -26.6 | -30.7 | -18.5 |
| miR-361-5p | -26.4 | -17.6 | -26.3 | -17.9 |
| miR-365 | -26.1 | -17.0 | -27.4 | -20.9 |
| miR-381 | -18.8 | -24.6 | -24.4 | -20.7 |
| miR-383-5p | -21.1 | -15.6 | -24.8 | -16.6 |
| miR-411 | -14.8 | -16.3 | -23.5 | -14.4 |
| miR-431-5p | -21.6 | -22.6 | -24.5 | -22.7 |
| miR-455-3p | -21.0 | -17.2 | -23.5 | -18.9 |
| miR-490-3p | -21.4 | -24.9 | -28.2 | -27.9 |
| miR-491 | -24.1 | -21.1 | -29.1 | -26.2 |
| miR-520-3p | -28.0 | -24.2 | -24.6 | -16 |
| miR-582-3p | -15.5 | -14.2 | -21.9 | -15.2 |
| miR-596 | -25.2 | -28.6 | -28.8 | -24.2 |
| miR-621 | -18.9 | -18.6 | -23.2 | -16.7 |
| miR-643 | -21.6 | -18.8 | -23.7 | -17.1 |
| miR-665 | -24.1 | -27.8 | -35.5 | -24.8 |
| miR-711 | -25.5 | -23.1 | -29.7 | -19.3 |
| miR-760 | -32.0 | -18.9 | -33.6 | -23.7 |
| miR-874-3p | -21.3 | -26.9 | -31.6 | -22.5 |
| miR-936 | -25.1 | -21.8 | -27.5 | -15 |
| miR-1179 | -20.2 | -16.3 | -21.7 | -15.8 |
| miR-1275 | -23.5 | -20.4 | -29.9 | -16.7 |
| miR-1286 | -18.4 | -23.9 | -26.7 | -18.3 |
| miR-1324 | -20.9 | -23.2 | -21.6 | -16.8 |
| miR-1915 | -23.5 | -27.2 | -32.7 | -20.9 |
| miR-4268 | -23.9 | -29.1 | -28.8 | -25 |
| miR-4429 | -24.0 | -14.5 | -28 | -18.8 |
| miR-5683 | -21.6 | -19.6 | -24 | -16 |
| miR-6838-5p | -20.4 | -17.3 | -25.2 | -22.4 |
| miR-7160 | -26.0 | -23.3 | -26.9 | -19.1 |

## Table S8. The tendency between genes affected by MPs in cervical cancer and microRNAs that have demonstrated anti-cervical cancer capability.

| Micro-RNA | Genes | Micro-RNA | Genes |
| --- | --- | --- | --- |
|  | TIM-4 |  | TIM-4 |
| miR-26b | -18.3 | miR-326 | -24.7 |
| miR-29a | -16.6 | miR-362-3p | -14.4 |
| miR-32-5p | -21.6 | miR-374b | -15.6 |
| miR-34c-5p | -17.9 | miR-377 | -20.6 |
| miR-100 | -20.4 | miR-520a-3p | -21.3 |
| miR-137 | -20.2 | miR-526b | -18 |
| miR-138 | -24.1 | miR-613 | -20.8 |
| miR-142-5p | -14.7 | miR-636 | -21.7 |
| miR-145 | -17.3 | miR-638 | -24.7 |
| miR-152 | -18.7 | miR-664 | -22.4 |
| miR-186-3p | -16.9 | miR-802 | -17.4 |
| miR-204 | -20.6 | miR-1258 | -24.2 |
| miR-214-3p | -20.5 | miR-3184-5p | -19.4 |
| miR-302c-3p | -25.1 | miR-3929 | -21.3 |

## Table S9. The tendency between genes affected by MPs in skin cancer and microRNAs that have demonstrated anti-skin cancer capability.

| Micro-RNA | Genes | Micro-RNA | Genes |
| --- | --- | --- | --- |
|  | NLRP3 |  | NLRP3 |
| miR-29c | -27.1 | miR-224-5p | -23.5 |
| miR-33a-5p | -19.6 | miR-365 | -28.9 |
| miR-127 | -27.6 | miR-429-5p | -26.9 |
| miR-130a | -26.9 | miR-524-5p | -20.5 |
| miR-135b-5p | -20.3 | miR-590-5p | -18.5 |
| miR-139-5p | -24.3 | miR-767-3p | -22.4 |
| miR-140-5p | -22.7 | miR-876-3p | -17.7 |
| miR-155 | -19.3 | miR-3619-5p | -28.7 |
| miR-193a-3p | -26.1 | miR-4458 | -21 |
| miR-200b-3p | -22 |  |  |

## Table S10. The tendency between genes affected by MPs in pancreatic cancer and microRNAs that have demonstrated anti-pancreatic cancer capability.

| Micro-RNA | Genes | Micro-RNA | Genes |
| --- | --- | --- | --- |
|  | TIM-4 |  | TIM-4 |
| miR-24-3p | -18.2 | miR‑381 | -20.7 |
| miR-26a | -19.6 | miR-382 | -18.8 |
| miR-33a-3p | -18.7 | miR-455 | -18.1 |
| miR-33a-5p | -18.9 | miR-497 | -17.7 |
| miR-139 | -24 | miR-519 | -19.9 |
| miR‑142‑5p | -14.7 | miR-519d-3p | -22 |
| miR-143-3p | -19.5 | miR-539 | -20.6 |
| miR-145 | -17.3 | miR-543 | -16.4 |
| miR-203 | -18.1 | miR-573 | -20.5 |
| miR-211-5p | -22.9 | miR-597-5p | -21.6 |
| miR-217 | -20.8 | miR-608 | -23.6 |
| miR-320b | -22.7 | miR-612 | -25.4 |
| miR-337 | -18 | miR-634 | -21 |
| miR-345-5p | -18.5 | miR-4269 | -23.2 |
| miR-374 | -16.5 |  |  |

# Reference

1. Tang Y-J, Wu W, Chen Q-Q, Liu S-H, Zheng Z-Y, Cui Z-L, et al. miR-29b-3p suppresses the malignant biological behaviors of AML cells via inhibiting NF-κB and JAK/STAT signaling pathways by targeting HuR. BMC cancer. 2022;22(1):909. doi:10.1186/s12885-022-09996-1

2. Yang L, Cai N, Zhao L. MicroRNA-1 regulates the growth and chemosensitivity of breast cancer cells by targeting MEK/ERK pathway. J buon. 2020;25(5):2215-20.

3. Huang L, Liu R, Zhou P, Tian Y, Lu Z. miR-9 and miR-181a target Gab2 to inhibit the proliferation and migration of hepatocellular carcinoma HepG2 cells. Genes. 2022;13(11):2152. doi:10.3390/genes13112152

4. Fan X, Liu M, Tang H, Leng D, Hu S, Lu R, et al. MicroRNA-7 exerts antiangiogenic effect on colorectal cancer via ERK signaling. J Surg Res. 2019;240:48-59. doi:10.1016/j.jss.2019.02.035

5. Lin Q. MicroRNA-1-3p affects lung adenocarcinoma progression through E2F8 and regulating NF-кB pathway. Cytokine. 2022;156:155922. doi:10.1016/j.cyto.2022.155922

6. Hu Y, Ma X, Wu Z, Nong Q, Liu F, Wang Y, et al. MicroRNA‐34a‐mediated death of acute myeloid leukemia stem cells through apoptosis induction and exosome shedding inhibition via histone deacetylase 2 targeting. IUBMB Life. 2020;72(7):1481-90. doi:10.1002/iub.2273

7. Najjary S, Mohammadzadeh R, Mansoori B, Vahidian F, Mohammadi A, Doustvandi MA, et al. Combination therapy with miR-34a and doxorubicin synergistically induced apoptosis in T-cell acute lymphoblastic leukemia cell line. Med Oncol. 2021;38:1-10. doi:10.1007/s12032-021-01578-8

8. Wang Z, Hu S, Li X, Liu Z, Han D, Wang Y, et al. MiR-16-5p suppresses breast cancer proliferation by targeting ANLN. BMC cancer. 2021;21:1-12. doi:10.1186/s12885-021-08914-1

9. Cheng B, Ding F, Huang C-Y, Xiao H, Fei F-Y, Li J. Role of miR-16-5p in the proliferation and metastasis of hepatocellular carcinoma. Eur Rev Med Pharmacol Sci. 2019;23(1). doi:10.26355/eurrev_201901_16757

10. Wang W, Yu H, Liu X. MiR-9-5p suppresses cell metastasis and epithelial-mesenchymal transition through targeting FOXP2 and predicts prognosis of colorectal carcinoma. Eur Rev Med Pharmacol Sci. 2019;23(15). doi:10.26355/eurrev_201908_18530

11. Wei J, Jia A, Ma L, Wang Y, Qiu L, Xiao B. MicroRNA-16 inhibits the proliferation and metastasis of human lung cancer cells by modulating the expression of YAP1. J BUON. 2020;25:862-8.

12. Qi H-X, Cao Q, Zhou G-P, Sun X-Z, Zhou W-D, Hong Z, et al. MicroRNA 34b inhibits cell proliferation in pediatric acute myeloid leukemia via regulating LDHA. Eur Rev Med Pharmacol Sci. 2019;23(12). doi:10.26355/eurrev_201906_18202

13. Ameli Mojarad M, Ameli Mojarad M, Pourmahdian A. MicroRNA-26b reduces cell viability by inhibition of nicotinamide phosphoribosyltransferase in breast cancer cells. DNA Cell Biol. 2022;41(8):735-41. doi:10.1089/dna.2022.0214

14. Wen M, Xu H, Peng H, Sheng Y, Yang W, Yan J. MiR‐27a‐3p targets USP46 to inhibit the cell proliferation of hepatocellular carcinoma. Chem Biol Drug Des. 2022;100(2):280-9. doi:10.1111/cbdd.14063

15. Yang H, Lin J, Jiang J, Ji J, Wang C, Zhang J. miR-20b-5p functions as tumor suppressor microRNA by targeting cyclinD1 in colon cancer. Cell Cycle. 2020;19(21):2939-54. doi:10.1080/15384101.2020.1829824

16. Wang H, Wang X, Fei J, Li F, Han J, Qin X. microRNA-23B inhibits non-small cell lung cancer proliferation, invasion and migration via downregulation of RUNX2 and inhibition of Wnt/Β-catenin signaling pathway. J Biol Regul Homeost Agents. 2020;34(3):825-35. doi:10.23812/20-11-A-34

17. Huang T, Fu Y, Wang S, Xu M, Yin X, Zhou M, et al. miR-96 acts as a tumor suppressor via targeting the BCR-ABL1 oncogene in chronic myeloid leukemia blastic transformation. Biomed Pharmacother. 2019;119:109413. doi:10.1016/j.biopha.2019.109413

18. Gao X-h, Zhang Y-l, Zhang Z-y, Guo S-s, Chen X-b, Guo Y-z. MicroRNA-96-5p represses breast cancer proliferation and invasion through Wnt/β-catenin signaling via targeting CTNND1. Sci Rep. 2020;10(1):44. doi:10.1038/s41598-019-56571-z

19. Lv T, Jiang L, Kong L, Yang J. MicroRNA‑29c‑3p acts as a tumor suppressor gene and inhibits tumor progression in hepatocellular carcinoma by targeting TRIM31. Oncol Rep. 2020;43(3):953-64. doi:10.3892/or.2020.7469

20. Jin R-R, Zeng C, Chen Y. MiR-22-3p regulates the proliferation, migration and invasion of colorectal cancer cells by directly targeting KDM3A through the Hippo pathway. Histol Histopathol. 2022. doi:10.14670/HH-18-526

21. Xie Y, Zhao F, Zhang P, Duan P, Shen Y. miR-29b inhibits non-small cell lung cancer progression by targeting STRN4. Hum Cell. 2020;33(1):220-31. doi:10.1007/s13577-019-00305-w

22. Yang X-Y, Sheng Y. miR-101 represses T-cell acute lymphoblastic leukemia by targeting CXCR7/STAT3 axis. Oncol Res. 2019;27(9):997. doi:10.3727/096504018X15439207752093

23. Hong Z, Hong C, Ma B, Wang Q, Zhang X, Li L, et al. MicroRNA‑126‑3p inhibits the proliferation, migration, invasion, and angiogenesis of triple‑negative breast cancer cells by targeting RGS3. Oncol Rep. 2019;42(4):1569-79. doi:10.3892/or.2019.7251

24. Wang H, Lin X, Liu E, Jian Z, Ou Y. MicroRNA‐33b regulates hepatocellular carcinoma cell proliferation, apoptosis, and mobility via targeting Fli‐1‐mediated Notch1 pathway. J Cell Physiol. 2020;235(10):7635-44. doi:10.1002/jcp.29673

25. Fan M, Ma X, Wang F, Zhou Z, Zhang J, Zhou D, et al. MicroRNA-30b-5p functions as a metastasis suppressor in colorectal cancer by targeting Rap1b. Cancer Lett. 2020;477:144-56. doi:10.1016/j.canlet.2020.02.021

26. Yang C, Huang T, Liang Y, Xue Y, Liang Y, Wei X, et al. CTHRC1 targeted by miR-30a-5p regulates cell adhesion, invasion and migration in lung adenocarcinoma. J Cardiothorac Surg. 2022;17(1):46. doi:10.1186/s13019-022-01788-9

27. Zhang X, Ma W, Xue W, Wang Y, Chen P, Li Q, et al. miR-181a plays the tumor-suppressor role in chronic myeloid leukemia CD34+ cells partially via SERPINE1. Cell Mol Life Sci. 2024;81(1):10. doi:10.1007/s00018-023-05036-8

28. Zhao J, Li D, Fang L. MiR-128-3p suppresses breast cancer cellular progression via targeting LIMK1. Biomed Pharmacother. 2019;115:108947. doi:10.1016/j.biopha.2019.108947

29. Xu X-P, Peng X-Q, Yin X-M, Liu Y, Shi Z-Y. miR-34a-5p suppresses the invasion and metastasis of liver cancer by targeting the transcription factor YY1 to mediate MYCT1 upregulation. Acta Histochem. 2020;122(6):151576. doi:10.1016/j.acthis.2020.151576

30. Hao C, Gao C, Shang H, Liu J, Qi F. MicroRNA-31 inhibits the growth and metastasis and enhances drug sensitivity of the human colon cancer cells by targeting PAX6. J BUON. 2020;25:1860-5.

31. Cui Y, Zhao L, Zhao S, Guo T, Li F, Li Z, et al. MicroRNA-30e inhibits proliferation and invasion of non-small cell lung cancer via targeting SOX9. Hum Cell. 2019;32:326-33. doi:10.1007/s13577-018-0223-0

32. Li D, Yuan Y, Meng C, Lin Z, Zhao M, Shi L, et al. Low expression of miR-182 caused by DNA hypermethylation accelerates acute lymphocyte leukemia development by targeting PBX3 and BCL2: miR-182 promoter methylation is a predictive marker for hypomethylation agents+ BCL2 inhibitor venetoclax. Clin Epigenetics. 2024;16(1):48. doi:10.1186/s13148-024-01658-2

33. Li Q, Gu Z, Tan Q, Ren L, Chen S. MicroRNA-129-1-3p represses the progression of triple-negative breast cancer by targeting the GRIN2D gene. Biomed Res Int. 2022;2022(1):1549357. doi:10.1155/2022/1549357

34. Liu Y, Tan J, Ou S, Chen J, Chen L. MicroRNA-101-3p suppresses proliferation and migration in hepatocellular carcinoma by targeting the HGF/c-Met pathway. Invest New Drugs. 2020;38(1):60-9. doi:10.1007/s10637-019-00766-8

35. Chen YL, Wang GX, Lin BA, Huang JS. MicroRNA‐93‐5p expression in tumor tissue and its tumor suppressor function via targeting programmed death ligand‐1 in colorectal cancer. Cell Biol Int. 2020;44(5):1224-36. doi:10.1002/cbin.11323

36. Zhuang X-F, Zhao L-X, Guo S-P, Wei S, Zhai J-F, Zhou Q-H. miR-34b inhibits the migration/invasion and promotes apoptosis of non-small-cell lung cancer cells by YAF2. Eur Rev Med Pharmacol Sci. 2019;23(5). doi:10.26355/eurrev_201903_17244

37. Qi S, Huang J, Long R. The synergistic effect of miR-203 and cytarabine on the inhibition of cell proliferation and induction of apoptosis in chronic myelogenous leukemia cells. Pak J Pharm Sci. 2024;37(5):1019-25.

38. Zhang G, Wang J, Zheng R, Song B, Huang L, Liu Y, et al. MiR-133 targets YES1 and inhibits the growth of triple-negative breast cancer cells. Technol Cancer Res Treat. 2020;19:1533033820927011. doi:10.1177/1533033820927011

39. Li X-N, Yang H, Yang T. miR-122 inhibits hepatocarcinoma cell progression by targeting LMNB2. Oncol Res. 2020;28(1):41. doi:10.3727/096504019X15615433287579

40. Huang Z, Wu X, Li J. miR-101 suppresses colon cancer cell migration through the regulation of EZH2. Rev Esp Enferm Dig. 2021;113:255-60. doi:10.17235/reed.2020.6800/2019

41. Feng H, Ge F, Du L, Zhang Z, Liu D. MiR‐34b‐3p represses cell proliferation, cell cycle progression and cell apoptosis in non‐small‐cell lung cancer (NSCLC) by targeting CDK4. J Cell Mol Med. 2019;23(8):5282-91. doi:10.1111/jcmm.14404

42. An L, Li X, Yang J. MicroRNA-211 attenuates cell proliferation in T-cell lymphoblastic lymphoma through targeting TCF12. Leuk Res. 2021;110:106653. doi:10.1016/j.leukres.2021.106653

43. Rasoolnezhad M, Safaralizadeh R, Hosseinpourfeizi MA, Banan-Khojasteh SM, Baradaran B. MiRNA-138-5p: A strong tumor suppressor targeting PD-L-1 inhibits proliferation and motility of breast cancer cells and induces apoptosis. Eur J Pharmacol. 2021;896:173933. doi:10.1016/j.ejphar.2021.173933

44. Xu X, Tao Y, Niu Y, Wang Z, Zhang C, Yu Y, et al. miR-125a-5p inhibits tumorigenesis in hepatocellular carcinoma. Aging (Albany NY). 2019;11(18):7639. doi:10.18632/aging.102276

45. Lu M-L, Zhang Y, Li J, Fu Y, Li W-H, Zhao G-F, et al. MicroRNA-124 inhibits colorectal cancer cell roliferation and suppresses tumor growth by interacting with PLCB1 and regulating Wnt/β-catenin signaling pathway. Eur Rev Med Pharmacol Sci. 2019;23(1). doi:10.26355/eurrev_201901_16756

46. Yang L-z, Lei C-c, Zhao Y-p, Sun H-w, Yu Q-h, Yang E-J, et al. MicroRNA-34c-3p target inhibiting NOTCH1 suppresses chemosensitivity and metastasis of non-small cell lung cancer. J Int Med Res. 2020;48(3):0300060520904847. doi:10.1177/0300060520904847

47. Yu K, Wang J, Hou J, Zhang L, Liang H. miR-338-3p plays a significant role in casticin-induced suppression of acute myeloid leukemia via targeting PI3K/Akt pathway. Biomed Res Int. 2022;2022(1):9214130. doi:10.1155/2022/9214130

48. Hou L, Liu Q, Zhao Y, Yang H, Meng Q, Yu F. MicroRNA-140-5p inhibits cellular proliferation, migration and invasion by downregulating AKT/STAT3/NF-κB pathway in breast carcinoma cells. Acta Pharm. 2022;72(4):587-97. doi:10.2478/acph-2022-0039

49. Xing B, Shen C, Yang Q, Wang Z, Tan W. miR‐144‐3p represses hepatocellular carcinoma progression by affecting cell aerobic glycolysis via FOXK1. Int J Exp Pathol. 2023;104(3):117-27. doi:10.1111/iep.12468

50. Roshani Asl E, Rasmi Y, Baradaran B. MicroRNA‐124‐3p suppresses PD‐L1 expression and inhibits tumorigenesis of colorectal cancer cells via modulating STAT3 signaling. J Cell Physiol. 2021;236(10):7071-87. doi:10.1002/jcp.30378

51. Chen L, Zhuo H-Z, Wu J-Y, Lin L-Y, Huang Z-L, Lu J-X, et al. MiR-92b inhibits proliferation and invasion of lung cancer by targeting EZH2. Eur Rev Med Pharmacol Sci. 2020;24(6). doi:10.26355/eurrev_202003_20683

52. Wang Y, Guo X, Wang L, Xing L, Zhang X, Ren J. miR-342-3p inhibits acute myeloid leukemia progression by targeting SOX12. Oxid Med Cell Longev. 2022;2022(1):1275141. doi:10.1155/2022/1275141

53. Mansoori B, Duijf PH, Mohammadi A, Safarzadeh E, Ditzel HJ, Gjerstorff MF, et al. MiR-142-3p targets HMGA2 and suppresses breast cancer malignancy. Life Sci. 2021;276:119431. doi:10.1016/j.lfs.2021.119431

54. Yuan X, Wen Y, Shi Q, Zhao Y, Ding J. MicroRNA-148a-3p suppresses the glycolysis and Cell proliferation by targeting transmembrane protein 54 in liver cancer. Biochem Biophys Res Commun. 2024;695:149424. doi:10.1016/j.bbrc.2023.149424

55. Yang M, Tang X, Wang Z, Wu X, Tang D, Wang D. miR-125 inhibits colorectal cancer proliferation and invasion by targeting TAZ. Biosci Rep. 2019;39(12):BSR20190193. doi:10.1042/BSR20190193

56. Huang H, Huang J, Yao J, Li N, Yang Z. miR‐125a regulates HAS1 and inhibits the proliferation, invasion and metastasis by targeting STAT3 in non–small cell lung cancer cells. J Cell Biochem. 2020;121(5-6):3197-207. doi:10.1002/jcb.29586

57. Wu Y-Y, Lai H-F, Huang T-C, Chen Y-G, Ye R-H, Chang P-Y, et al. Aberrantly reduced expression of miR-342-5p contributes to CCND1-associated chronic myeloid leukemia progression and imatinib resistance. Cell Death Dis. 2021;12(10):908. doi:10.1038/s41419-021-04209-2

58. Mansoori B, Kiani S, Mezajin AA, Zandi P, Banaie H, Rostamzadeh D, et al. MicroRNA-143-5p suppresses ER-positive breast cancer development by targeting oncogenic HMGA2. Clin Breast Cancer. 2023;23(7):e480-e90. e3. doi:10.1016/j.clbc.2023.07.011

59. Yin T, Liu M-M, Jin R-T, Kong J, Wang S-H, Sun W-B. miR-152-3p Modulates hepatic carcinogenesis by targeting cyclin-dependent kinase 8. Pathol Res Pract. 2019;215(6):152406. doi:10.1016/j.prp.2019.03.034

60. Wan P, Bai X, Yang C, He T, Luo L, Wang Y, et al. miR‐129‐5p inhibits proliferation, migration, and invasion in rectal adenocarcinoma cells through targeting E2F7. J Cell Physiol. 2020;235(7-8):5689-701. doi:10.1002/jcp.29501

61. Zhou W, Cai C, Lu J, Fan Q. miR‐129‐2 upregulation induces apoptosis and promotes NSCLC chemosensitivity by targeting SOX4. Thorac Cancer. 2022;13(7):956-64. doi:10.1111/1759-7714.14336

62. Xu D, Jiang J, He G, Zhou H, Ji C. KMT2A is targeted by miR-361-3p and modulates leukemia cell’s abilities to proliferate, migrate and invade. Hematology. 2023;28(1):2225341. doi:10.1080/16078454.2023.2225341

63. Maimaitiming A, Wusiman A, Aimudula A, Kuerban X, Su P. MicroRNA-152 inhibits cell proliferation, migration, and invasion in breast cancer. Oncol Res. 2020;28(1):13. doi:10.3727/096504019X15519249902838

64. Wang Q, Wang G-t, Lu W-h. MiR-155 inhibits malignant biological behavior of human liver cancer cells by regulating SRPK1. Technol Cancer Res Treat. 2021;20:1533033820957021. doi:10.1177/1533033820957021

65. Song G-L, Xiao M, Wan X-Y, Deng J, Ling J-D, Tian Y-G, et al. MiR-130a-3p suppresses colorectal cancer growth by targeting Wnt Family Member 1 (WNT1). Bioengineered. 2021;12(1):8407-18. doi:10.1080/21655979.2021.1977556

66. Sun T, Liu Z. MicroRNA-139-5p suppresses non-small cell lung cancer progression by targeting ATAD2. Pathol Res Pract. 2023;249:154719. doi:10.1016/j.prp.2023.154719

67. Li L, Luo F, Song X. MicroRNA-370-3p inhibits cell proliferation and induces chronic myelogenous leukemia cell apoptosis by suppressing PDLIM1/Wnt/ß-catenin signaling. Neoplasma. 2020;67(3). doi:10.4149/neo_2020_190612N506

68. Dinami R, Pompili L, Petti E, Porru M, D'Angelo C, Di Vito S, et al. MiR‐182‐3p targets TRF2 and impairs tumor growth of triple‐negative breast cancer. EMBO Mol Med. 2023;15(1):e16033. doi:10.15252/emmm.202216033

69. Ding X, Zhang J, Feng Z, Tang Q, Zhou X. MiR-137-3p inhibits colorectal cancer cell migration by regulating a KDM1A-dependent epithelial–mesenchymal transition. Dig Dis Sci. 2021;66:2272-82. doi:10.1007/s10620-020-06518-6

70. Huang H, Wang Y, Li Q, Fei X, Ma H, Hu R. miR-140-3p functions as a tumor suppressor in squamous cell lung cancer by regulating BRD9. Cancer Lett. 2019;446:81-9. doi:10.1016/j.canlet.2019.01.007

71. Xin X, Xu Z, Wei J, Zhang Y. MiR-376a-3p increases cell apoptosis in acute myeloid leukemia by targeting MT1X. Cancer Biol Ther. 2022;23(1):234-42. doi:10.1080/15384047.2022.2054243

72. Wang J. Tripterine and miR-184 show synergy to suppress breast cancer progression. Biochem Biophys Res Commun. 2021;561:19-25. doi:10.1016/j.bbrc.2021.04.108

73. Atta S, El Kramani N, Mohamed SR, Mohamed MA, Hassan SH, Hesham R, et al. MicroRNA-199: a potential therapeutic tool for hepatocellular carcinoma in an experimental model. Asian Pac J Cancer Prev. 2021;22(9):2771. doi:10.31557/APJCP.2021.22.9.2771

74. Xing Y, Jing H, Zhang Y, Suo J, Qian M. MicroRNA-141-3p affected proliferation, chemosensitivity, migration and invasion of colorectal cancer cells by targeting EGFR. Int J Biochem Cell Biol. 2020;118:105643. doi:10.1016/j.biocel.2019.105643

75. Jin Ce, Xiao L, Zhou Z, Zhu Y, Tian G, Ren S. MiR-142-3p suppresses the proliferation, migration and invasion through inhibition of NR2F6 in lung adenocarcinoma. Hum Cell. 2019;32:437-46. doi:10.1007/s13577-019-00258-0

76. Ye Q, Ying Q, Dai Q, Liao C, Xiao G. Tumor-suppressing effects of miR-381-3p in pediatric acute myeloid leukemia via ROCK1 downregulation. Funct Integr Genomics. 2023;23(1):43. doi:10.1007/s10142-022-00950-9

77. Sun Wj, Zhang Yn, Xue P. miR‐186 inhibits proliferation, migration, and epithelial‐mesenchymal transition in breast cancer cells by targeting Twist1. J Cell Biochem. 2019;120(6):10001-9. doi:10.1002/jcb.28283

78. Weijin F, Liying S, Zuojun L, Peipei M, Shanru Z, Shikun L. Effect of miRNA-200b on the proliferation of liver cancer cells via targeting SMYD2/p53 signaling pathway. Zhong Nan Da Xue Xue Bao Yi Xue Ban. 2022;47(10):1303. doi:10.11817/j.issn.1672-7347.2022.210521

79. Ren J, Li W, Pan G, Huang F, Yang J, Zhang H, et al. miR‐142‐3p modulates cell invasion and migration via PKM2‐mediated aerobic glycolysis in colorectal cancer. Anal Cell Pathol (Amst). 2021;2021(1):9927720. doi:10.1155/2021/9927720

80. Fang G, Zhang C, Liu Z, Peng Z, Tang M, Xue Q. MiR-144-3p inhibits the proliferation and metastasis of lung cancer A549 cells via targeting HGF. J Cardiothorac Surg. 2022;17(1):117. doi:10.1186/s13019-022-01861-3

81. Wang X, Zhong L, Dan W, Chu X, Luo X, Liu C, et al. MiR-454-3p promotes apoptosis and autophagy of AML cells by targeting ZEB2 and regulating AKT/mTOR pathway. Hematology. 2023;28(1):2223874. doi:10.1080/16078454.2023.2223874

82. Wang M, Zhang H, Yang F, Qiu R, Zhao X, Gong Z, et al. miR‐188‐5p suppresses cellular proliferation and migration via IL6ST: a potential noninvasive diagnostic biomarker for breast cancer. J Cell Physiol. 2020;235(5):4890-901. doi:10.1002/jcp.29367

83. Lu N, Min J, Peng L, Huang S, Chai X, Wang S, et al. MiR-297 inhibits tumour progression of liver cancer by targeting PTBP3. Cell Death Dis. 2023;14(8):564. doi:10.1038/s41419-023-06097-0

84. Cheng B, Zhang Y, Wu ZW, Cui ZC, Li WL. MiR-144 inhibits colorectal cancer cell migration and invasion by regulating PBX3. Eur Rev Med Pharmacol Sci. 2020;24(18):9361-9. doi:10.26355/eurrev_202009_23019

85. Iacona JR, Monteleone NJ, Lemenze AD, Cornett AL, Lutz CS. Transcriptomic studies provide insights into the tumor suppressive role of miR-146a-5p in non-small cell lung cancer (NSCLC) cells. RNA Biol. 2019;16(12):1721-32. doi:10.1080/15476286.2019.1657351

86. Xiao J, Wan F, Tian L, Li Y. Tumor suppressor miR-520a inhibits cell growth by negatively regulating PI3K/AKT signaling pathway in acute myeloid leukemia. Adv Clin Exp Med. 2024;33(7):729-38. doi:10.17219/acem/171299

87. Chen P, Feng Y, Zhang H, Shi X, Li B, Ju W, et al. MicroRNA‑192 inhibits cell proliferation and induces apoptosis in human breast cancer by targeting caveolin 1. Oncol Rep. 2019;42(5):1667-76. doi:10.3892/or.2019.7298

88. Wang M, Lv G, Jiang C, Xie S, Wang G. miR-302a inhibits human HepG2 and SMMC-7721 cells proliferation and promotes apoptosis by targeting MAP3K2 and PBX3. Sci Rep. 2019;9(1):2032. doi:10.1038/s41598-018-38435-0

89. Zheng X-F, Liu K-X, Wang X-M, Zhang R, Li X. MicroRNA-192 acts as a tumor suppressor in colon cancer and simvastatin activates miR-192 to inhibit cancer cell growth. Mol Med Rep. 2019;19(3):1753-60. doi:10.3892/mmr.2019.9808

90. Jiang Z, Zhang J, Chen F, Sun Y. MiR‐148b suppressed non‐small cell lung cancer progression via inhibiting ALCAM through the NF‐κB signaling pathway. Thorac Cancer. 2020;11(2):415-25. doi:10.1111/1759-7714.13285

91. Li H, Tian X, Wang P, Huang M, Xu R, Nie T. MicroRNA-582–3p negatively regulates cell proliferation and cell cycle progression in acute myeloid leukemia by targeting cyclin B2. Cell Mol Biol Lett. 2019;24:1-12. doi:10.1186/s11658-019-0184-7

92. Vajen B, Greiwe L, Schäffer V, Eilers M, Huge N, Stalke A, et al. MicroRNA‐192‐5p inhibits migration of triple negative breast cancer cells and directly regulates Rho GTPase activating protein 19. Genes Chromosomes Cancer. 2021;60(11):733-42. doi:10.1002/gcc.22982

93. Xin R-Q, Li W-B, Hu Z-W, Wu Z-X, Sun W. MiR-329-3p inhibits hepatocellular carcinoma cell proliferation and migration through USP22-Wnt/β-Catenin pathway. Eur Rev Med Pharmacol Sci. 2020;24(19). doi:10.26355/eurrev_202010_23204

94. Hejazi M, Baghbani E, Amini M, Rezaei T, Aghanejad A, Mosafer J, et al. MicroRNA‐193a and taxol combination: A new strategy for treatment of colorectal cancer. J Cell Biochem. 2020;121(2):1388-99. doi:10.1002/jcb.29374

95. Wang J, Li M, Wang M, Yang J, Li D, Hao Y. MiR-181c-5p regulates lung adenocarcinoma progression via targeting PRKN. Biochem Genet. 2024;62(2):1103-14. doi:10.1007/s10528-023-10459-w

96. Fan X, Fang X, Liu G, Xiong Q, Li Z, Zhou W. MicroRNA-204 inhibits the proliferation and metastasis of breast cancer cells by targeting PI3K/AKT pathway. J buon. 2019;24(3):1054-9.

97. Wang J, Lu L, Luo Z, Li W, Lu Y, Tang Q, et al. miR-383 inhibits cell growth and promotes cell apoptosis in hepatocellular carcinoma by targeting IL-17 via STAT3 signaling pathway. Biomed Pharmacother. 2019;120:109551. doi:10.1016/j.biopha.2019.109551

98. Miao J, Hou N, Yang W, Jiang Q, Xue W, Wang X, et al. miR-203a suppresses cell proliferation by targeting RING-finger protein 6 in colorectal cancer. Anti-Cancer Drugs. 2020;31(6):583-91. doi:10.1097/CAD.0000000000000874

99. Gao L-M, Zheng Y, Wang P, Zheng L, Zhang W-L, Di Y, et al. Tumor-suppressive effects of microRNA-181d-5p on non-small-cell lung cancer through the CDKN3-mediated Akt signaling pathway in vivo and in vitro. Am J Physiol Lung Cell Mol Physiol. 2019;316(5):L918-L33. doi:10.1152/ajplung.00334.2018

100. Cui Y, Wang J, Liu S, Qu D, Jin H, Zhu L, et al. miR‐216a promotes breast cancer cell apoptosis by targeting PKC α. Fundam Clin Pharmacol. 2019;33(4):397-404. doi:10.1111/fcp.12481

101. Zeng H, Zheng J, Wen S, Luo J, Shao G, Zhang Y. MicroRNA-339 inhibits human hepatocellular carcinoma proliferation and invasion via targeting ZNF689. Drug Des Devel Ther. 2019:435-45. doi:10.2147/DDDT.S186352

102. Fan Y, Wang K. miR‑205 suppresses cell migration, invasion and EMT of colon cancer by targeting mouse double minute 4. Mol Med Rep. 2020;22(2):633-42. doi:10.3892/mmr.2020.11150

103. Han P, Zhang B, Li Y, Gao R, Li X, Ren H, et al. MiR-183-5p inhibits lung squamous cell carcinoma survival through disrupting hypoxia adaptation mediated by HIF-1α/NDUFA4L2 axis. Oncogene. 2024;43(38):2821-34. doi:10.1038/s41388-024-03129-7

104. Zhang Y, Lin P, Zou J-Y, Zou G, Wang W-Z, Liu Y-L, et al. MiR-216a-5p act as a tumor suppressor, regulating the cell proliferation and metastasis by targeting PAK2 in breast cancer. Eur Rev Med Pharmacol Sci. 2019;23(6). doi:10.26355/eurrev_201903_17394

105. Komoll R-M, Hu Q, Olarewaju O, von Döhlen L, Yuan Q, Xie Y, et al. MicroRNA-342-3p is a potent tumour suppressor in hepatocellular carcinoma. J Hepatol. 2021;74(1):122-34. doi:10.1016/j.jhep.2020.07.039

106. Mou T-Y, Zhang R-R, Wang Y-N. MiRNA-212 acts as a tumor-suppressor in colorectal carcinoma through targeting SOX4. Eur Rev Med Pharmacol Sci. 2019;23(24). doi:10.26355/eurrev_201912_19777

107. Liang Z, Xu J, Ma Z, Li G, Zhu W. MiR-187 suppresses non-small-cell lung cancer cell proliferation by targeting FGF9. Bioengineered. 2020;11(1):70-80. doi:10.1080/21655979.2019.1706287

108. Liu T, Ye P, Ye Y, Han B. MicroRNA-216b targets HK2 to potentiate autophagy and apoptosis of breast cancer cells via the mTOR signaling pathway. Int J Biol Sci. 2021;17(11):2970. doi:10.7150/ijbs.48933

109. He Y, He X. MicroRNA-370 regulates cellepithelial-mesenchymal transition, migration, invasion, and prognosis of hepatocellular carcinoma by targeting GUCD1. Yonsei Med J. 2019;60(3):267. doi:10.3349/ymj.2019.60.3.267

110. Shan H, Zhou X, Chen C. MicroRNA‑214 suppresses the viability, migration and invasion of human colorectal carcinoma cells via targeting transglutaminase 2. Mol Med Rep. 2019;20(2):1459-67. doi:10.3892/mmr.2019.10325

111. Meng X, Li Z, Zhou S, Xiao S, Yu P. miR‐194 suppresses high glucose‐induced non‐small cell lung cancer cell progression by targeting NFAT5. Thorac Cancer. 2019;10(5):1051-9. doi:10.1111/1759-7714.13038

112. Hu J, Guo P, Zhang Y, Huang Z, Chen B. MicroRNA-322 regulates the growth, chemosensitivity, migration and invasion of breast cancer cells by targeting NF-kB1. J BUON. 2020;25(1):152-8.

113. Yu X, Fan X, Zhang X, Wei P, Zhou H, Liu D, et al. miR-429 inhibits the formation of an immunosuppressive microenvironment to counteract hepatocellular carcinoma immune escape by targeting PD-L1. Funct Integr Genomics. 2023;23(4):312. doi:10.1007/s10142-023-01231-9

114. Zhang Z, Zhong X, Xiao Y, Chen C. MicroRNA-296 inhibits colorectal cancer cell growth and enhances apoptosis by targeting ARRB1-mediated AKT activation. Oncol Rep. 2019;41(1):619-29. doi:10.3892/or.2018.6806

115. Long Z, Wang Y. miR-195-5p suppresses lung cancer cell proliferation, migration, and invasion via FOXK1. Technol Cancer Res Treat. 2020;19:1533033820922587. doi:10.1177/1533033820922587

116. Zhao M, Zhang M, Tao Z, Cao J, Wang L, Hu X. miR-331-3p Suppresses Cell Proliferation in TNBC Cells by Downregulating NRP2. Technol Cancer Res Treat. 2020;19:1533033820905824. doi:10.1177/1533033820905824

117. Ma F, Huang J, Li W, Li P, Liu M, Xue H. MicroRNA‐455‐3p functions as a tumor suppressor by targeting HDAC2 to regulate cell cycle in hepatocellular carcinoma. Environ Toxicol. 2022;37(7):1675-85. doi:10.1002/tox.23516

118. Pan S, Ren F, Li L, Liu D, Li Y, Wang A, et al. MiR-328-3p inhibits cell proliferation and metastasis in colorectal cancer by targeting Girdin and inhibiting the PI3K/Akt signaling pathway. Exp Cell Res. 2020;390(1):111939. doi:10.1016/j.yexcr.2020.111939

119. Liu H, Wang Y, Wang Y, Wu D, Zhang H. miR-199a-3p plays an anti-tumorigenic role in lung adenocarcinoma by suppressing anterior gradient 2. Bioengineered. 2021;12(1):7859-71. doi:10.1080/21655979.2021.1967009

120. Liu C, Xing H, Luo X, Wang Y. MicroRNA-342 targets Cofilin 1 to suppress the growth, migration and invasion of human breast cancer cells. Arch Biochem Biophys. 2020;687:108385. doi:10.1016/j.abb.2020.108385

121. Gao J, Dai C, Yu X, Yin X-B, Zhou F. microRNA-485-5p inhibits the progression of hepatocellular carcinoma through blocking the WBP2/Wnt signaling pathway. Cell Signal. 2020;66:109466. doi:10.1016/j.cellsig.2019.109466

122. Zhang D, Yang N. MiR-335-5p inhibits cell proliferation, migration and invasion in colorectal cancer through downregulating LDHB. J buon. 2019;24(3):1128-36.

123. Yi X, Chen X, Li Z. miR-200c targeting GLI3 inhibits cell proliferation and promotes apoptosis in non-small cell lung cancer cells. Medicine (Baltimore). 2024;103(38):e39658. doi:10.1097/MD.0000000000039658

124. Liu F, Zhuang L, Wu RX, Li DY. miR-365 inhibits cell invasion and migration of triple negative breast cancer through ADAM10. Journal of Buon. 2019;24(5):1905-12.

125. Yan X, Liu X, Wang Z, Cheng Q, Ji G, Yang H, et al. MicroRNA‑486‑5p functions as a tumor suppressor of proliferation and cancer stem‑like cell properties by targeting Sirt1 in liver cancer. Oncol Rep. 2019;41(3):1938-48. doi:10.3892/or.2018.6930

126. Xu X, Chen X, Xu M, Liu X, Pan B, Qin J, et al. miR-375-3p suppresses tumorigenesis and partially reverses chemoresistance by targeting YAP1 and SP1 in colorectal cancer cells. Aging (Albany NY). 2019;11(18):7357. doi:10.18632/aging.102214

127. Li P, Wang Q, Wang H. MicroRNA-204 inhibits the proliferation, migration and invasion of human lung cancer cells by targeting PCNA-1 and inhibits tumor growth in vivo. Int J Mol Med. 2019;43(3):1149-56. doi:10.3892/ijmm.2018.4044

128. Hao S, Tian W, Chen Y, Wang L, Jiang Y, Gao B, et al. MicroRNA‐374c‐5p inhibits the development of breast cancer through TATA‐box binding protein associated factor 7‐mediated transcriptional regulation of DEP domain containing 1. J Cell Biochem. 2019;120(9):15360-8. doi:10.1002/jcb.28803

129. Yu Y, Cai O, Wu P, Tan S. MiR‐490‐5p inhibits the stemness of hepatocellular carcinoma cells by targeting ECT2. J Cell Biochem. 2019;120(1):967-76. doi:10.1002/jcb.27459

130. Li K, Zhang J, Zhang M, Wu Y, Lu X, Zhu Y. miR-378a-5p inhibits the proliferation of colorectal cancer cells by downregulating CDK1. World J Surg Oncol. 2021;19:1-13. doi:10.1186/s12957-021-02166-w

131. Sun Y-J, Li J, Chen C-H. Effects of miR-221 on the apoptosis of non-small cell lung cancer cells by lncRNA HOTAIR. Eur Rev Med Pharmacol Sci. 2019;23(10). doi:10.26355/eurrev_201905_17927

132. Chen Z, Sang M-X, Geng C-Z, Jia H-Q. MicroRNA-409 regulates the proliferation and invasion of breast cancer cell lines by targeting special AT-rich sequence-binding protein 1 (SATB1). Bioengineered. 2022;13(5):13045-54. doi:10.1080/21655979.2022.2073320

133. Zhang R, Guo C, Liu T, Li W, Chen X. MicroRNA miR-495 regulates the development of Hepatocellular Carcinoma by targeting C1q/tumor necrosis factor-related protein-3 (CTRP3). Bioengineered. 2021;12(1):6902-12. doi:10.1080/21655979.2021.1973878

134. Wang Y-B, Shi Q, Li G, Zheng J-H, Lin J, Qiu W. MicroRNA-488 inhibits progression of colorectal cancer via inhibition of the mitogen-activated protein kinase pathway by targeting claudin-2. Am J Physiol Cell Physiol. 2019;316(1):C33-C47. doi:10.1152/ajpcell.00047.2018

135. Yu M, Li L, Xu P. miR-325 suppresses cell proliferation and migration in non-small cell lung cancer via targeting DNA ligase 1 (LIG1). Folia Biol (Praha). 2024;70(2). doi:10.14712/fb2024070020095

136. Vajen B, Bhowmick R, Greiwe L, Schäffer V, Eilers M, Reinkens T, et al. MicroRNA-449a inhibits triple negative breast cancer by disturbing DNA repair and chromatid separation. Int J Mol Sci. 2022;23(9):5131. doi:10.3390/ijms23095131

137. Wang J, Li J, Chen L, Fan Z, Cheng J. MicroRNA-499 suppresses the growth of hepatocellular carcinoma by downregulating astrocyte elevated gene-1. Technol Cancer Res Treat. 2020;19:1533033820920253. doi:10.1177/1533033820920253

138. Wang T, Ma L, Li W, Ding L, Gao H. MicroRNA‐498 reduces the proliferation and invasion of colorectal cancer cells via targeting Bcl‐2. FEBS Open Bio. 2020;10(1):168-75. doi:10.1002/2211-5463.12767

139. Zhang B, Wang D, Wang Y, Chen G. miRNA-338-3p inhibits the migration, invasion and proliferation of human lung adenocarcinoma cells by targeting MAP3K2. Aging (Albany NY). 2022;14(15):6094. doi:10.18632/aging.204198

140. Zhang H, Chen P, Yang J. miR-451a suppresses the development of breast cancer via targeted inhibition of CCND2. Mol Cell Probes. 2020;54:101651. doi:10.1016/j.mcp.2020.101651

141. Wang C, Li C, Hao R. miR-559 inhibits proliferation, autophagy, and angiogenesis of hepatocellular carcinoma cells by targeting PARD3. Mediators Inflamm. 2022;2022(1):3121492. doi:10.1155/2022/3121492

142. Ai L, Luo X, Yan X, Jiang S. MicroRNA-506-3p inhibits colorectal cancer cell proliferation through targeting enhancer of zeste homologue 2. Bioengineered. 2021;12(1):4044-53. doi:10.1080/21655979.2021.1951930

143. Wu H, Li F, Zhu R. miR-338-5p inhibits cell growth and migration via inhibition of the METTL3/m6A/c-Myc pathway in lung cancer. Acta Biochim Biophys Sin (Shanghai). 2021;53(3):304-16. doi:10.1093/abbs/gmaa170

144. Menbari MN, Rahimi K, Ahmadi A, Mohammadi-Yeganeh S, Elyasi A, Darvishi N, et al. miR-483-3p suppresses the proliferation and progression of human triple negative breast cancer cells by targeting the HDAC8>oncogene. J Cell Physiol. 2020;235(3):2631-42. doi:10.1002/jcp.29167

145. Sun X, Hou Z, Li N, Zhang S. MiR-597-5p suppresses the progression of hepatocellular carcinoma via targeting transcriptional enhancer associate domain transcription factor 1 (TEAD1). In Vitro Cell Dev Biol Anim. 2022;58(2):96-108. doi:10.1007/s11626-021-00614-1

146. Elrebehy MA, Abdelghany TM, Elshafey MM, Gomaa MH, Doghish AS. miR-509–5p promotes colorectal cancer cell ferroptosis by targeting SLC7A11. Pathol Res Pract. 2023;247:154557. doi:10.1016/j.prp.2023.154557

147. Chen Z, Ying J, Shang W, Ding D, Guo M, Wang H. miR-342-3p regulates the proliferation and apoptosis of NSCLC cells by targeting BCL-2. Technol Cancer Res Treat. 2021;20:15330338211041193. doi:10.1177/15330338211041193

148. Zhang C, Yang T, Jiang H. miR‐511 inhibits proliferation and metastasis of breast cancer cells by targeting FGF4. J Gene Med. 2020;22(9):e3168. doi:10.1002/jgm.3168

149. Wang J, Chen T, Wang L, Yao B, Sun L, Chen S, et al. MicroRNA‐627‐5p inhibits the proliferation of hepatocellular carcinoma cells by targeting BCL3 transcription coactivator. Clin Exp Pharmacol Physiol. 2020;47(3):485-94. doi:10.1111/1440-1681.13218

150. Xiao W, Zhou H, Chen B, Shen B, Zhou J. miR-582-5p inhibits migration and chemo-resistant capabilities of colorectal cancer cells by targeting TNKS2. Genes Genomics. 2022;44(6):747-56. doi:10.1007/s13258-021-01141-9

151. Hashemi S, Yari N, Rahimi Jamnani F, Mahdian R, Karimi M, Zeinali S, et al. The role of miRNA-377 as a tumor suppressor in lung cancer by negative regulation of genes belonging to ErbB signaling pathway. Mol Biol Rep. 2022:1-11. doi:10.1007/s11033-021-06844-6

152. Wang L, Guo Z, Zhang S, Zhang X. miR-518a-5p targets ZEB2 to suppress the migration and invasion of breast-cancer cells. Altern Ther Health Med. 2023;29(1):137-43.

153. Zhang H, Liang H, Wu S, Zhang Y, Yu Z. MicroRNA-638 induces apoptosis and autophagy in human liver cancer cells by targeting enhancer of zeste homolog 2 (EZH2). Environ Toxicol Pharmacol. 2021;82:103559. doi:10.1016/j.etap.2020.103559

154. Ma H, Li M, Jia Z, Chen X, Bu N. miR-876-3p suppresses the progression of colon cancer and correlates the prognosis of patients. Exp Mol Pathol. 2021;122:104682. doi:10.1016/j.yexmp.2021.104682

155. Liu B, Wang Z, Cheng S, Du L, Yin Y, Yang Z, et al. miR‑379 inhibits cell proliferation and epithelial‑mesenchymal transition by targeting CHUK through the NF‑κB pathway in non‑small cell lung cancer. Mol Med Rep. 2019;20(2):1418-28. doi:10.3892/mmr.2019.10362

156. Ma S, Pu C. MiR-519e-5p regulates malignant phenotype of breast cancer cells through binding to CTPS1. Exp Cell Res. 2024;442(2):114225. doi:10.1016/j.yexcr.2024.114225

157. Tian J, Zhao Y, Li L, Cui Y, Wu Y. MicroRNA-936 targets JAG1 and inhibits the proliferation of hepatocellular carcinoma cells. Technol Cancer Res Treat. 2021;20:1533033820985785. doi:10.1177/1533033820985785

158. Cong K, Li C-G, Wei Y-H, Zhang K, Xu H-B. MicroRNA-760 inhibits the biological progression of colorectal carcinoma by directly targeting FOXA1 and regulating epithelial-tomesenchymal transition and PI3K/AKT signaling pathway. Eur Rev Med Pharmacol Sci. 2019;23(13). doi:10.26355/eurrev_201907_18310

159. Cui F, Luo P, Wu R, Meng J. miR‐381 Inhibits Proliferation and Invasion of Non‐Small‐Cell Cancer Cells by Targeting USP39. Dis Markers. 2022;2022(1):2195393. doi:10.1155/2022/2195393

160. Jin T, Zhang Y, Zhang T. MiR-524-5p suppresses migration, invasion, and EMT progression in breast cancer cells through targeting FSTL1. Cancer Biother Radiopharm. 2020;35(10):789-801. doi:10.1089/cbr.2019.3046

161. Sun L, Wang L, Chen T, Yao B, Wang Y, Li Q, et al. microRNA‐1914, which is regulated by lncRNA DUXAP10, inhibits cell proliferation by targeting the GPR39‐mediated PI3K/AKT/mTOR pathway in HCC. J Cell Mol Med. 2019;23(12):8292-304. doi:10.1111/jcmm.14705

162. He X-X, Luo S-S, Qin H-Q, Mo X-W. MicroRNA-766-3p-mediated downregulation of HNF4G inhibits proliferation in colorectal cancer cells through the PI3K/AKT pathway. Cancer Gene Ther. 2022;29(6):803-13. doi:10.1038/s41417-021-00362-0

163. Yu X, Zheng H, Sun R, Qian X, Jiang P, Yang B, et al. MicroRNA-425-5p inhibits lung cancer cell growth in vitro and in vivo by downregulating TFIIB-related factor 2. Technol Cancer Res Treat. 2020;19:1533033819901115. doi:10.1177/1533033819901115

164. Liu Y-Q, Cong Y-Z, Jiang J, Sheng J-Z, Li X-H, Zhao M, et al. MiR-526b suppresses cell proliferation, cell invasion and epithelial-mesenchymal transition in breast cancer by targeting Twist1. Eur Rev Med Pharmacol Sci. 2020;24(6). doi:10.26355/eurrev_202003_20678

165. Sun B, Ji W, Liu C, Lin X, Chen L, Qian H, et al. miR‐2392 functions as tumour suppressor and inhibits malignant progression of hepatocellular carcinoma via directly targeting JAG2. Liver Int. 2022;42(7):1658-73. doi:10.1111/liv.15284

166. Zhou H, Huang S, Shao C, Zou J, Zhou A, Yu J, et al. MiR-1266-3p suppresses epithelial-mesenchymal transition in colon cancer by targeting P4HA3. Anal Cell Pathol (Amst). 2022;2022(1):1542117. doi:10.1155/2022/1542117

167. Wei D. MiR-486-5p specifically suppresses SAPCD2 expression, which attenuates the aggressive phenotypes of lung adenocarcinoma cells. Histol Histopathol. 2022. doi:10.14670/HH-18-463

168. Cai F, Chen L, Sun Y, He C, Fu D, Tang J. MiR-539 inhibits the malignant behavior of breast cancer cells by targeting SP1. Biochem Cell Biol. 2020;98(3):426-33. doi:10.1139/bcb-2019-0111

169. Wang Y, Li C-f, Sun L-b, Li Y-c. microRNA-4270-5p inhibits cancer cell proliferation and metastasis in hepatocellular carcinoma by targeting SATB2. Hum Cell. 2020;33(4):1155-64. doi:10.1007/s13577-020-00384-0

170. Chang S, Sun G, Zhang D, Li Q, Qian H. MiR-3622a-3p acts as a tumor suppressor in colorectal cancer by reducing stemness features and EMT through targeting spalt-like transcription factor 4. Cell Death Dis. 2020;11(7):592. doi:10.1038/s41419-020-02789-z

171. Xia Y, Hu C, Lian L, Hui K, Wang L, Qiao Y, et al. miR‑497 suppresses malignant phenotype in non‑small cell lung cancer via targeting KDR. Oncol Rep. 2019;42(1):443-52. doi:10.3892/or.2019.7163

172. He S, Wang Q. MicroRNA-548c-5p inhibits the proliferation of breast cancer cells through regulating Wnt/β-catenin signaling pathway. Eur Rev Med Pharmacol Sci. 2020;24(7):3795-804. doi:10.26355/eurrev_202004_20845

173. Li H, Chen Z, Zhang Y, Yuan P, Liu J, Ding L, et al. MiR-4310 regulates hepatocellular carcinoma growth and metastasis through lipid synthesis. Cancer Lett. 2021;519:161-71. doi:10.1016/j.canlet.2021.07.029

174. Huang L, Zhang Y, Li Z, Zhao X, Xi Z, Chen H, et al. MiR-4319 suppresses colorectal cancer progression by targeting ABTB1. United European Gastroenterol J. 2019;7(4):517-28. doi:10.1177/2050640619837440

175. Cao B, Tan S, Tang H, Chen Y, Shu P. miR‑512‑5p suppresses proliferation, migration and invasion, and induces apoptosis in non‑small cell lung cancer cells by targeting ETS1. Mol Med Rep. 2019;19(5):3604-14. doi:10.3892/mmr.2019.10022

176. Li L, Wei D, Zhang J, Deng R, Tang J, Su D. miR‐641 inhibited cell proliferation and induced apoptosis by targeting NUCKS1/PI3K/AKT signaling pathway in breast cancer. Comput Math Methods Med. 2022;2022(1):5203839. doi:10.1155/2022/5203839

177. Li Y, Wang X, Li Z, Liu B, Wu C. MicroRNA-4651 represses hepatocellular carcinoma cell growth and facilitates apoptosis via targeting FOXP4. Biosci Rep. 2020;40(6):BSR20194011. doi:10.1042/BSR20194011

178. Li W, Song Z, Jia N, Zhang C, Gao W, Wang L. microRNA-4429-5p suppresses the malignant development of colon cancer by targeting matrix metalloproteinase 16. In Vitro Cell Dev Biol Anim. 2021;57(7):715-25. doi:10.1007/s11626-021-00603-4

179. Xie Y, Xue C, Guo S, Yang L. MicroRNA-520a suppresses pathogenesis and progression of non-small-cell lung cancer through targeting the RRM2/Wnt axis. Anal Cell Pathol (Amst). 2021;2021(1):9652420. doi:10.1155/2021/9652420

180. Zhang P, Yang F, Luo Q, Yan D, Sun S. miR-1284 inhibits the growth and invasion of breast cancer cells by targeting ZIC2. Oncol Res. 2019;27(2):253. doi:10.3727/096504018X15242763477504

181. Chen H-L, Li J-J, Jiang F, Shi W-J, Chang G-Y. MicroRNA-4461 derived from bone marrow mesenchymal stem cell exosomes inhibits tumorigenesis by downregulating COPB2 expression in colorectal cancer. Biosci Biotechnol Biochem. 2020;84(2):338-46. doi:10.1080/09168451.2019.1677452

182. Jiang W, Zheng L, Yan Q, Chen L, Wang X. MiR-532-3p inhibits metastasis and proliferation of non-small cell lung cancer by targeting FOXP3. J buon. 2019;24(6):2287-93.

183. Chen W, Lu Q, Li S, Zhang X, Xue X. microRNA-1298 inhibits the malignant behaviors of breast cancer cells via targeting ADAM9. Biosci Rep. 2020;40(12):BSR20201215. doi:10.1042/BSR20201215

184. Ren Y, Li Y, Zhang W, Yang K, Li J, Hu Y, et al. Mir-4746 inhibits the proliferation of colorectal cancer cells in vitro and in vivo by targeting CCND1. Biochem Biophys Res Commun. 2022;594:153-60. doi:10.1016/j.bbrc.2022.01.063

185. Men L, Nie D, Nie H. microRNA‑577 inhibits cell proliferation and invasion in non‑small cell lung cancer by directly targeting homeobox A1. Mol Med Rep. 2019;19(3):1875-82. doi:10.3892/mmr.2019.9804

186. Chen C, Pan Y, Bai L, Chen H, Duan Z, Si Q, et al. MicroRNA-3613-3p functions as a tumor suppressor and represents a novel therapeutic target in breast cancer. Breast Cancer Res. 2021;23:1-13. doi:10.1186/s13058-021-01389-9

187. Sun J, Ye L, Shi Y, Wang X, Zhao X, Ren S, et al. MiR-6511b-5p suppresses metastasis of pMMR colorectal cancer through methylation of CD44 by directly targeting BRG1. Clin Transl Oncol. 2022;24(10):1940-53. doi:10.1007/s12094-022-02845-4

188. Guo T, Zheng C, Wang Z, Zheng X. miR‑584‑5p regulates migration and invasion in non‑small cell lung cancer cell lines through regulation of MMP‑14. Mol Med Rep. 2019;19(3):1747-52. doi:10.3892/mmr.2019.9813

189. Wu J, Miao J, Ding Y, Zhang Y, Huang X, Zhou X, et al. MiR-4458 inhibits breast cancer cell growth, migration, and invasiveness by targeting CPSF4. Biochem Cell Biol. 2019;97(6):722-30. doi:10.1139/bcb-2019-0008

190. Hao X, Su A. MiR-590 suppresses the progression of non-small cell lung cancer by regulating YAP1 and Wnt/β-catenin signaling. Clin Transl Oncol. 2022:1-10. doi:10.1007/s12094-021-02713-7

191. Zhang H-B, Shen B, Ma Z-C, Xu Y-Y, Lou Y-L, Chen M. MiR-593-5p inhibited proliferation and migration of lung adenocarcinoma by targeting ICAM-1. Eur Rev Med Pharmacol Sci. 2020;24(8). doi:10.26355/eurrev_202004_21010

192. Ke B, Huang Y, Gong Y, Zhong H, Shi L. Overexpression of microRNA-611 inhibits TGF-β-induced epithelial-mesenchymal transition and migration in lung cancer cells through MAPKAP1. Cell Signal. 2024;123:111357. doi:10.1016/j.cellsig.2024.111357

193. Wang W, Cao R, Su W, Li Y, Yan H. miR-655-3p inhibits cell migration and invasion by targeting pituitary tumor-transforming 1 in non-small cell lung cancer. Biosci Biotechnol Biochem. 2019;83(9):1703-8. doi:10.1080/09168451.2019.1617109

194. Pan H, Pan Z, Guo F, Meng F, Zu L, Fan Y, et al. MicroRNA-1915-3p inhibits cell migration and invasion by targeting SET in non-small-cell lung cancer. BMC cancer. 2021;21:1-16. doi:10.1186/s12885-021-08961-8

195. Li P, Li Y, Bai S, Zhang Y, Zhao L. miR-4732-3p prevents lung cancer progression via inhibition of the TBX15/TNFSF11 axis. Epigenomics. 2023;15(4):195-207. doi:10.2217/epi-2023-0009

196. Xiong J, Xing S, Dong Z, Niu L, Xu Q, Li Y, et al. miR‑654‑3p suppresses cell viability and promotes apoptosis by targeting RASAL2 in non‑small‑cell lung cancer. Mol Med Rep. 2021;23(2):1-. doi:10.3892/mmr.2020.11763

197. Zhou S, Han H, Yang L, Lin H. MiR-1-3p targets CENPF to repress tumor-relevant functions of gastric cancer cells. BMC Gastroenterol. 2022;22(1):145. doi:10.1186/s12876-022-02203-2

198. Wang L, Wang W, Wu Y. MicroRNA‑26b acts as an antioncogene and prognostic factor in cervical cancer. Oncol Lett. 2019;17(3):3418-24. doi:10.3892/ol.2019.9965

199. Yang H, Shen C. MicroRNA-29c induces G1 arrest of melanoma by targeting CDK6. J BUON. 2019;24(2):819-25.

200. Borchardt H, Ewe A, Morawski M, Weirauch U, Aigner A. miR24–3p activity after delivery into pancreatic carcinoma cell lines exerts profound tumor-inhibitory effects through distinct pathways of apoptosis and autophagy induction. Cancer Lett. 2021;503:174-84. doi:10.1016/j.canlet.2021.01.018

201. Fan Y, Shi Y, Lin Z, Huang X, Li J, Huang W, et al. miR-9-5p suppresses malignant biological behaviors of human gastric cancer cells by negative regulation of TNFAIP8L3. Dig Dis Sci. 2019;64:2823-9. doi:10.1007/s10620-019-05626-2

202. Wang A, Xu Q, Sha R, Bao T, Xi X, Guo G. MicroRNA‑29a inhibits cell proliferation and arrests cell cycle by modulating p16 methylation in cervical cancer. Oncol Lett. 2021;21(4):1-. doi:10.3892/ol.2021.12533

203. Zhang Z, Yang N. MiR-33a-5p inhibits the growth and metastasis of melanoma cells by targeting SNAI2. Neoplasma. 2020;67(4). doi:10.4149/neo_2020_190823N811

204. Wang L, Li M, Chen F. microRNA-26a represses pancreatic cancer cell malignant behaviors by targeting E2F7. Discov Oncol. 2021;12:1-11. doi:10.1007/s12672-021-00448-z

205. Hu X, Liu H, Li C. MiRNA-19b-3p downregulates the endothelin B receptor in gastric cancer cells to prevent angiogenesis and proliferation. Acta Biochim Pol. 2023;70(2):363-70. doi:10.18388/abp.2020_6595

206. Liu Y-J, Zhou H-G, Chen L-H, Qu D-C, Wang C-J, Xia Z-Y, et al. MiR-32-5p regulates the proliferation and metastasis of cervical cancer cells by targeting HOXB8. Eur Rev Med Pharmacol Sci. 2019;23(1). doi:10.26355/eurrev_201901_16752

207. Tian P, Tao L, Wang Y, Han X. MicroRNA‐127 inhibits the progression of melanoma by downregulating delta‐like homologue 1. Biomed Res Int. 2020;2020(1):8523465.

208. Su X, Lai T, Tao Y, Zhang Y, Zhao C, Zhou J, et al. miR-33a-3p regulates METTL3-mediated AREG stability and alters EMT to inhibit pancreatic cancer invasion and metastasis. Sci Rep. 2023;13(1):13587. doi:10.1038/s41598-023-39506-7

209. Jiang W, Meng K, Sheng G, Yang T. MicroRNA-24 inhibits the proliferation, migration and invasion and enhances chemosensitivity of human gastric cancer by targeting DND1. J buon. 2020;25:1001-6.

210. Wei H, Wang X, Niu X, Jiao R, Li X, Wang S. miR‑34c‑5p targets Notch1 and suppresses the metastasis and invasion of cervical cancer. Mol Med Rep. 2021;23(2):1-. doi:10.3892/mmr.2020.11759

211. Wu S, Han M, Zhang C. Overexpression of microRNA-130a represses uveal melanoma cell migration and invasion through inactivation of the Wnt/β-catenin signaling pathway by downregulating USP6. Cancer Gene Ther. 2022;29(7):930-9. doi:10.1038/s41417-021-00377-7

212. Lian Y, Jiang D, Sun J. Tumor suppressive role of miR-33a-5p in pancreatic ductal adenocarcinoma cells by directly targeting RAP2A. Cell Mol Biol Lett. 2021;26(1):24. doi:10.1186/s11658-021-00265-w

213. Jiang H, Liu Z-N, Cheng X-H, Zhang Y-F, Dai X, Bao G-M, et al. MiR-29c suppresses cell invasion and migration by directly targeting CDK6 in gastric carcinoma. Eur Rev Med Pharmacol Sci. 2019;23(18). doi:10.26355/eurrev_201909_19006

214. Huang C, Qin X, Zhao N, Jin H, Zhang S, Yang H. MicroRNA-100 functions as a tumor suppressor in cervical cancer via downregulating the SATB1 expression and regulating AKT/mTOR signaling pathway and epithelial-to-mesenchymal transition. Oncol Lett. 2020;20(2):1336-44. doi:10.3892/ol.2020.11686

215. Zhang X-H, Xin Z-M. MiR-135b-5p inhibits the progression of malignant melanoma cells by targeting RBX1. Eur Rev Med Pharmacol Sci. 2020;24(3). doi:10.26355/eurrev_202002_20188

216. Wang Y, Zheng Y, Chen Q, Dai Y, Li T. MicroRNA-139 inhibits pancreatic-cancer carcinogenesis by suppressing RalB via the Ral/RAC/PI3K pathway. Arch Biochem Biophys. 2021;704:108719. doi:10.1016/j.abb.2020.108719

217. Sun K-k, Shen X-j, Yang D, Gan M-q, Liu G, Zhang Y-f, et al. MicroRNA-31 triggers G2/M cell cycle arrest, enhances the chemosensitivity and inhibits migration and invasion of human gastric cancer cells by downregulating the expression of zeste homolog 2 (ZH2). Arch Biochem Biophys. 2019;663:269-75. doi:10.1016/j.abb.2019.01.023

218. Gui Y, Wang L, Huang Z. MiR-137 inhibits cervical cancer progression via down-modulating Notch1 and inhibiting the PI3K/AKT/mTOR signaling pathway. Transl Cancer Res. 2021;10(8):3748. doi:10.21037/tcr-21-1049

219. Yang C, Xia Z, Zhu L, Li Y, Zheng Z, Liang J, et al. MicroRNA-139-5p modulates the growth and metastasis of malignant melanoma cells via the PI3K/AKT signaling pathway by binding to IGF1R. Cell Cycle. 2019;18(24):3513-24. doi:10.1080/15384101.2019.1690881

220. Zhu J, Zhou L, Wei B, Qian Z, Wang J, Hui H, et al. miR‑142‑5p inhibits pancreatic cancer cell migration and invasion by targeting PIK3CA. Mol Med Rep. 2020;22(3):2085-92. doi:10.3892/mmr.2020.11251

221. Chen D-D, Cheng J-T, Chandoo A, Sun X-W, Zhang L, Lu M-D, et al. microRNA-33a prevents epithelial-mesenchymal transition, invasion, and metastasis of gastric cancer cells through the Snail/Slug pathway. Am J Physiol Gastrointest Liver Physiol. 2019;317(2):G147-G60. doi:10.1152/ajpgi.00284.2018

222. Yuan M, Zhao S, Chen R, Wang G, Bie Y, Wu Q, et al. MicroRNA‑138 inhibits tumor growth and enhances chemosensitivity in human cervical cancer by targeting H2AX. Exp Ther Med. 2020;19(1):630-8. doi:10.3892/etm.2019.8238

223. Zhao G, Yin Y, Zhao B. miR‐140‐5p is negatively correlated with proliferation, invasion, and tumorigenesis in malignant melanoma by targeting SOX4 via the Wnt/β‐catenin and NF‐κB cascades. J Cell Physiol. 2020;235(3):2161-70. doi:10.1002/jcp.29122

224. Xie F, Li C, Zhang X, Peng W, Wen T. MiR-143-3p suppresses tumorigenesis in pancreatic ductal adenocarcinoma by targeting KRAS. Biomed Pharmacother. 2019;119:109424. doi:10.1016/j.biopha.2019.109424

225. Yong H, Fu J, Gao G, Shi H, Zheng D, Zhou X. MiR-34a suppresses the proliferation and invasion of gastric cancer by modulating PDL1 in the immune microenvironment. Mol Cell Probes. 2020;53:101601. doi:10.1016/j.mcp.2020.101601

226. Guo J, Tang T, Li J, Yang Y, Quan Y, Zhang L, et al. Overexpression of MicroRNA 142-5p suppresses the progression of cervical cancer through targeting phosphoinositol-3-kinase adaptor protein 1 expression. Mol Cell Biol. 2021;41(6):e00363-20. doi:10.1128/MCB.00363-20

227. DiSano JA, Huffnagle I, Gowda R, Spiegelman VS, Robertson GP, Pameijer CR. Loss of miR-155 upregulates WEE1 in metastatic melanoma. Melanoma Res. 2019;29(2):216-9. doi:10.1097/CMR.0000000000000545

228. Chen S, Xu J, Su Y, Hua L, Feng C, Lin Z, et al. MicroRNA-145 suppresses epithelial to mesenchymal transition in pancreatic cancer cells by inhibiting TGF-β signaling pathway. J Cancer. 2020;11(9):2716. doi:10.7150/jca.34902

229. Ma Q, Zhao Y, Li Z, Gao W, Xu Y, Li B, et al. MicroRNA‐34c‐5p exhibits anticancer properties in gastric cancer by targeting MAP2K1 to inhibit cell proliferation, migration, and invasion. Biomed Res Int. 2022;2022(1):7375661. doi:10.1155/2022/7375661

230. Hu C, Liu T, Zhang W, Sun Y, Jiang D, Zhang X, et al. miR‐145 inhibits aerobic glycolysis and cell proliferation of cervical cancer by acting on MYC. FASEB J. 2023;37(4):e22839. doi:10.1096/fj.202201189RR

231. Polini B, Carpi S, Doccini S, Citi V, Martelli A, Feola S, et al. Tumor suppressor role of hsa-miR-193a-3p and-5p in cutaneous melanoma. Int J Mol Sci. 2020;21(17):6183. doi:10.3390/ijms21176183

232. Altan Z, Sahin Y. miR-203 suppresses pancreatic cancer cell proliferation and migration by modulating DUSP5 expression. Mol Cell Probes. 2022;66:101866. doi:10.1016/j.mcp.2022.101866

233. Shang J-C, Yu G-Z, Ji Z-W, Wang X-Q, Xia L. MiR-105 inhibits gastric cancer cells metastasis, epithelial-mesenchymal transition by targeting SOX9. Eur Rev Med Pharmacol Sci. 2019;23(14). doi:10.26355/eurrev_201907_18429

234. Zhang H, Lu Y, Wang S, Sheng X, Zhang S. MicroRNA-152 acts as a tumor suppressor microRNA by inhibiting Krüppel-like factor 5 in human cervical cancer. Oncol Res. 2019;27(3):335. doi:10.3727/096504018X15252202178408

235. Li D, Luo C, Deng J, Xu Y, Fu S, Liu K, et al. MicroRNA 211-5p inhibits cancer cell proliferation and migration in pancreatic cancer by targeting BMP2. Aging (Albany NY). 2023;15(23):14411. doi:10.18632/aging.205320

236. Yan J, Dai L, Yuan J, Pang M, Wang Y, Lin L, et al. miR-107 inhibits the proliferation of gastric cancer cells in vivo and in vitro by targeting TRIAP1. Front Genet. 2022;13:855355. doi:10.3389/fgene.2022.855355

237. Lu X, Song X, Hao X, Liu X, Zhang X, Yuan N, et al. miR-186-3p attenuates the tumorigenesis of cervical cancer via targeting insulin-like growth factor 1 to suppress PI3K-Akt signaling pathway. Bioengineered. 2021;12(1):7079-92. doi:10.1080/21655979.2021.1977053

238. Illmer J, Zauner R, Piñón Hofbauer J, Wimmer M, Gruner S, Ablinger M, et al. MicroRNA-200b-mediated reversion of a spectrum of epithelial-to-mesenchymal transition states in recessive dystrophic epidermolysis bullosa squamous cell carcinomas. Br J Dermatol. 2024;190(1):80-93. doi:10.1093/bjd/ljad335

239. Dutta M, Das B, Mohapatra D, Behera P, Senapati S, Roychowdhury A. MicroRNA-217 modulates pancreatic cancer progression via targeting ATAD2. Life Sci. 2022;301:120592. doi:10.1016/j.lfs.2022.120592

240. Liang F, Zhang H, Qiu Y, Xu Q, Jian K, Jiang L, et al. MiR-124-5p inhibits the progression of gastric cancer by targeting MIEN1. Technol Cancer Res Treat. 2020;19:1533033820979199. doi:10.1177/1533033820979199

241. Li N, Guo X, Liu L, Wang L, Cheng R. Molecular mechanism of miR-204 regulates proliferation, apoptosis and autophagy of cervical cancer cells by targeting ATF2. Artif Cells Nanomed Biotechnol. 2019;47(1):2529-35. doi:10.1080/21691401.2019.1628038

242. Liu Y, Ruan H, Lu F, Peng H, Luan W. miR-224–5p acts as a tumour suppressor and reverses the resistance to BRAF inhibitor in melanoma through directly targeting PAK4 to block the MAPK pathway. Pathol Res Pract. 2023;249:154772. doi:10.1016/j.prp.2023.154772

243. Jingyang Z, Jinhui C, Lu X, Weizhong Y, Yunjiu L, Haihong W, et al. Mir-320b inhibits pancreatic cancer cell proliferation by targeting FOXM1. Curr Pharm Biotechnol. 2021;22(8):1106-13. doi:10.2174/1389201021999200917144704

244. Ouyang J, Song F, Li H, Yang R, Huang H. miR-126 targeting GOLPH3 inhibits the epithelial-mesenchymal transition of gastric cancer BGC-823 cells and reduces cell invasion. Eur J Histochem. 2020;64(4). doi:10.4081/ejh.2020.3168

245. Wang X, Xu J, Hua F, Wang Y, Fang G, Zhang H, et al. MiR-214-3p suppresses cervical cancer cell metastasis by downregulating THBS2. Cell Mol Biol. 2023;69(9):195-200. doi:10.14715/cmb/2023.69.9.30

246. Mohammadi M, Spotin A, Mahami-Oskouei M, Shanehbandi D, Ahmadpour E, Casulli A, et al. MicroRNA-365 promotes apoptosis in human melanoma cell A375 treated with hydatid cyst fluid of Echinococcus granulosus sensu stricto. Microb Pathog. 2021;153:104804. doi:10.1016/j.micpath.2021.104804

247. Shi J, Su Q, Han F, Chen W, Zhang D, Xu B. MiR-337 suppresses pancreatic cancer development via STAT3/Wnt/β-catenin axis. Anticancer Drugs. 2021;32(7):681-92. doi:10.1097/CAD.0000000000001044

248. Ma L, Chen X, Li C, Cheng R, Gao Z, Meng X, et al. miR‐129‐5p and‐3p co‐target WWP1 to suppress gastric cancer proliferation and migration. J Cell Biochem. 2019;120(5):7527-38. doi:10.1002/jcb.28027

249. Ding HM, Zhang H, Wang J, Zhou JH, Shen FR, Ji RN, et al. miR‑302c‑3p and miR‑520a‑3p suppress the proliferation of cervical carcinoma cells by targeting CXCL8. Mol Med Rep. 2021;23(5):1-10. doi:10.3892/mmr.2021.11961

250. Sheng H, Guo Y-H, Cao D-S, Li X-J, Zhao Y, Ding H, et al. MiR-429-5p attenuates the migration and invasion of malignant melanoma by targeting LIMK1. Eur Rev Med Pharmacol Sci. 2020;24(5). doi:10.26355/eurrev_202003_20531

251. Mou T, Xie F, Zhong P, Hua H, Lai L, Yang Q, et al. MiR-345-5p functions as a tumor suppressor in pancreatic cancer by directly targeting CCL8. Biomed Pharmacother. 2019;111:891-900. doi:10.1016/j.biopha.2018.12.121

252. He J, Ge Q, Lin Z, Shen W, Lin R, Wu J, et al. MiR-129-5p induces cell cycle arrest through modulating HOXC10/Cyclin D1 to inhibit gastric cancer progression. FASEB J. 2019. doi:10.1096/fj.201903217R

253. Zhang J, He H, Wang K, Xie Y, Yang Z, Qie M, et al. miR-326 inhibits the cell proliferation and cancer stem cell-like property of cervical cancer in vitro and oncogenesis in vivo via targeting TCF4. Ann Transl Med. 2020;8(24). doi:10.21037/atm-20-6830

254. Nguyen M-HT, Lin C-H, Liu S-M, Miyashita A, Ihn H, Lin H, et al. miR-524-5p reduces the progression of the BRAF inhibitor-resistant melanoma. Neoplasia. 2020;22(12):789-99. doi:10.1016/j.neo.2020.10.009

255. Li W, Tang N, Tao J, Zhu Z, Liu L, Fang Q, et al. MicroRNA-374 targets JAM-2 regulates the growth and metastasis of human pancreatic cancer cells. Am J Transl Res. 2019;11(10):6454.

256. Zhao M, Liu Q, Liu W, Zhou H, Zang X, Lu J. MicroRNA‑140 suppresses Helicobacter pylori‑positive gastric cancer growth by enhancing the antitumor immune response. Mol Med Rep. 2019;20(3):2484-92. doi:10.3892/mmr.2019.10475

257. Yang S, Zhang X, Sun Y, Shi J, Jiang D, Wang J, et al. MicroRNA-362-3p inhibits migration and invasion via targeting BCAP31 in cervical cancer. Front Mol Biosci. 2020;7:107. doi:10.3389/fmolb.2020.00107

258. Tong Y, Jin L. MiR‐590‐5p targets Skp2 to inhibit the growth and invasion of malignant melanoma cells. Dis Markers. 2022;2022(1):8723725. doi:10.1155/2022/8723725

259. Qiao G, Li J, Wang J, Wang Z, Bian W. miR‑381 functions as a tumor suppressor by targeting ETS1 in pancreatic cancer. Int J Mol Med. 2019;44(2):593-607. doi:10.3892/ijmm.2019.4206

260. Song M, Liu J, Zheng X, Zhou X, Feng Z, Hu W. MiR-148a-3p targets CEMIP to suppress the genesis of gastric cancer cells. Biochem Biophys Res Commun. 2021;575:42-9. doi:10.1016/j.bbrc.2021.08.039

261. Xia N, Tan W-F, Peng Q-Z, Cai H-N. MiR-374b reduces cell proliferation and cell invasion of cervical cancer through regulating FOXM1. Eur Rev Med Pharmacol Sci. 2019;23(2). doi:10.26355/eurrev_201901_16863

262. Shi X, Xu X, Shi N, Chen Y, Fu M. miR-767-3p suppresses melanoma progression by inhibiting ASF1B expression. Biochem Biophys Res Commun. 2022;627:60-7. doi:10.1016/j.bbrc.2022.08.014

263. Wan X, Guo D, Zhu Q, Qu R. microRNA-382 suppresses the progression of pancreatic cancer through the PI3K/Akt signaling pathway by inhibition of Anxa3. Am J Physiol Gastrointest Liver Physiol. 2020;319(3):G309-G22. doi:10.1152/ajpgi.00322.2019

264. Li D, Zhang Y, Li Y, Wang X, Wang F, Du J, et al. miR‐149 Suppresses the Proliferation and Metastasis of Human Gastric Cancer Cells by Targeting FOXC1. Biomed Res Int. 2021;2021(1):1503403. doi:10.1155/2021/1503403

265. Ye C, Hu Y, Wang J. MicroRNA-377 targets zinc finger E-box-binding homeobox 2 to inhibit cell proliferation and invasion of cervical cancer. Oncol Res. 2019;27(2):183. doi:10.3727/096504018X15201124340860

266. Bezrookove V, Khan I, Bhattacharjee A, Fan J, Jones R, Sharma A, et al. miR-876-3p is a tumor suppressor on 9p21 that is inactivated in melanoma and targets ERK. J Transl Med. 2024;22(1):758. doi:10.1186/s12967-024-05527-7

267. Xu B, Gong X, Zi L, Li G, Dong S, Chen X, et al. Silencing of DLEU 2 suppresses pancreatic cancer cell proliferation and invasion by upregulating microRNA‐455. Cancer Sci. 2019;110(5):1676-85. doi:10.1111/cas.13987

268. Gu J, Li X, Li H, Jin Z, Jin J. MicroRNA-198 inhibits proliferation and induces apoptosis by directly suppressing FGFR1 in gastric cancer. Biosci Rep. 2019;39(6):BSR20181258. doi:10.1042/BSR20181258

269. Zhang M, Luo H, Hui L. MiR-3619-5p hampers proliferation and cisplatin resistance in cutaneous squamous-cell carcinoma via KPNA4. Biochem Biophys Res Commun. 2019;513(2):419-25. doi:10.1016/j.bbrc.2019.03.203

270. Zhou ZG, Xu C, Dong Z, Wang YP, Duan JY, Yan CQ. MiR-497 inhibits cell proliferation and invasion ability by targeting HMGA2 in pancreatic ductal adenocarcinoma. Eur Rev Med Pharmacol Sci. 2020;24(1):122-9. doi:10.26355/eurrev_202001_19901

271. Li X, Tibenda JJ, Nan Y, Huang S-C, Ning N, Chen G-Q, et al. MiR-204-3p overexpression inhibits gastric carcinoma cell proliferation by inhibiting the MAPK pathway and RIP1/MLK1 necroptosis pathway to promote apoptosis. World J Gastroenterol. 2023;29(29):4542. doi:10.3748/wjg.v29.i29.4542

272. Li H, Wang J, Xu F, Wang L, Sun G, Wang J, et al. By downregulating PBX3, miR-526b suppresses the epithelial–mesenchymal transition process in cervical cancer cells. Future Oncol. 2019;15(14):1577-91. doi:10.2217/fon-2018-0575

273. Zhou H, Rao Y, Sun Q, Liu Y, Zhou X, Chen Y, et al. MiR-4458/human antigen R (HuR) modulates PBX3 mRNA stability in melanoma tumorigenesis. Arch Dermatol Res. 2020;312(9):665-73. doi:10.1007/s00403-020-02051-8

274. Nong K, Zhang D, Chen C, Yang Y, Yang Y, Liu S, et al. MicroRNA‑519 inhibits hypoxia‑induced tumorigenesis of pancreatic cancer by regulating immune checkpoint PD‑L1. Oncol Lett. 2020;19(2):1427-33. doi:10.3892/ol.2019.11234

275. Zhang J, Zhang J, Pang X, Chen Z, Zhang Z, Lei L, et al. MiR-205–5p suppresses angiogenesis in gastric cancer by downregulating the expression of VEGFA and FGF1. Exp Cell Res. 2021;404(2):112579. doi:10.1016/j.yexcr.2021.112579

276. Ji H, Hu N-J. MiR-613 blocked the progression of cervical cancer by targeting LETM1. Eur Rev Med Pharmacol Sci. 2020;24(12). doi:10.26355/eurrev_202006_21642

277. Liang J, Liu Y, Zhang L, Tan J, Li E, Li F. Overexpression of microRNA-519d-3p suppressed the growth of pancreatic cancer cells by inhibiting ribosomal protein S15A-mediated Wnt/β-catenin signaling. Chem Biol Interact. 2019;304:1-9. doi:10.1016/j.cbi.2019.02.026

278. Chen Z, Gao Y-J, Hou R-Z, Ding D-Y, Song D-F, Wang D-Y, et al. MicroRNA-206 facilitates gastric cancer cell apoptosis and suppresses cisplatin resistance by targeting MAPK2 signaling pathway. Eur Rev Med Pharmacol Sci. 2019;23(1). doi:10.26355/eurrev_201901_16761

279. Hu QL, Xu ZP, Lan YF, Li B. miR‐636 represses cell survival by targeting CDK6/Bcl‐2 in cervical cancer. Kaohsiung J Med Sci. 2020;36(5):328-35. doi:10.1002/kjm2.12181

280. Yu H, Gao G, Cai J, Song H, Ma Z, Jin X, et al. MiR-539 functions as a tumor suppressor in pancreatic cancer by targeting TWIST1. Exp Mol Pathol. 2019;108:143-9. doi:10.1016/j.yexmp.2019.04.012

281. Chen X, Zhang L, Song Q, Chen Z. MicroRNA-216b regulates cell proliferation, invasion and cycle progression via interaction with cyclin T2 in gastric cancer. Anticancer Drugs. 2020;31(6):623-31. doi:10.1097/CAD.0000000000000915

282. Wang M, Liu K, Zhou Z, Geng H. miR-638 suppresses cervical cancer progression by inhibiting NCAPG2 under the treatment of Tetrandrine. Histol Histopathol. 2024. doi:10.14670/HH-18-657

283. Liu Y, Wei J, Wang C, Meng Z, Luo D, Zhao X, et al. MicroRNA-543 controls pancreatic cancer development by LINC00847-microRNA-543-STK31 axis. J Gastrointest Oncol. 2022;13(6):3263. doi:10.21037/jgo-22-1017

284. Zhou X, Hu M, Ge Z. Tumor‑suppressive miR‑299‑3p inhibits gastric cancer cell invasion by targeting heparanase. Mol Med Rep. 2019;20(3):2151-8. doi:10.3892/mmr.2019.10436

285. Lv M, Ou R, Zhang Q, Lin F, Li X, Wang K, et al. MicroRNA-664 suppresses the growth of cervical cancer cells via targeting c-Kit. Drug Des Devel Ther. 2019:2371-9. doi:10.2147/DDDT.S203399

286. Wang L, Gao P, Yuan P, Zhou P, Fan H, Lin X, et al. miR-573 suppresses pancreatic cancer cell proliferation, migration, and invasion through targeting TSPAN1. Strahlenther Onkol. 2021;197:438-48. doi:10.1007/s00066-020-01728-3

287. Zhang J, Wang C, Yan S, Yang Y, Zhang X, Guo W. miR-345 inhibits migration and stem-like cell phenotype in gastric cancer via inactivation of Rac1 by targeting EPS8. Acta Biochim Biophys Sin. 2020;52(3):259-67. doi:10.1093/abbs/gmz166

288. Wu X, Liu L, Zhang H. miR‑802 inhibits the epithelial‑mesenchymal transition, migration and invasion of cervical cancer by regulating BTF3. Mol Med Rep. 2020;22(3):1883-91. doi:10.3892/mmr.2020.11267

289. Li S, Li X, Xing X, Wang L. miR-597-5p inhibits cell growth and promotes cell apoptosis by targeting ELK1 in pancreatic cancer. Hum Cell. 2020;33(4):1165-75. doi:10.1007/s13577-020-00395-x

290. Qian B, Zhang D, Tao R, Yu G, Jia B, Ye K, et al. MiR-361-5p exerts tumor-suppressing functions in gastric carcinoma by targeting syndecan-binding protein. Anticancer Drugs. 2020;31(2):131-40. doi:10.1097/CAD.0000000000000846

291. Peng X, Zhang Y, Gao J, Cai C. MiR-1258 promotes the apoptosis of cervical cancer cells by regulating the E2F1/P53 signaling pathway. Exp Mol Pathol. 2020;114:104368. doi:10.1016/j.yexmp.2020.104368

292. Li M, Li T, Ma W, Wang X, Zhao G. MicroRNA‑608 promotes apoptosis via BRD4 downregulation in pancreatic ductal adenocarcinoma. Oncol Lett. 2020;19(2):1418-26. doi:10.3892/ol.2019.11246

293. Hong D, Zang A, Wang Z, Yang L, Ren G, Zhang C, et al. Elevation of microRNA-365 impedes malignant behaviors of gastric cancer cells by inhibiting PAX6. Funct Integr Genomics. 2022;22(5):825-34. doi:10.1007/s10142-022-00858-4

294. Liu H, Li Y, Zhang J, Wu N, Liu F, Wang L, et al. Erb‑B2 Receptor Tyrosine Kinase 2 is negatively regulated by the p53‑responsive microRNA‑3184‑5p in cervical cancer cells. Oncol Rep. 2021;45(1):95-106. doi:10.3892/or.2020.7862

295. Javadrashid D, Mohammadzadeh R, Baghbanzadeh A, Safaee S, Amini M, Lotfi Z, et al. Simultaneous microRNA-612 restoration and 5-FU treatment inhibit the growth and migration of human PANC-1 pancreatic cancer cells. EXCLI J. 2021;20:160-73. doi:10.17179/excli2020-2900

296. Yin Y, Li X, Guo Z, Zhou F. MicroRNA‑381 regulates the growth of gastric cancer cell by targeting TWIST1. Mol Med Rep. 2019;20(5):4376-82. doi:10.3892/mmr.2019.10651

297. Wang Y, Li X, Wang S, Song Z, Bao Y, Zheng L, et al. miR-3929 inhibits proliferation and promotes apoptosis by downregulating cripto-1 expression in cervical cancer cells. Cytogenet Genome Res. 2021;161(8-9):425-36. doi:10.1159/000518521

298. Chen D, Wu X, Zhao J, Zhao X. MicroRNA‑634 functions as a tumor suppressor in pancreatic cancer via directly targeting heat shock‑related 70‑kDa protein 2. Exp Ther Med. 2019;17(5):3949-56. doi:10.3892/etm.2019.7433

299. Xu G, Li N, Zhang Y, Zhang J, Xu R, Wu Y. MicroRNA-383-5p inhibits the progression of gastric carcinoma via targeting HDAC9 expression. Braz J Med Biol Res. 2019;52:e8341. doi:10.1590/1414-431X20198341

300. Sui X, Sui Z. MiR-4269 suppresses the tumorigenesis and development of pancreatic cancer by targeting ZEB1/OTX1 pathway. Biosci Rep. 2020;40(6):BSR20200010. doi:10.1042/BSR20200010

301. Bai T, Liu Y, Li B. MiR-411 inhibits gastric cancer proliferation and migration through targeting SETD6. Eur Rev Med Pharmacol Sci. 2019;23(8):3344-50. doi:10.26355/eurrev_201904_17697

302. Liu P, Ding Pa, Yang J, Wu H, Wu J, Guo H, et al. MicroRNA‐431‐5p inhibits angiogenesis, lymphangiogenesis, and lymph node metastasis by affecting TGF‐β1/SMAD2/3 signaling via ZEB1 in gastric cancer. Mol Carcinog. 2024;63(7):1378-91. doi:10.1002/mc.23731

303. Zhan T, Chen M, Liu W, Han Z, Zhu Q, Liu M, et al. MiR-455-3p inhibits gastric cancer progression by repressing Wnt/β-catenin signaling through binding to ARMC8. BMC Med Genomics. 2023;16(1):155. doi:10.1186/s12920-023-01583-y

304. Chen R, Zhou S, Fang C, Ye F, Chen J, Jiang P. Targeting AURKA by microRNA-490-3p suppresses gastric cancer cell growth. Histol Histopathol. 2022. doi:10.14670/HH-18-415

305. Liu Z, Lü Y, Jiang Q, Yang Y, Dang C, Sun R. miR-491 inhibits BGC-823 cell migration via targeting HMGA2. Int J Biol Markers. 2019;34(4):364-72. doi:10.1177/1724600819874488

306. Jiang W, Yang W, Liu J, Zhao X, Lu W. Cancer-suppressing miR-520-3p gene inhibits proliferation, migration, and invasion of gastric cancer cells through targeted regulation of KLF7. Bull Cancer. 2022;109(6):631-41. doi:10.1016/j.bulcan.2022.02.005

307. Chang Y, Hao Y, Su Y, Guo J, Liu Y, Sun R, et al. MicroRNA-582-5p inhibits the progression of gastric cancer cells and their resistance to oxaliplatin by suppressing ATG7 expression. Front Oncol. 2024;14:1481266. doi:10.3389/fonc.2024.1481266

308. Zhang Z, Dai D-Q. MicroRNA-596 acts as a tumor suppressor in gastric cancer and is upregulated by promotor demethylation. World J Gastroenterol. 2019;25(10):1224. doi:10.3748/wjg.v25.i10.1224

309. Han X, Liu H, Tang X, Zhao Y. MiRNA-621 exerts tumor suppressor function in gastric adenocarcinoma by targeting AURKA/GSK-3β pathway. Acta Biochim Pol. 2021;68(1):91-8. doi:10.18388/abp.2020_5452

310. Wu Y, Ye H, Peng B, Jiang H, Tang Q, Liu Y, et al. MiR-643 functions as a potential tumor suppressor in gastric cancer by inhibiting cell proliferation and invasion via targeting TXNDC9. Ann Clin Lab Sci. 2021;51(4):494-502.

311. Wu K-Z, Zhang C-D, Zhang C, Pei J-P, Dai D-Q. miR-665 suppresses the epithelial–mesenchymal transition and progression of gastric cancer by targeting CRIM1. Cancer Manag Res. 2020:3489-501. doi:10.2147/CMAR.S241795

312. Li L, Gao J, Li J, Wang J. MiR-711 regulates gastric cancer progression by targeting CD44. Cancer Biomark. 2022;35(1):71-81. doi:10.3233/CBM-210213

313. Ge L, Wang Y, Duan Q-H, Liu S-S, Liu G-J. MicroRNA-760 acts as a tumor suppressor in gastric cancer development via inhibiting G-protein-coupled receptor kinase interacting protein-1 transcription. World J Gastroenterol. 2019;25(45):6619. doi:10.3748/wjg.v25.i45.6619

314. Zhang X, Dai X, Zhao X, Wang J, Dou J, Zhuang H, et al. MiR-874-3p represses the migration and invasion yet promotes the apoptosis and cisplatin sensitivity via being sponged by long intergenic non-coding RNA 00922 (LINC00922) and targeting Glycerophosphodiester Phosphodiesterase Domain Containing 5 (GDPD5) in gastric cancer cells. Bioengineered. 2022;13(3):7082-104. doi:10.1080/21655979.2022.2045831

315. Liu S, Gong Y, Xu XD, Shen H, Gao S, Bao HD, et al. MicroRNA‐936/ERBB4/Akt axis exhibits anticancer properties of gastric cancer through inhibition of cell proliferation, migration, and invasion. Kaohsiung J Med Sci. 2021;37(2):111-20. doi:10.1002/kjm2.12304

316. Li Y, Qin C. MiR-1179 inhibits the proliferation of gastric cancer cells by targeting HMGB1. Hum Cell. 2019;32(3):352-9. doi:10.1007/s13577-019-00244-6

317. Xu X-c, Zhang W-b, Li C-x, Gao H, Pei Q, Cao B-w, et al. Up-regulation of miR-1915 inhibits proliferation, invasion, and migration of Helicobacter pylori-infected gastric cancer cells via targeting RAGE. Yonsei Med J. 2019;60(1):38-47. doi:10.3349/ymj.2019.60.1.38

318. Cai A, Xia P, Zhou X, He Y, Lv J. MiR-1275 targeting SPARC promotes gambogic acid-induced inhibition of gastric cancer. Biochem Genet. 2023;61(6):2481-95. doi:10.1007/s10528-023-10381-1

319. Tian M, Jiang M, Bi Y, Wang B. miR-1286, a tumor suppressor of gastric cancer, serves as a promising biomarker for screening gastric cancer from gastritis. Biochem Genet. 2024:1-13. doi:10.1007/s10528-023-10618-z

320. Zhang X-Y, Xu Y-Y, Chen W-Y. MicroRNA-1324 inhibits cell proliferative ability and invasiveness by targeting MECP2 in gastric cancer. Eur Rev Med Pharmacol Sci. 2020;24(9). doi:10.26355/eurrev_202005_21165

321. Zhang F, Wang G, Yan W, Jiang H. MiR-4268 suppresses gastric cancer genesis through inhibiting keratin 80. Cell Cycle. 2022;21(19):2051-64. doi:10.1080/15384101.2022.2085351

322. He H, Wu W, Sun Z, Chai L. MiR-4429 prevented gastric cancer progression through targeting METTL3 to inhibit m6A-caused stabilization of SEC62. Biochem Biophys Res Commun. 2019;517(4):581-7. doi:10.1016/j.bbrc.2019.07.058

323. Miao Y, Li Q, Sun G, Wang L, Zhang D, Xu H, et al. MiR‐5683 suppresses glycolysis and proliferation through targeting pyruvate dehydrogenase kinase 4 in gastric cancer. Cancer Med. 2020;9(19):7231-43. doi:10.1002/cam4.3344

324. Zhou W, Ding X, Jin P, Li P. miR-6838-5p affects cell growth, migration, and invasion by targeting GPRIN3 via the Wnt/β-catenin signaling pathway in gastric cancer. Pathobiology. 2020;87(6):327-37. doi:10.1159/000511691

325. Meng M, Guan G, Liu X, Sun W, Cui X, Fu S, et al. miR-7160 inhibits gastric cancer cell proliferation and metastasis by silencing SIX1. J Genet. 2024;103(2):27.

326. Pradhan R, Singhvi G, Dubey SK, Gupta G, Dua K. MAPK pathway: a potential target for the treatment of non-small-cell lung carcinoma. Future Med Chem. 2019;11(8):793-5. doi:10.4155/fmc-2018-0468
